# Secrets of the Hospital Underbelly: Patterns of Abundance of Antimicrobial Resistance Genes in Hospital Wastewater Vary by Specific Antimicrobial and Bacterial Family

**DOI:** 10.3389/fmicb.2021.703560

**Published:** 2021-09-10

**Authors:** Meghan R. Perry, Hannah C. Lepper, Luke McNally, Bryan A. Wee, Patrick Munk, Amanda Warr, Barbara Moore, Pota Kalima, Carol Philip, Ana Maria de Roda Husman, Frank M. Aarestrup, Mark E. J. Woolhouse, Bram A. D. van Bunnik

**Affiliations:** ^1^Usher Institute, University of Edinburgh, Edinburgh, United Kingdom; ^2^NHS Lothian Infection Service, Edinburgh Clinical Infection Research Group, Edinburgh, United Kingdom; ^3^Centre for Inflammation Research, University of Edinburgh, Edinburgh, United Kingdom; ^4^Centre for Synthetic and Systems Biology, School of Biological Sciences, University of Edinburgh, Edinburgh, United Kingdom; ^5^School of Biological Sciences, Institute of Evolutionary Biology, University of Edinburgh, Edinburgh, United Kingdom; ^6^National Food Institute, Technical University of Denmark, Kongens Lyngby, Denmark; ^7^Roslin Institute, University of Edinburgh, Edinburgh, United Kingdom; ^8^National Institute for Public Health and the Environment (RIVM), Bilthoven, Netherlands

**Keywords:** antimicrobial resistance, metagenomics, hospital waste water, surveillance, environmental risk, resistance dissemination, antibiotic usage

## Abstract

**Background:** Hospital wastewater is a major source of antimicrobial resistance (AMR) outflow into the environment. This study uses metagenomics to study how hospital clinical activity impacts antimicrobial resistance genes (ARGs) abundances in hospital wastewater.

**Methods:** Sewage was collected over a 24-h period from multiple wastewater collection points (CPs) representing different specialties within a tertiary hospital site and simultaneously from community sewage works. High throughput shotgun sequencing was performed using Illumina HiSeq4000. ARG abundances were correlated to hospital antimicrobial usage (AMU), data on clinical activity and resistance prevalence in clinical isolates.

**Results:** Microbiota and ARG composition varied between CPs and overall ARG abundance was higher in hospital wastewater than in community influent. ARG and microbiota compositions were correlated (Procrustes analysis, *p*=0.014). Total antimicrobial usage was not associated with higher ARG abundance in wastewater. However, there was a small positive association between resistance genes and antimicrobial usage matched to ARG phenotype (IRR 1.11, CI 1.06–1.16, *p*<0.001). Furthermore, analyzing carbapenem and vancomycin resistance separately indicated that counts of ARGs to these antimicrobials were positively associated with their increased usage [carbapenem rate ratio (RR) 1.91, 95% CI 1.01–3.72, *p*=0.07, and vancomycin RR 10.25, CI 2.32–49.10, *p*<0.01]. Overall, ARG abundance within hospital wastewater did not reflect resistance patterns in clinical isolates from concurrent hospital inpatients. However, for clinical isolates of the family *Enterococcaceae* and *Staphylococcaceae*, there was a positive relationship with wastewater ARG abundance [odds ratio (OR) 1.62, CI 1.33–2.00, *p*<0.001, and OR 1.65, CI 1.21–2.30, *p*=0.006 respectively].

**Conclusion:** We found that the relationship between hospital wastewater ARGs and antimicrobial usage or clinical isolate resistance varies by specific antimicrobial and bacterial family studied. One explanation, we consider is that relationships observed from multiple departments within a single hospital site will be detectable only for ARGs against parenteral antimicrobials uniquely used in the hospital setting. Our work highlights that using metagenomics to identify the full range of ARGs in hospital wastewater is a useful surveillance tool to monitor hospital ARG carriage and outflow and guide environmental policy on AMR.

## Introduction

In response to the antimicrobial resistance (AMR) crisis, a challenge for the research and medical communities is understanding the flow of AMR between different environmental niches (Woolhouse et al., 2015) and deciding where to focus surveillance and interventions to inform effective policies and action (Laxminarayan et al., 2016). There is an increasing interest in the contribution of hospital wastewater to AMR in the environment. Sewage treatment does not completely eradicate antimicrobial resistance genes (ARGs) and thus ARGs can enter the food chain through water and the use of sewage sludge in agriculture (Woolhouse and Ward, 2013; Woolhouse et al., 2015). As a complex matrix representing human bodily waste the potential of community sewage as a surveillance tool to monitor the global epidemiology of AMR has recently been explored (Hendriksen et al., 2019; Aarestrup and Woolhouse, 2020).

Hospitals are epidemiologically important nodal points for concentrated antimicrobial consumption and are sources of resistant pathogens (Versporten et al., 2018). Secondary care surveillance, guided by national and international policies, is based on passive reporting of phenotypic and molecular laboratory results for specific pathogens or from screening samples on specific high risk patients (Tornimbene et al., 2018; Department of Health and Social Care, 2019). These methods do not represent the full impact of antimicrobial use and inpatient activity on AMR carriage within a hospital and thus risk of transmission. Nor do they capture all pertinent ARGs. As hospital wastewater contains inpatient bodily waste, we hypothesized that it could be used as a representation of hospital inpatient carriage of AMR and as such may be a useful surveillance tool.

Many previous studies have identified key pathogens and resistant genes in hospital wastewater and attempts have been made to correlate resistance of specific organisms from hospital clinical isolates with hospital wastewater isolates with conflicting results (Talebi et al., 2008; Tuméo et al., 2008; Yang et al., 2009; Santoro et al., 2012; Drieux et al., 2016; Maheshwari et al., 2016). In this study, we apply the technique of metagenomics to hospital waste water (Hendriksen et al., 2019), with cross-sectional sampling of waste water from different hospital departments. The use of metagenomics in hospital waste water is increasingly applied to understand the resistance profile of hospitals (Subirats et al., 2016; Rowe et al., 2017; Ekwanzala et al., 2020; Petrovich et al., 2020; Kutilova et al., 2021). Combining metagenomics and multiple sampling sites allowed us to test hypotheses about what factors may drive patterns in resistance abundance in hospital waste water. We investigated whether clinical activity, such as antimicrobial usage and patient length of stay, impacts resistance abundance in hospital waste water. We also tested our hypothesis that resistance in hospital patients is correlated with the abundance of resistance genes within that department’s waste water.

## Materials and Methods

### Sewage Collection and Antibiotic Residue Analysis

Sampling was performed in June 2017 on eight wastewater collection points (CP) in the Western General Hospital, Edinburgh. Each sampling point represented a different clinical departments, identified to capture the effluent from the majority of the hospital (Supplementary Figure S1). No treatment was applied to hospital effluent prior to discharge into the main sewerage network. Using composite sampling machines, 100ml of wastewater was sampled every 15min over a 24-h period thus aiming to collect a representative sample of waste from the hospital inpatient population. Simultaneously, a 24-h time proportional sample was collected at the inflow site to Seafield community sewage works (hereafter “Seafield”), which serves a population equivalent of 760,000 from Edinburgh and the Lothians. Samples were transported from the site on dry ice and stored at −80°C. Antibiotic residue analysis was performed on 1L of composite hospital wastewaters and 1L of domestic sewage using LC-MS/MS as previously described (Berendsen et al., 2015; Hendriksen et al., 2019).

### DNA Extraction and Analysis

DNA was extracted from sewage by pelleting using the QIAamp Fast DNA Stool mini kit with an optimized protocol as previously described (Knudsen et al., 2016) and sequenced on the HiSeq4000 platform (Illumina) using 2×150bp paired-end sequencing. The concentrations of gDNA in nanograms per microliter per sample measured by Qubit can be found in Supplementary Table S2. All samples used in this analysis met the minimum quality requirements genomic DNA biomass used by the sequencing firm BGI Genomics. The taxonomic origin of paired reads were assigned using Kraken2 (Wood and Salzberg, 2014) to the standard database, a database of representative bacterial genomes and a database of known vector sequences, UniVec_Core (downloaded 9th April 2019). Taxonomic assignments were summarized at the genus level using kraken-biom (Dabdoub, 2019). One sample, CP2, was heavily contaminated with *Pseudomonas*, likely from the *Pseudomonas fluorescens* species group. In CP2 52.9% of reads aligned to *Pseudomonas* genus OTUs, compared to 7.2% on average for other hospital sites. We therefore removed results from this site from further analysis. We used KMA version 1.2.12 (Clausen et al., 2018) to assign the paired and singleton reads to a database consisting of ResFinder reference genes (Zankari et al., 2012; downloaded 5th of September, 2019). KMA uses *k*-mer seeding followed by the Needleman-Wunsch sequence alignment algorithm to align the rest of the read from these *k*-mer seeds. ResFinder is a reference database of AMR genes. The following flags were used: “-mem_mode -ef -1t1 -cge -nf -shm 1 -t 1.” Reads mapping to the human reference genome (GCA_000001405.15) were removed prior to submission to public sequence databases according to the protocol used in the Human Microbiome Project (Sherry, 2011; Human Microbiome Project, 2021).

### Data Collection

Data was collected on clinical isolates from the week surrounding the hospital wastewater sampling to represent pathogens in hospital inpatients. All types of clinical isolate were included (including fecal, urine, skin, indwelling plastic, and fluid and tissue) but duplicate samples from the same patient within a 48-h period were excluded. Antimicrobial usage was collated from weekly pharmacy issues to each ward over the 3months prior to sampling and presented as defined daily dose per 100 occupied bed days (DDD/100OBDs). Pharmacy issues for prescriptions for outpatient use and for theaters were excluded.

### Data Analysis

All statistical analysis and plots were produced using R version 3.6.0. The abundance of ARGs and bacterial genera were calculated as Reads Per Kilobase of transcript per Million mapped bacterial reads (RPKM; Munk et al., 2018). This measure is frequently used for metagenomic data, and normalizes the read hit count with respect to the gene length in base pairs and the total number of bacterial reads. Principal coordinate analysis (PCoA; e.g., Borcard et al., 2018) was conducted on Bray-Curtis dissimilarity matrices were determined using Hellinger transformation of the RPKM. Resistance genes from the ResFinder database were grouped into clusters with 90% sequence homology. The top 50 ARGs were visualized using a heatmap and gene-wise and collection point dendrograms as previously described (Hendriksen et al., 2019). Procrustes analysis was used to test the association between the resistome and bacteriome dissimilarities.

### Correlation Between Inpatient Activity and ARG Abundance

The source of variance in the abundance of ARGs between the collection points was investigated using a multilevel Poisson model with the dependent variable as counts of ARG reads at each collection point aggregated at the 90% homology cluster level. We used an offset term with the log of the average gene-length per cluster in the ResFinder database, multiplied by the total bacterial reads per collection point. Random effects of collection point, 70% sequence homology cluster, and observation were included in the model, the latter to model the over dispersion inherent to count data (Harrison, 2014).

In the main model, we accounted for co- and cross-resistance by fitting both a measure of direct selection for resistance (effect of department-level usage of antimicrobials on ARGs that confer resistance to those antimicrobials) and indirect selection [effect of total department-level antimicrobial usage (AMU) on ARG abundance]. In a second set of three models, we tested the association between resistance genes and antimicrobial usage of three specific antimicrobials of interest chosen to represent parenteral antimicrobials only used in a hospital setting (carbapenems, vancomycin) and an antimicrobial widely used in both community and hospital (amoxicillin). We use a Bonferroni correction on *p* values of these additional tests to account for increased risk of type I error. We used all antimicrobial resistance phenotypes suggested for any gene in a 90% homology cluster from either the ResFinder or STARAMR (National Microbiology Laboratory, 2021) databases. The average length of stay per department was also used to assess the role of clinical activity on sewage resistance abundance in the main model. The fixed effects structure of the main model was further adjusted using AIC minimizing methods, assessing whether any interaction effect should be included.

To assess the relationship between AMR in clinical isolates and ARG abundance in hospital wastewater a binomial generalized linear mixed effects model was used including random effects for site, the class of the antimicrobial used to test the isolates, and for the species of the isolate to control for inter-species heterogeneity. Two fixed effects were estimated for the log RPKM of all resistance genes in the sewage that had the same resistance phenotype as the isolates: one for isolates that were urinary or fecal, and a second for all other isolate types, due to the different dynamics of inpatient bodily waste being represented in the wastewater system. Using separate binomial regression models, we accounted for heterogeneity between the taxonomic family of the isolates in the relationship between AMR in clinical isolates and sewage ARGs. As some families were rarely tested, the sample size was too small for this heterogeneity to be assessed in a single model. Therefore, the three most frequently isolated families were assessed (*Enterobacteriaceae*, *Enterococcaceae*, and *Staphylococcaceae*), with the log RPKM of phenotypically matched resistance genes as the only model effect. A Bonferroni correction was used to adjust the *p* values of the effects of these models to account for multiple testing. A similar model was used to evaluate the relationship between AMU and AMR in clinical isolates.

### Ethics

This study was conducted following approval from NHS Lothian Research and Development Committee under the sponsorship of University of Edinburgh. There was no direct patient contact and therefore the study did not require ethical board approval.

## Results

The hospital departments served by the wastewater collection points differed by pattern of antimicrobial use (Table 1; Supplementary Table S2) and resistance in the 181 clinical isolates identified in the week surrounding wastewater sampling (Supplementary Figure S3).

**Table 1:** Demographics of hospital collection points.

Collection point	Specialties	No. of wards	No. of pts	Average length of stay in days (SD)	Average age in years (SD)	DDD per 100 OBDs	No. of clinical isolates
CP1	Cardiology, Urology	3	46	4.9 (0.8)	62.6 (2.2)	123.7	19
CP3	Oncology, Hematology	7	67	3.7 (2.7)	62.1 (1.0)	200.5	27
CP4	Acute receiving unit	5	35	0.9 (0.7)	70.5 (2.3)	325.8	45
CP5	Neuroscience	3	59	3.3 (1.1)	53.5 (2.1)	73.5	8
CP6	Intensive care, Surgery, Medicine	3	70	7.6 (2.5)	66.6 (1.7)	223.8	17
CP7	Infectious Diseases, Surgery, Medicine	6	105	6.1 (3.2)	63.5 (0.8)	148.1	20
CP8	Respiratory, Medicine for the Elderly, Urology, Surgical High Dependency	6	133	12.8 (9.0)	69.0 (1.0)	116.4	25

SD only represents SD of the average age and length of stay per week. Antimicrobial usage from previous 3months does not include antibiotics issued for outpatient prescriptions or in theaters. Clinical isolates are from inpatients in the week surrounding wastewater collection. pts, patients; DDD, defined daily dose; OBDs, occupied bed days; and SD, standard deviation.

### Metagenomics of Wastewater

An average read pair count of 38.4 million (range 35.7–39.2 million) was obtained with an average of 62% (range 52–73%) of reads allocated to bacteria from the seven hospital wastewater samples and one community sewage sample.^1^ An average of 0.25% of reads mapped to ARGs in the seven hospital wastewater samples vs. 0.1% from Seafield (Supplementary Table S1).

**Figure 1:**
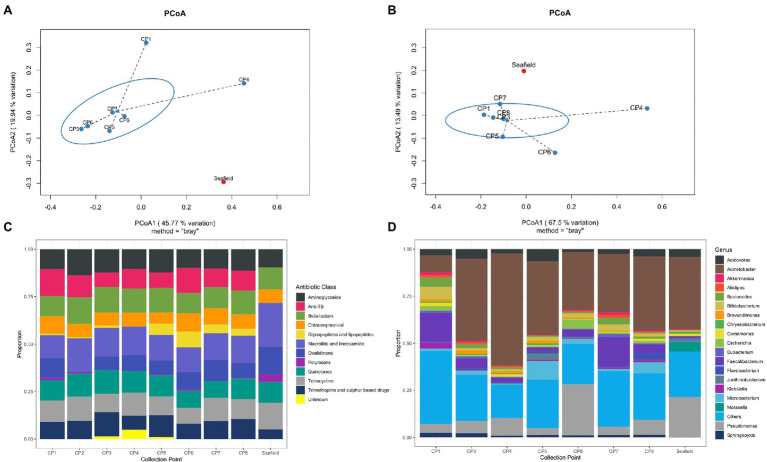
Hospital wastewater and community sewage resistome and microbiome abundance composition. **(A)** Principal coordinate analyses (PCoA) of resistome based on Bray-Curtis dissimilarity. The percentage of variation explained is noted on the axis labels. **(B)** Principal coordinate analyses for the microbiome. **(C)** Relative abundance of antimicrobial resistance genes (ARGs) by antimicrobial class. **(D)** Relative abundance of the 19 most abundant bacterial genera in the wastewater and sewage microbiome. CP, collection point within hospital; Seafield, community sewage works; and TB, tuberculosis.

One thousand, one hundred and fifty-four unique bacterial genera were detected across all samples (range 1,151–1,154 genera per sample; Supplementary Table S2). The top 19 genera accounted for >70% of bacterial abundance in all samples (Figure 1D). The most predominant genera were *Pseudomonas* and *Acinetobacter*, mainly environmental species such as *Pseudomonas fluorescens*, *Acinetobacter johnsonii*, likely representing bacteria usually present in the hospital pipes. When compared with Seafield, there was a difference in diversity in the hospital samples with a higher predominance of gut associated bacteria including *Faecalibacterium*, *Bacteroides*, *Bifidobacterium*, and *Escherichia* (Figures 1B,D).

**Figure 2:**
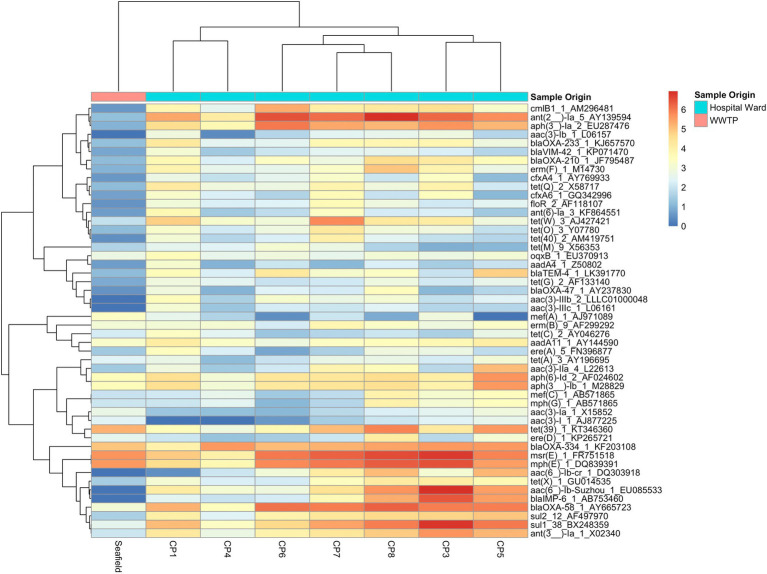
Heat map of 50 most abundant ARGs. Relative abundance of ARGs (RPKM) were log transformed and both ARGs and CPs were clustered using complete-linkage clustering. For ARGs clustering was based on Pearson correlation coefficients, for collection points clustering was based on the BC-dissimilarity matrix (Figure 1) which uses all genes.

Antimicrobial resistance gene abundance and composition varied across different hospital collection points and Seafield (Figures 1A,C, 2; Supplementary Figures S4, S6). Apart from the wastewater collected at CP4, which represents the acute receiving unit with patients directly admitted from the community, ARG abundance from hospital wastewater was higher than ARG abundance in Seafield (Figure 2; Supplementary Figure S4). ARG composition was strongly correlated with bacterial genus level composition (Procrustes, *p*=0.014; Supplementary Figure S6).

We detected 502 different resistance genes belonging to 10 different antimicrobial classes (Supplementary Table S3) but over 65% of the sample resistomes were composed of the 15 most abundant genes (Supplementary Figure S6), mainly belonging to the aminoglycoside and macrolide antimicrobial classes (Figure 1C). Key ARGs of interest to infection control including *bla*OXA, *bla*IMP, and genes of the *vanA* cluster were identified.

### Inpatient Activity and ARG Abundance

**Figure 3:**
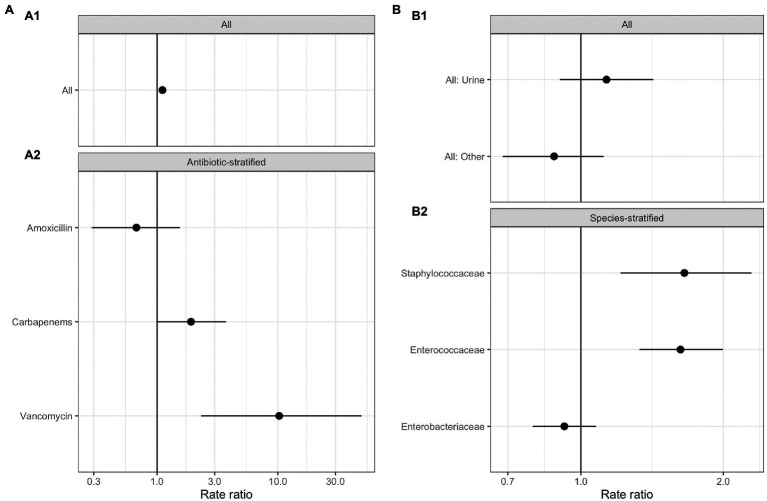
Generalized linear mixed effects models for the relationship between antimicrobial resistance gene abundance, hospital department antibiotic consumption rates, and hospital department rates of resistance in clinical isolates. **(A)** Effect of antimicrobial usage (AMU) measured in defined daily dose per 100 occupied bed days (DDD/100 OBDs) on ARG abundance. **(A1)** The main model, with a single coefficient for all resistance phenotypes. **(A2)** Separate models with coefficients for each antimicrobial. **(B)** Association between antimicrobial resistance gene abundance in the sewage and clinical resistance rates. **(B1)** Main model, with a single coefficient for all clinical isolate taxonomic family, stratified by sample type – urine or fecal samples (All: Urine), and for resistance genes and any other sample source (All: Other). **(B2)** Separate models with coefficients for each isolate taxonomic family.

No significant relationships were observed between total antimicrobial usage or length of stay and the abundance of ARGs in sewage (Figure 3; Supplementary Table S5). This result indicates there was no evidence for indirect selection or for the impact of transmission among hospital patients on ARG abundance in sewage when all resistance phenotypes were modeled. There was a significant positive effect of increased phenotypically-matched antimicrobial usage on resistance gene abundance, indicating support for a small role of direct selection (IRR 1.11, CI 1.06–1.16, *p*<0.001). AIC comparison of fixed effect structures for the model indicated that no interaction effects improved model fit.

We next analyzed data on the association between carbapenem, vancomycin, and amoxicillin usage and ARGs conferring resistance to these specific antimicrobials in three separate models (Figure 3A; Supplementary Table S5). We found positive associations that were significant between vancomycin ARGs and vancomycin usage (IRR 10.25, CI 2.32–49.10, *p*<0.001) and showed a trend toward significance between carbapenem ARG abundance and carbapenem antimicrobial usage (IRR 1.91, CI 1.01–3.72, *p*=0.07). No evidence for an association between amoxicillin usage and amoxicillin ARGs was identified. We omitted the observation-level random effect from vancomycin model due to singular model fits, so overdispersion was not accounted for.

Antimicrobial resistance gene abundance at a class level within hospital wastewater did not reflect resistance patterns in clinical isolates when all the data was analyzed in one model (Figure 3B; Supplementary Table S6). There was no difference between the relationship of isolates from urine and fecal samples with ARG abundance and isolates from other sample types, e.g., skin, which we expect to enter the wastewater system at different rates *via* sinks and showers. We next separately modeled the three most frequently isolated taxonomic families (Figure 3; Supplementary Table S6). *Enterococcaceae* and *Staphylococcaceae* had a significant positive association with the abundance of ARGs conferring resistance to the same antimicrobial class (OR: 1.62, C.I. 1.32–2.00, *p*<0.001, and OR: 1.65, C.I. 1.21–2.30, *p*<0.01, respectively), but there was no such relationship for resistance levels in Enterobacteriaceae. At an antimicrobial class level, clinical isolate resistance did not reflect the antimicrobial usage of that class in the preceding 3months (Supplementary Table S5).

Analysis of antibiotic residues reflected the high AMU within the hospital compared to the community with an average 12-fold increased residue concentration in hospital effluent (ranging between 4 and 13μl^−1^) for the five classes measured (Supplementary Figure S7). Our residue data only represents the residue levels from the whole hospital and not individual collection points and thus could not be specifically correlated with ARG abundance.

## Discussion

This study identified that hospital AMU impacts ARG abundances in hospital effluent, with implications upstream for infection control in the hospital and downstream for AMR in the environment. Overall, the distribution of bacterial genera and ARGs in our hospital wastewater samples and domestic sewage sample is similar to previously described sewage composition in European regions (Buelow et al., 2018; Hendriksen et al., 2019).

There was a significant positive relationship between inpatient department-level AMU and the abundance of antimicrobial resistance phenotype matched ARGs when all data was considered together. No relationship was found for total department AMU and ARG abundance. This supports a role of direct selection from antimicrobial usage in overall patterns of ARGs in hospital waste water, but not for indirect selection. Previous studies have found a relationship at a country level between antimicrobial residues and ARG abundance in sewage from the community (Hendriksen et al., 2019). Indeed, our data shows that the hospital antimicrobial residues for ciprofloxacin were around 9,900μg/L, well above the estimated minimum selection concentration range for *Escherichia coli* and ciprofloxacin resistance of 5–10μg/L (Kraupner et al., 2018).

The association between phenotype-matched ARGs and AMU was weak. Sewage captures resistance acquired in both the community and in the hospital, but drivers of hospital- and community-acquired resistance differ. For example, amoxicillin is used in both the community and hospitals, and resistance is widespread in the United Kingdom (60% hospital isolates resistant to amoxicillin or ampicillin in 2019; European Centre for Disease Prevention and Control, 2020), suggesting patients be more likely to arrive in hospital with carriage of amoxicillin resistance genes. The acquisition of vancomycin or carbapenem resistance, on the other hand, is associated with prior use of these antibiotics in hospital (Vasilakopoulou et al., 2020; Zhao et al., 2021), and these antibiotics are solely used parenterally in a hospital setting. Factors affecting within-hospital selection for and transmission of resistance, such as hospital antimicrobial usage, may play a stronger role in patterns of ARGs of vancomycin and carbapenems in hospital waste water than the ubiquitously used antibiotic amoxicillin. In support of this theory, we found a positive relationship between AMU and waste water ARGs for vancomycin and carbapenems, but not amoxicillin. Where a particular ward or department consumes high levels of carbapenem or vancomycin then this work demonstrates that there could be high levels of undetected fecal or urinary carriage of carbapenem and vancomycin resistance genes. This could warrant more stringent isolation of these patients, in fitting with concerns about “unsampled transmission chains” in carbapenem-resistant *Enterobacteriaceae* (Cerqueira et al., 2017). In addition, if the 70% renal excretion of unchanged meropenem (Mouton and van den Anker, 1995) selects for resistant organisms in waste water, then procedures for treatment of the bodily waste of patients on meropenem may need to be reconsidered. However, it is important to note that we cannot conclude from this study whether selection for resistance may take place within patients in the hospital or in hospital waste water and whether transfer could be plasmid mediated. Further studies that sample longitudinally from patients and hospital waste water would be required to determine routes and mechanisms of selection for resistance in hospitals and wastewater systems.

Length of stay did not impact ARG abundance in this dataset, despite prolonged duration of inpatient stay being a risk factor for carriage and infection with resistant microorganisms in previous studies (Safdar and Maki, 2002; Gupta et al., 2011; Founou et al., 2018). This appears not to support the theory of transmission of antimicrobial resistant organisms among patients and their local environment, including from the hospital water system (Kotay et al., 2017), during their inpatient stay. However, as these data were aggregated at the department-level there were few observations of length of stay, and further research with a greater sample size is needed to investigate this relationship.

Metagenomics can capture ARGs carried by a wide variety of bacterial genera, which is of benefit as the majority of ARGs are carried by non-pathogenic commensal bacteria (Sommer et al., 2009). Although, short-read sequencing cannot conclusively resolve associations between bacteria and ARGs, in our results ARGs are highly correlated with the bacteria identified at that collection point (Supplementary Figure S7). This can explain why abundance of ARGs for aminoglycosides, tetracyclines, and macrolides are higher than expected given lower proportions of phenotypic resistance in clinical isolates; the composition of bacterial genera within wastewater may have intrinsic or high levels of resistance to these antimicrobial classes. The potential for transfer of ARGs within the sewage network onto and between human pathogens has been demonstrated indicating the benefit of obtaining a universal view of ARGs (Ludden et al., 2017).

No quantitative relationship was observed between clinical isolates and ARG abundance in hospital wastewater when all data was considered together. In addition, there was no relationship between AMU in the previous 3months and resistance in clinical isolates. This may be because clinical isolates are not representative enough of carriage of resistance in the inpatient population as there is a low rate of culture positivity. However, when examined separately, there was a positive relationship between resistance in *Enterococcaceae* or *Staphylococcaceae*, but not *Enterobactericeae*, and hospital wastewater ARG abundance. The literature on these relationships is divided (Talebi et al., 2008; Tuméo et al., 2008; Yang et al., 2009; Zarfel et al., 2013; Ory et al., 2016; Hutinel et al., 2019) and future work on antimicrobial usage, specific organisms, isolate types, and ARG abundance in sewage potentially over a longer time period is required to interrogate these relationships further (Rogues et al., 2007; Mladenovic-Antic et al., 2016).

There was a higher abundance of ARGs in all hospital wastewater samples, bar one (CP4), which represents acute admissions unit, compared to Seafield. The lower abundance in Seafield could be due to dilution, and a decline in the relative abundance of AMR-gene carrying human commensal bacteria in the environment of sewerage system (Pehrsson et al., 2016), or possibly lower exposure to antimicrobial residues in community waste water. Associations between antimicrobial residues in community waste water and ARGs have been found (Hendriksen et al., 2019; Ju et al., 2019), and hospital waste water has been previously shown to have higher antimicrobial residue levels (Booth et al., 2020). Some studies comparing sewage influent in paired communities with and without a hospital have found minimal effect of a hospital on community influent (Buelow et al., 2018; Gouliouris et al., 2019). In other work, comparing resistance in hospital and community waste water has indicated some associations (Rogues et al., 2007; Pehrsson et al., 2016; Ludden et al., 2017), although, not all studies making this comparison have found evidence for a relationship (Paulshus et al., 2019).

Concern has been raised about the impact of hospital wastewater on urban influent and effluent and specific water treatments for hospital wastewater have been called for. This work highlights that physicians could consider prescribing environmentally degradable antimicrobials such as beta-lactams over antimicrobials, which have persistent residues across environmental niches e.g., tetracycline to minimize the impact of antimicrobials on the environmental resistome (Wellington et al., 2013). The ultimate effect of environmental ARGs on human disease is an ongoing important research question (Bürgmann et al., 2018).

The use of metagenomics is a key strength of this study, allowing quantification of resistance genes to a wide range of antibiotics and retrospective investigation if new resistance genes emerge. The 24-h composite samplers provide a representative sample of the hospital (Chau et al., 2020), although hospital staff, outpatients, and visitors will have also contributed to the effluent. In addition, some patients will have moved around the hospital during the sampling period. Although, this study is limited to one hospital site at one time point the variation in antimicrobial use and inpatient characteristics in each department has allowed us to treat them as discrete treatment centers and draw conclusions about factors affecting ARG abundance.

There is little doubt that hospital resistant pathogens can be abundant in wastewater systems (Maheshwari et al., 2016; Ludden et al., 2017; Gouliouris et al., 2019). However, using metagenomic sequencing, we show that resistance in hospital wastewater may quantitatively reflect clinical isolate resistance for some bacterial species (*Enterococcaceae* and *Staphylococcaceae*), although not all. As a surveillance tool this novel technique can represent the burden of AMR carriage in hospital inpatients and hospital pipes for specific resistance genes relating to important parenteral antimicrobials such as carbapenems and vancomycin. It may also aid in identification of emerging patterns of ARG abundance and novel ARGs, and how they may relate to changing patterns of transmission, infection control policies, and antimicrobial usage. Further longitudinal work evaluating the wastewater from multiple hospital sites is needed to establish AMU/ARG relationships, optimal collection points and sampling methods to be able to develop this as a surveillance technique.

In conclusion, we show in a multi-departmental study that the relationships between ARG abundance in hospital wastewater and hospital AMU or clinical resistance levels may vary by antimicrobial type and bacterial species. Our study emphasizes in a novel way the ARG burden from the high antimicrobial consuming and high resistance carriage environment of the hospital and that promoting active antimicrobial stewardship, particularly of key parenteral antimicrobials such as carbapenems and vancomycin, would impact the burden of environmental AMR. Hospital wastewater is an important source of AMR into the environment; this should be considered in environmental policy to reduce the flow of AMR between different environmental reservoirs.

## Data Availability Statement

The datasets presented in this study can be found in online repositories. The names of the repository/repositories and accession number(s) can be found at: https://www.ebi.ac.uk/ena, PRJEB34410.

## Ethics Statement

This study was conducted following approval from NHS Lothian Research and Development Committee under the sponsorship of University of Edinburgh. There was no direct patient contact and therefore the study did not require ethical board approval.

## Author Contributions

MP conceived the project and developed it with input from BB, HL, FA, and MW. MP facilitated sampling and DNA extraction with AW. PK, CP, and BM provided clinical and pharmaceutical databases and input. AH performed antibiotic residue analysis. HL, BB, LM, BW, PM, and MP performed bio-informatics analyses with input from FA and MW. MP and HL drafted the manuscript with input from BB and review and comments from all authors. All authors contributed to the article and approved the submitted version.

## Funding

This work was funded by Academy of Medical Sciences (SGL016_1086 to MP), an Institutional Strategic Support Fund from University of Edinburgh (J22738 to MP), and the Novo Nordisk Foundation (NNF16OC0021856: Global Surveillance of Antimicrobial Resistance to HL, BB, LM, MW, PM, and FA).

## Conflict of Interest

The authors declare that the research was conducted in the absence of any commercial or financial relationships that could be construed as a potential conflict of interest.

## Publisher’s Note

All claims expressed in this article are solely those of the authors and do not necessarily represent those of their affiliated organizations, or those of the publisher, the editors and the reviewers. Any product that may be evaluated in this article, or claim that may be made by its manufacturer, is not guaranteed or endorsed by the publisher.

## Footnotes

^1^ https://www.ebi.ac.uk/ena/data/view/PRJEB34410
