# Supplementary material for: Secrets of the Hospital Underbelly: Patterns of Abundance of Antimicrobial Resistance Genes in Hospital Wastewater Vary by Specific Antimicrobial and Bacterial Family
Source: Front Microbiol. 2021 Sep 10;12:703560. doi: 10.3389/fmicb.2021.703560 (PMC8461093; doi:10.3389/fmicb.2021.703560)
Supplement: Supplementary file 1 [file Data_Sheet_1.pdf]

Supplementary tables

Name	All read pairs	Non-human read pairs	% of total reads	Bacterial read pairs	% of non-human reads	Viral read pairs	% of non-human reads	Unclassified read pairs	% of non-human reads	AMR gene read pairs	% of non-human reads
CP1	38869360	37007788	95	20284420	52	36350	0.09	19414692	50	57318	0.15
CP3	38950823	37111066	95	23077786	59	7052	0.02	15802719	41	153806	0.39
CP4	38854316	38656944	99	27184108	70	2945	0.01	12865522	33	27632	0.07
CP5	39062827	38294655	98	27458178	70	8784	0.02	12632702	32	123020	0.31
CP6	38503994	28249480	73	21423749	56	30321	0.08	8045607	21	73101	0.19
CP7	39176215	37801241	96	21622738	55	5525	0.01	18844242	48	102444	0.26
CP8	35716605	35093020	98	22709754	64	6201	0.02	14292670	40	132598	0.37
Seafield	38298451	38143462	100	28016716	73	3594	0.01	11196189	29	40977	0.11

Table S1. Total read pairs, read pairs assigned to major taxonomic groups and antimicrobial resistance gene read pairs.

Table S2. gDNA extraction concentration in each sample.

Name	Concentration (ng/ul)
CP1	13.9
CP2	134
CP3	18.3
CP4	38.8
CP5	25
CP6	5.28
CP7	40.6
CP8	9.78
Full	38.4
Seafield	82.6

Table S3 Bacterial genera at each collection point
Please see separate excel file

Table S4 AMR genes at each collection point (FPKM)
Please see separate excel file

	Clinical Isolate Model		
Coefficients	*Odds Ratios*	*Conf. Int. (95%)*	*P-Value*
Intercept	0.02	0.00 – 0.11	**<0.001**
AMU (log, urine/faecal samples)	0.92	0.64 – 1.31	0.629
AMU (log, non-urine/faecal samples)	1.27	0.85 – 1.89	0.245
Isolate Type = Urine	8.05	2.69 – 24.15	**<0.001**
**Random Effects**			
σ^2	3.29		
$\tau_{00\ \mathrm{Observation}}$	1.82		
$\tau_{00\ \mathrm{Organism}}$	3.40		
$\tau_{00\ \mathrm{Antibiotic\ Class}}$	4.04		
$\tau_{00\ \mathrm{Site}}$	0.01		
$\mathrm{ICC}_{\mathrm{Observation}}$	0.14		
$\mathrm{ICC}_{\mathrm{Organism}}$	0.27		
$\mathrm{ICC}_{\mathrm{Antibiotic\ Class}}$	0.32		
$\mathrm{ICC}_{\mathrm{Site}}$	0.00		
Observations	1124		
Marginal R^2 / Conditional R^2	0.034 / 0.607		

Table S5. Output from model examining the relationship between resistance in clinical isolates and antimicrobial usage (AMU)

Data subset	Number of gene cluster groups	Number of observations	Fixed effects			Random effects		
			**Variable**	**IRR (CI)**	**P value**	$\tau_{Observation}$	$\tau_{Cluster}$	τ_{Site}
All data	106	584	Log of total DDDs<br>Log of phenotype-matched DDDs<br>Average length of stay	0.87 (0.43 - 1.73)<br>1.11, (1.06 - 1.16)<br>1.06 (0.97 - 1.17)	0.64<br><0.001<br>0.11	1.21	2.61	0.13
Carbapenems	12	56	Log of phenotype-matched DDDs	1.91 (1.01 – 3.72)	0.07†	2.01	2.51	0.13
Vancomycin	3	11	Log of phenotype-matched DDDs	10.25 (2.32 – 49.10)	<0.001†	-	0.46	1.88
Amoxicillin	42	172	Log of phenotype-matched DDDs	0.68 (0.29 – 1.55)	0.84†	1.51	2.42	0.12

Table S5. Data summary and output from models examining the relationship between antimicrobial usage (AMU) and antimicrobial resistance gene abundance in waste water. Each row represents a separate model. Abbreviations: DDDs=daily defined doses, CI=confidence interval. † P values Bonferroni corrected

Data subset	Number of bacterial species	Number of observations	Fixed effects			Random effects		
			Variable	Odds Ratios (CI)	P value	$\tau_{Observation}$	τ_{Class}	$\tau_{Organism}$
All data	28	2595	Urine or faecal samples: Log sewage FPKM<br><br>Other sample sources: log of sewage FPKM	1.13 (0.90 – 1.42)<br><br>0.88 (0.69 – 1.12)	0.281<br><br>0.293	1.84	3.87	3.54
Enterococcaceae	3	201	Log sewage FPKM	1.62 (1.33 – 2.00)	<0.001†	-	-	-
Staphylococcaceae	5	224	Log sewage FPKM	1.65 (1.21 – 2.30)	0.006 †	-	-	-
Enterobacteriaceae	9	395	Log sewage FPKM	0.92 (0.79 – 1.01)	0.92 †	-	-	-

Table S7. Summary of data and output of models examining the relationship between clinical isolates and ARG abundance in waste water. ORs are for the log FPKM of ARGs matched to the antimicrobial class of the resistance test of the isolate. The three bacterial families were modelled separately. † P values Bonferroni corrected

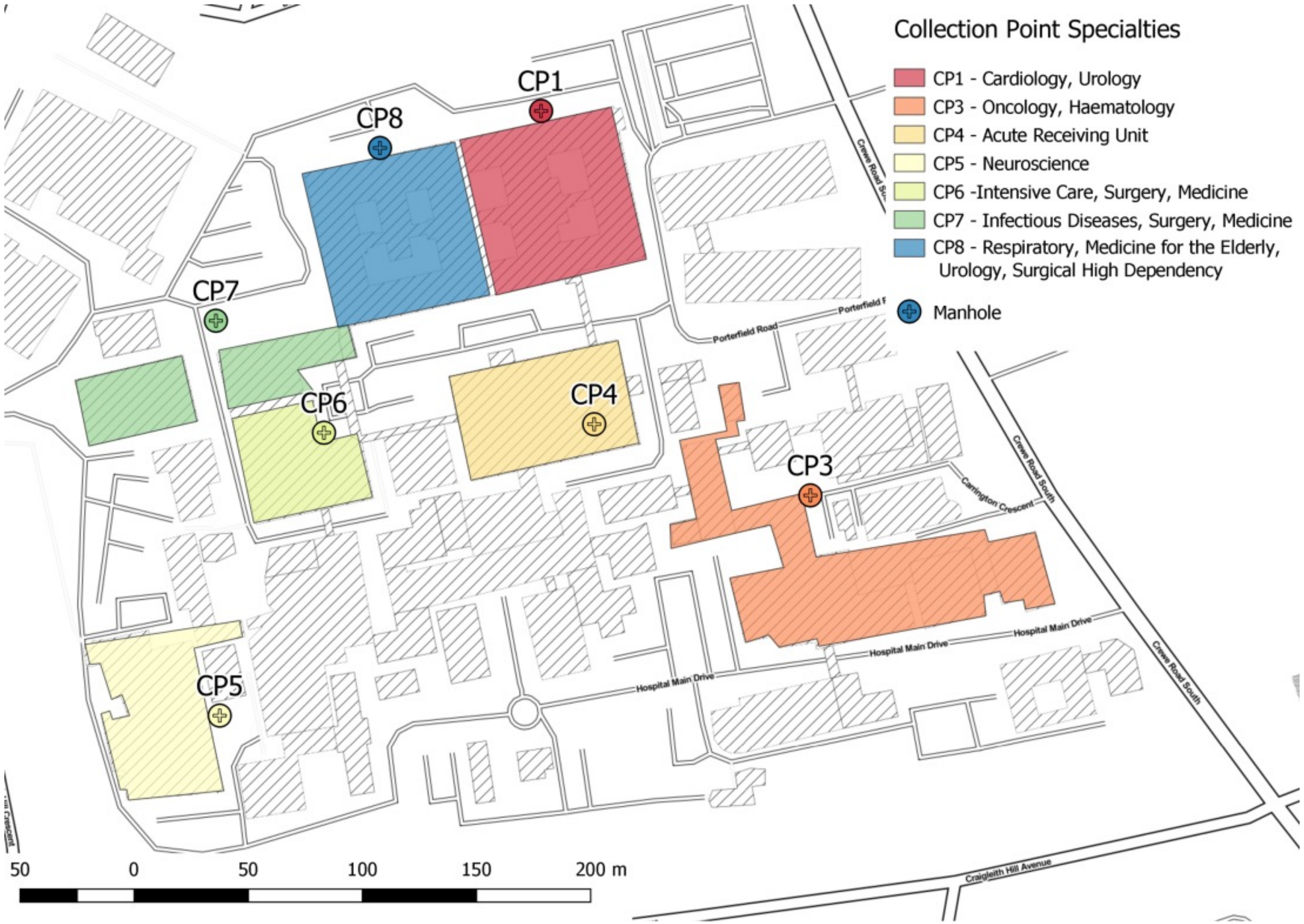

Supplementary Figure 1 Schemata of location of analysed wastewater Collection Points (CP) on tertiary hospital site
Hospital wastewater was sampled from indicated locations, which are the main clinical areas of the hospital site, over a 24-hour period using composite samplers. Shaded areas represent the drainage of the sampled manholes. CP4 was a manhole located underneath the hospital in a plant room.

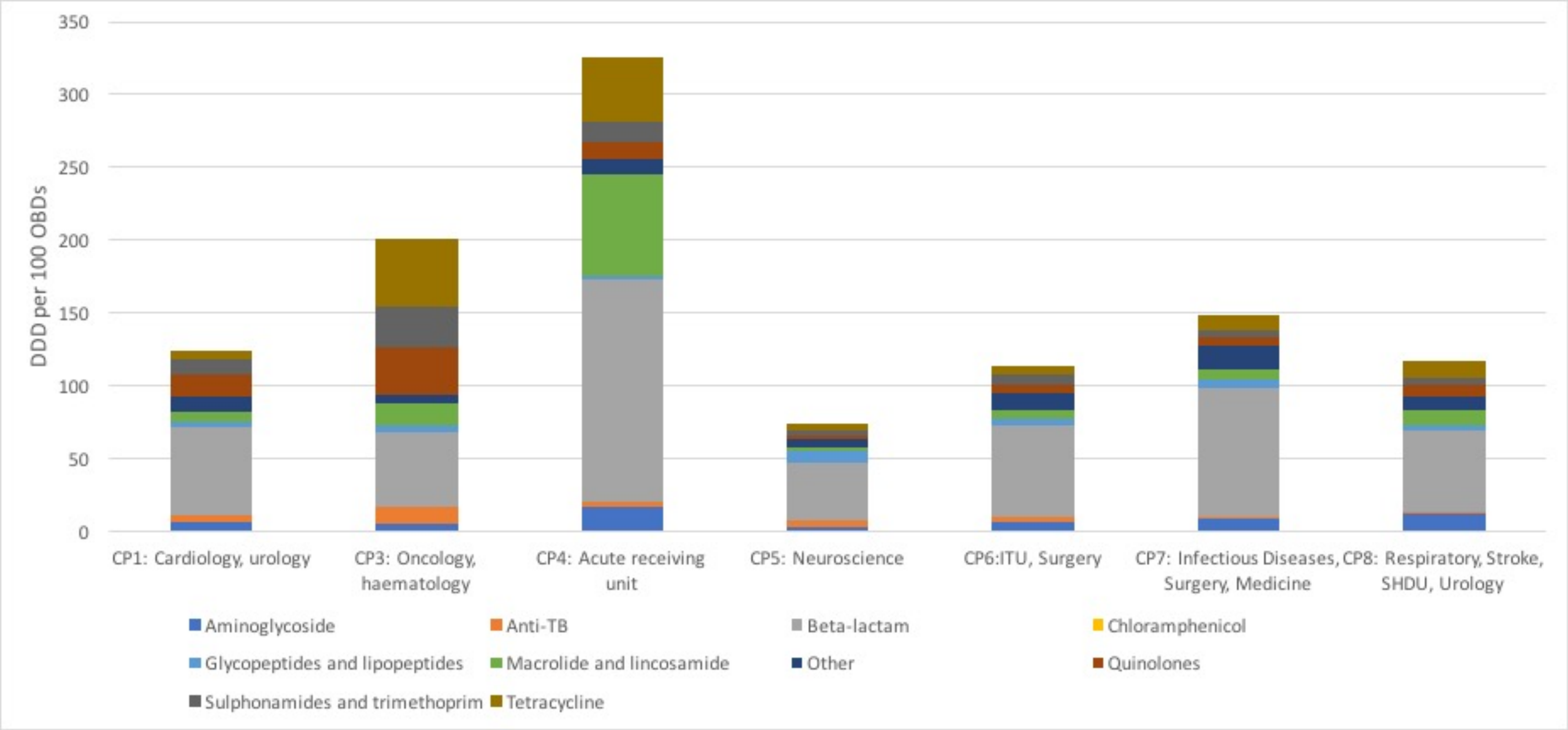

Supplementary Figure 2 Antimicrobial consumption by class over different hospital departments

Pharmacy issues to the wards within the collection points were collated over a three -month period and defined daily dose (DDD) per 100 occupied bed days (OBD) calculated to represent the antimicrobial consumption at each collection point.

DDD= defined daily dose, OBD = occupied bed day, ITU=intensive care unit, SHDU = surgical high dependency unit.

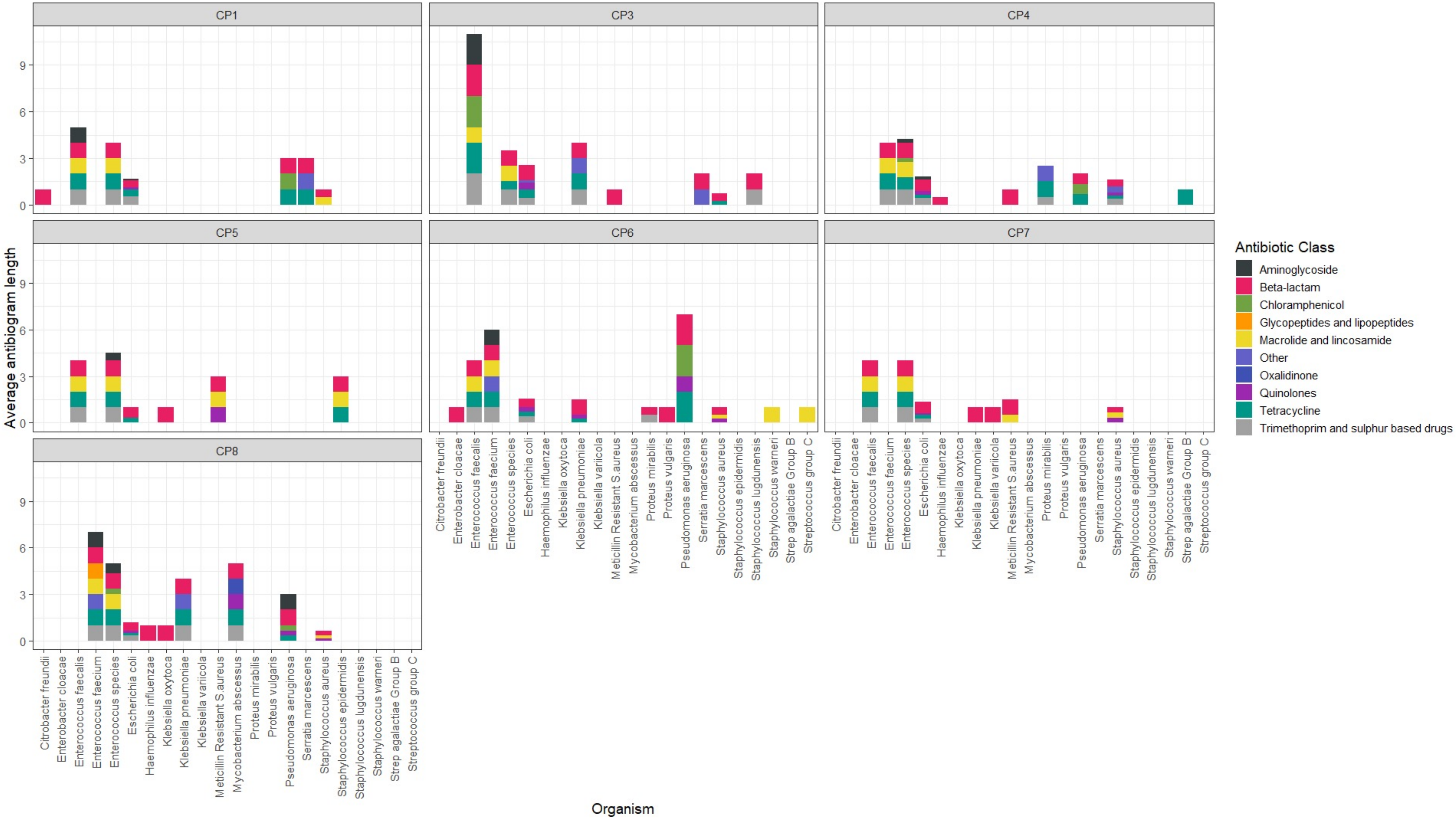

Supplementary Figure 3 Phenotypic resistance in clinical isolates by antimicrobial class represented by average antibiogram length per organism at each collection point

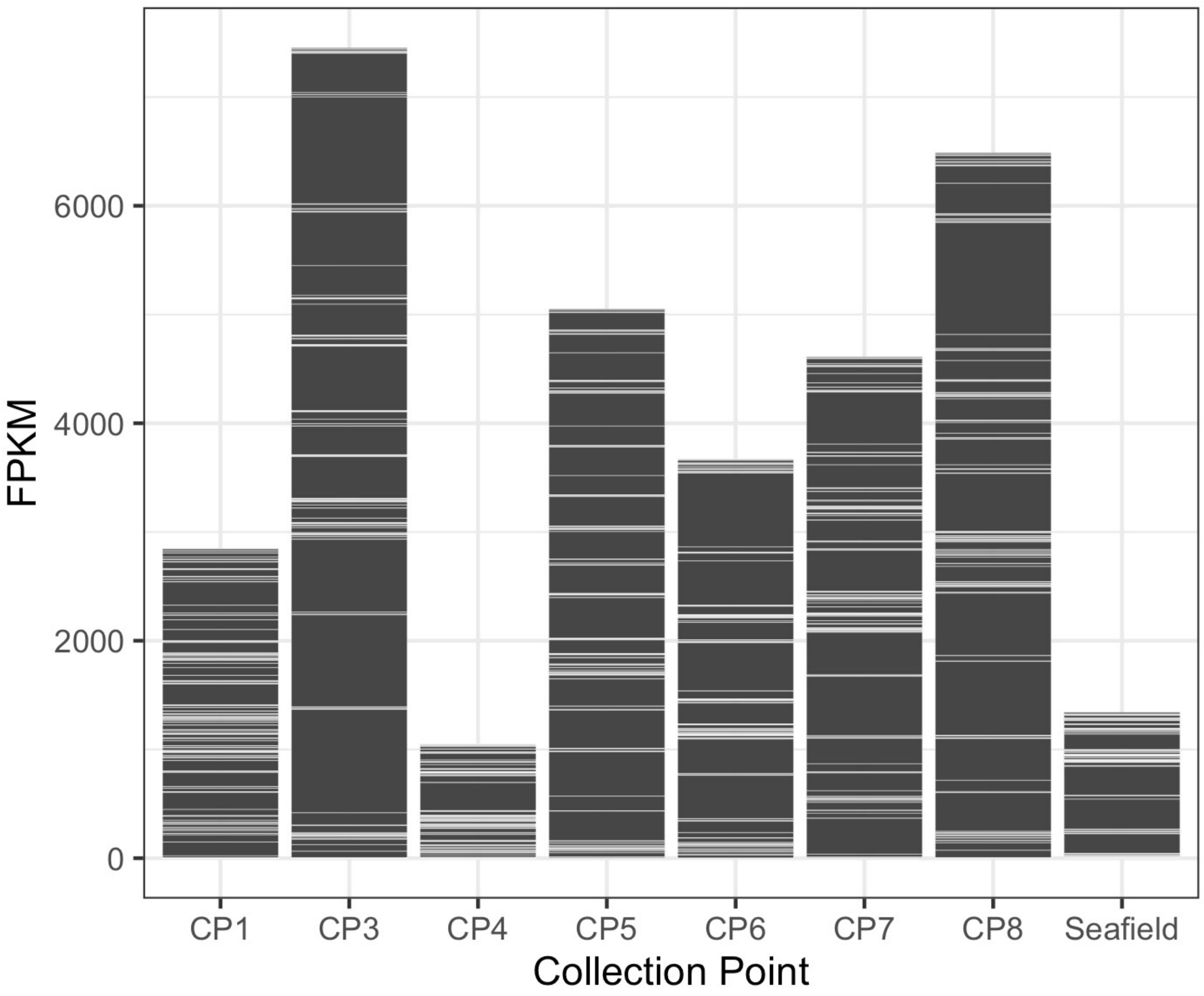

Supplementary Figure 4 Total AMR gene abundance in FPKM

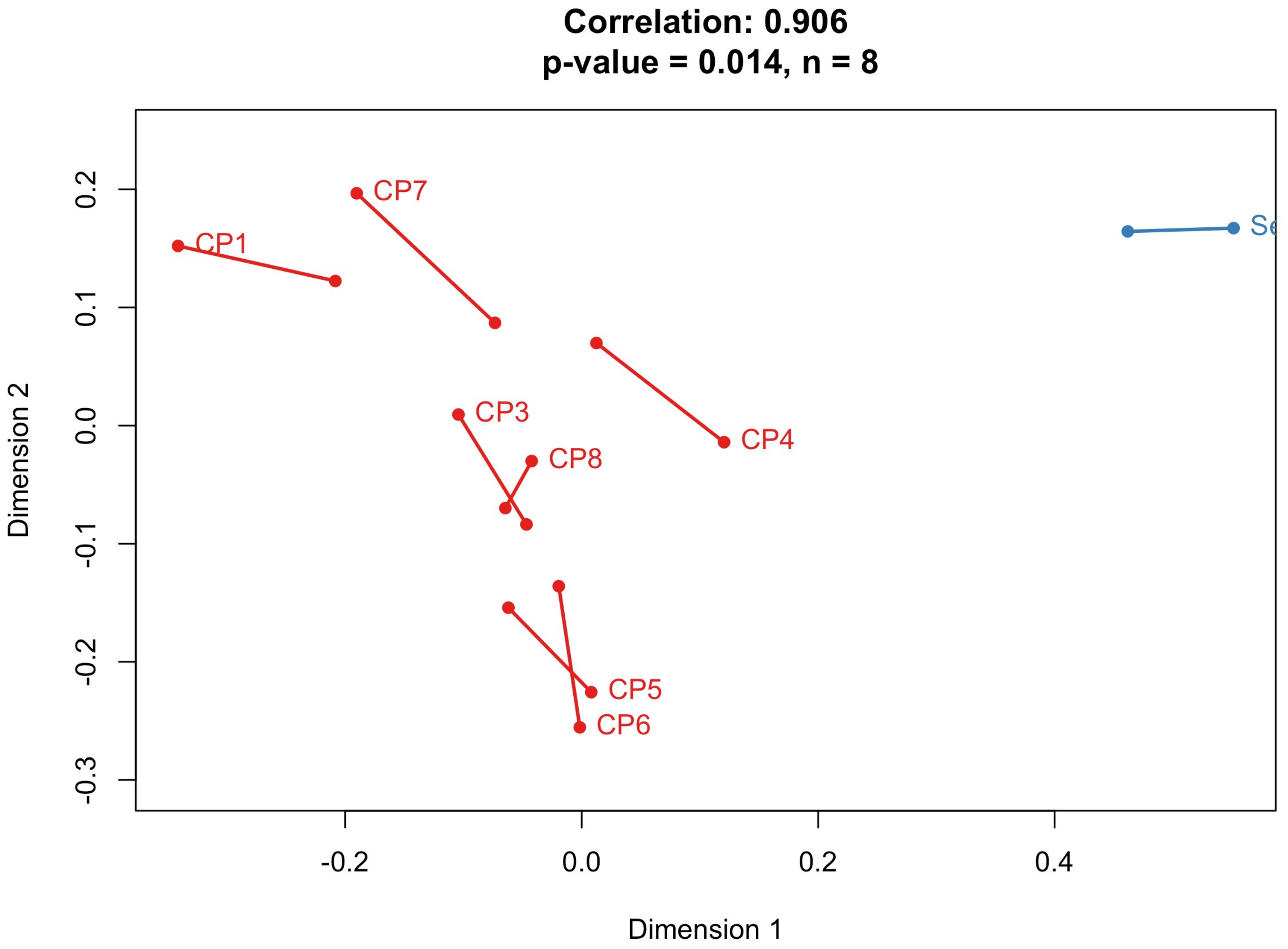

Supplementary Figure 5 Procrustes analysis on ordinations of AMR genes and bacterial composition

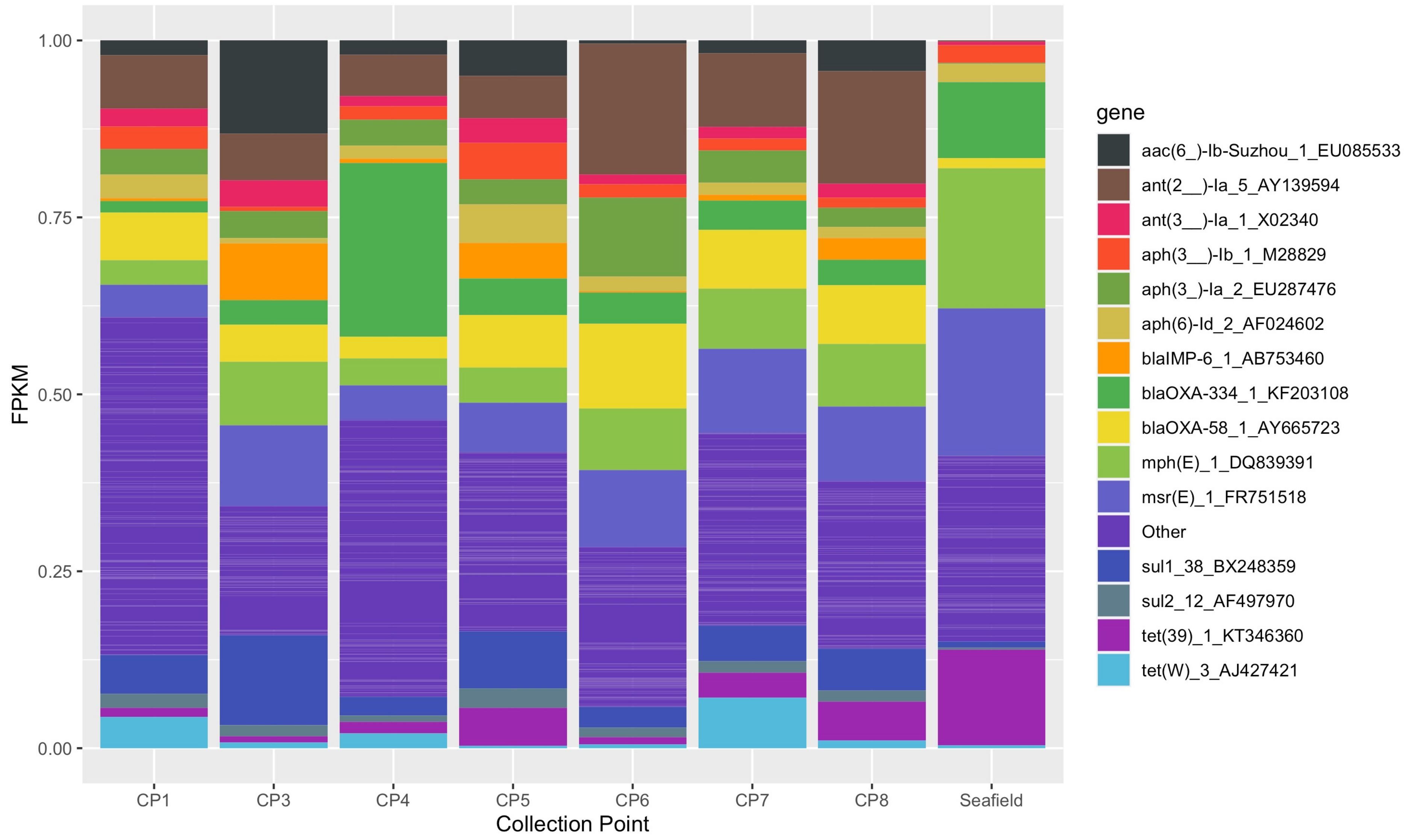

Supplementary Figure 6 Fifteen most common AMR genes relative to total abundance within samples

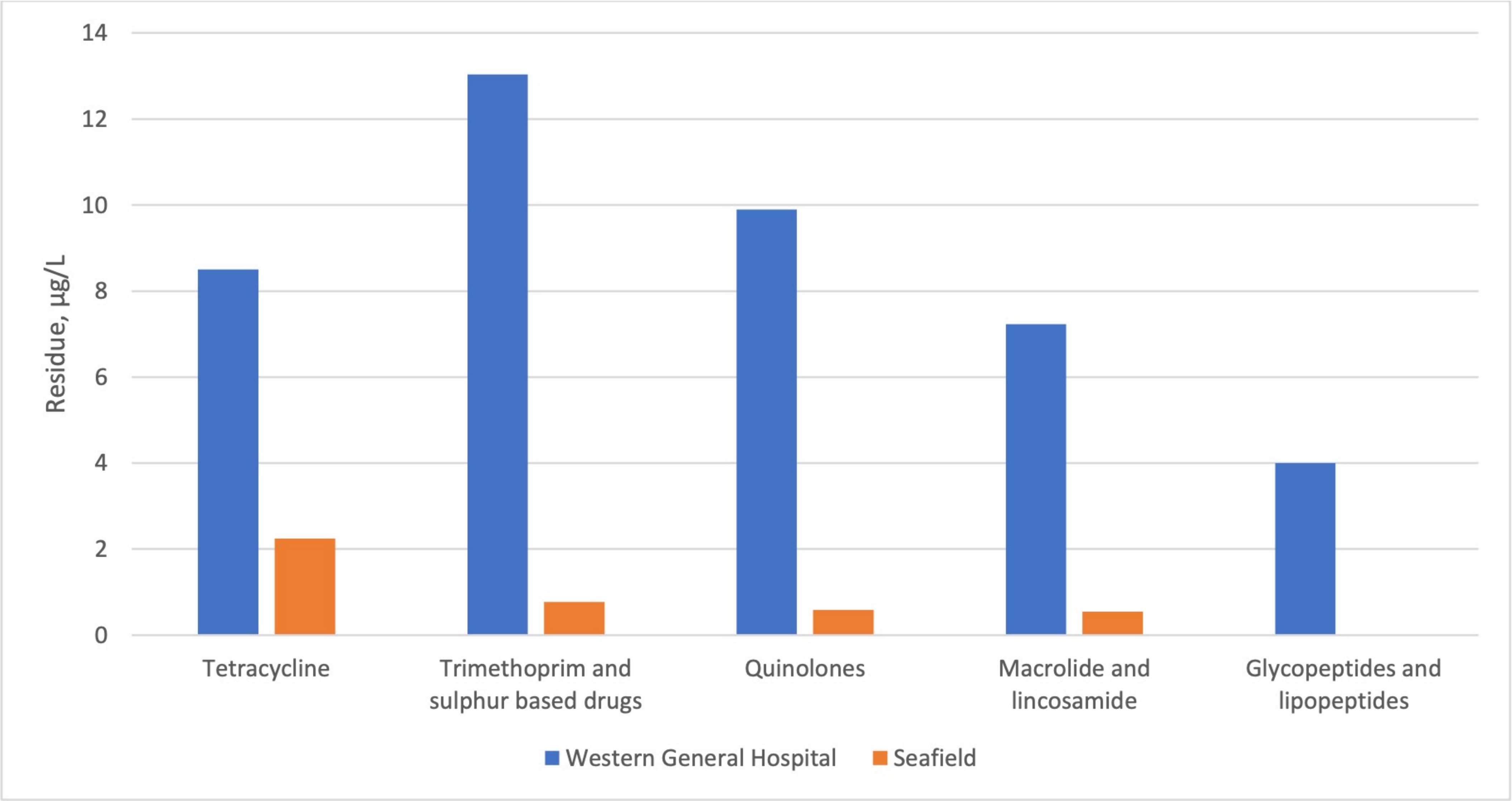

Supplementary Figure 7 Antibiotic residues from composite hospital wastewater sample and urban sewage as measured by LC-MS/MS

Table S3

Bacterial genus	CP1	CP3	CP4	CP5	CP6	CP7	CP8	Seafield
Acinetobacter	1604595	8858221	14545264	9253608	5301071	6084958	7617102	9367455
Moraxella	5012	8479	22845	8956	4858	8743	6683	751049
Psychrobacter	1252	5393	13810	2168	808	2426	2179	247366
Pseudomonas	1337571	2075434	3603155	1362168	7330230	1387450	2464891	8155080
Azotobacter	3588	4395	3655	5564	3015	2741	3052	4728
Oblitimonas	251	661	1513	339	133	257	484	1117
Klebsiella	854067	61633	42472	220763	62338	210958	324999	55315
Escherichia	626094	64854	49225	126536	1116220	210557	327398	26513
Citrobacter	27522	14909	40420	54589	13373	38541	58932	49004
Enterobacter	25852	13976	31045	30815	26297	53850	35869	95756
Salmonella	5921	2945	3674	3677	2605	4764	3806	8412
Candidatus Blochmannia	313	156	174	128	63	152	312	239
Candidatus Hamiltonella	158	136	214	109	86	149	206	843
Candidatus Doolittlea	90	27	13	10	9	26	25	35
Candidatus Tachikawaea	47	24	13	7	10	25	29	28
Candidatus Hoaglandella	39	45	19	18	12	26	35	36
Candidatus Purcelliella	33	27	23	32	4	22	15	56
Candidatus Annandia	32	15	1	5	1	12	6	15
Candidatus Gullanella	29	19	35	6	10	42	11	34
Candidatus Mikella	17	15	38	13	4	10	17	14
Raoultella	3701	7294	3595	7532	5081	11160	15986	21321
Cronobacter	2664	1892	1710	1864	1033	2194	2214	5896
Shigella	2149	218	131	299	1123	359	1077	325
Kosakonia	1948	1211	1999	1278	925	1741	1294	5056
Pluralibacter	1826	1032	1005	1264	632	1387	1128	2605
Lelliottia	1314	893	1444	2044	727	1416	1035	65515
Cedecea	1186	941	1520	912	538	1124	885	5535
Metakosakonia	793	115	281	427	223	660	447	206
Leclercia	694	736	2595	555	437	604	607	5465
Gibbsiella	467	313	590	513	240	394	363	818
Kluyvera	411	175	625	167	606	1107	266	36089
Atlantibacter	364	672	168	267	169	293	274	1206
Shimwellia	343	196	163	189	110	228	213	710
Buttiauxella	224	377	1407	353	132	249	224	41672
Limnobaculum	185	128	915	67	58	177	185	357
Candidatus Ishikawaella	69	28	16	20	4	32	25	113
Candidatus Riesia	49	51	14	18	17	80	48	43
Candidatus Moranella	47	31	5	22	11	19	22	17

**Serratia**	12923	18865	8705	18952	5314	10265	9238	45081
**Yersinia**	5281	2627	7156	12727	2233	2806	2213	105850
**Rahnella**	1273	1174	938	1099	881	1033	1196	75871
**Chania**	278	215	171	253	119	286	231	629
**Candidatus Fukatsuia**	117	169	109	399	49	141	137	175
**Proteus**	7858	1200	3296	1661	11821	2600	1410	2789
**Morganella**	4465	3104	1067	2398	3100	2455	2519	1460
**Providencia**	927	1559	5254	863	500	1305	1399	3933
**Xenorhabdus**	717	728	2760	391	356	675	594	1978
**Photorhabdus**	360	403	1838	286	131	345	367	1133
**Arsenophonus**	124	165	167	27	45	81	65	230
**Dickeya**	4408	3710	3294	4274	1883	3607	3502	7807
**Pectobacterium**	1875	1692	2195	1728	742	1852	1603	6714
**Brenneria**	898	747	1141	668	341	832	677	1167
**Sodalis**	855	710	526	607	361	713	487	762
**Lonsdalea**	227	194	124	266	138	179	186	311
**Pantoea**	3335	2832	4232	2967	1553	3335	2551	8308
**Erwinia**	1604	1419	2176	1485	777	1395	1401	5097
**Buchnera**	459	416	135	202	120	280	273	420
**Mixta**	365	306	233	322	173	272	237	422
**Tatumella**	334	322	199	211	132	325	227	638
**Wigglesworthia**	51	44	26	22	11	39	36	137
**Plesiomonas**	2831	542	273	726	172	1557	761	1350
**Phytobacter**	1833	953	2854	1000	2232	3887	1244	648
**Edwardsiella**	1705	1265	1197	1227	672	1609	1110	1526
**Hafnia**	451	625	1257	2323	762	1064	1375	6343
**Obesumbacterium**	117	80	53	111	865	334	124	1872
**Leminorella**	337	195	171	164	94	338	150	318
**Pragia**	295	347	723	145	160	401	252	1010
**Stenotrophomonas**	129607	272935	232378	137529	46509	37691	55058	58882
**Xanthomonas**	66411	49720	39052	65787	20620	22048	33576	24331
**Lysobacter**	65277	36462	24246	49947	12289	13463	25705	18990
**Pseudoxanthomonas**	48212	23750	15725	43240	14476	9043	14187	8727
**Luteimonas**	36083	17673	12756	25889	5827	6881	10198	8315
**Xylella**	1063	716	504	949	277	528	634	724
**Dyella**	9855	7904	3067	6179	2361	3569	5141	2283
**Dokdonella**	8473	6873	1991	4290	1666	2163	5308	1386
**Rhodanobacter**	5853	4424	1936	4384	1213	2151	3149	1353
**Luteibacter**	2311	1914	701	1413	642	1041	1278	581
**Ahniella**	1070	837	494	751	355	450	631	464

**Frateuria**	918	776	822	743	303	503	616	455
**Haemophilus**	49097	12316	3163	3159	3787	17155	10240	5388
**Aggregatibacter**	973	744	525	302	229	684	615	1069
**Actinobacillus**	892	716	982	416	220	777	596	1438
**Pasteurella**	623	1649	1553	565	220	555	1077	1769
**Mannheimia**	522	461	1258	373	156	471	404	1070
**Glaesserella**	363	287	570	120	123	282	285	739
**Avibacterium**	197	171	1285	103	69	151	332	445
**Histophilus**	191	141	80	88	48	143	219	315
**Bibersteinia**	167	184	344	53	34	176	124	314
**Basfia**	118	103	538	57	33	150	158	224
**Gallibacterium**	116	172	152	70	58	153	129	324
**Aeromonas**	46560	29421	45972	52462	52976	189850	169742	613987
**Tolumonas**	1346	1079	417	641	290	1022	832	10857
**Zobellella**	1154	853	703	921	512	828	706	691
**Oceanimonas**	631	501	519	441	271	512	407	592
**Oceanisphaera**	331	311	1958	477	128	411	253	984
**Thioalkalivibrio**	4654	3009	2298	3284	1563	2496	2569	1652
**Acidihalobacter**	2325	1818	1420	2218	795	1297	1469	1305
**Ectothiorhodospira**	1105	877	645	853	354	829	705	635
**Halorhodospira**	911	582	526	612	341	665	470	383
**Alkalilimnicola**	819	604	536	725	303	484	485	311
**Spiribacter**	466	338	276	442	186	421	329	286
**Thiodictyon**	1590	1288	805	1414	598	964	829	749
**Marichromatium**	1131	749	581	944	464	644	590	377
**Allochromatium**	1047	683	520	720	366	516	468	364
**Thioflavicoccus**	984	749	520	632	349	609	556	414
**Thiocystis**	875	792	520	745	383	547	550	480
**Nitrosococcus**	423	378	283	282	152	381	278	476
**Rheinheimera**	190	322	463	1077	146	301	174	543
**Candidatus Nitrosoglobus**	156	74	391	135	28	88	92	256
**Sulfurivermis**	1956	1047	746	1748	603	1020	993	651
**Halothiobacillus**	846	765	503	800	422	724	632	672
**Wenzhouxiangella**	800	617	359	742	295	478	479	294
**Sulfuriflexus**	334	274	261	193	101	287	292	221
**Granulosicoccus**	319	221	230	265	124	282	229	293
**Woeseia**	313	254	150	191	114	233	239	184
**Halomonas**	9330	8146	8044	8597	3970	7061	7016	7430
**Halotalea**	955	820	575	997	458	588	591	512
**Salinicola**	833	684	505	809	371	630	599	406

**Kushneria**	804	547	423	591	311	649	502	492
**Chromohalobacter**	652	516	406	653	292	369	372	357
**Cobetia**	436	378	235	381	176	318	283	328
**Zymobacter**	235	204	419	192	79	228	139	153
**Candidatus Carsonella**	22	31	15	14	14	19	17	28
**Candidatus Evansia**	22	14	18	6	4	21	20	15
**Candidatus Portiera**	8	9	15	14	2	11	8	25
**Alcanivorax**	3246	2586	2890	3040	1645	2363	2488	2551
**Ketobacter**	247	247	166	171	102	274	153	309
**Marinobacterium**	597	542	374	716	343	503	500	557
**Marinomonas**	562	609	1238	453	213	656	732	1628
**Thalassolituus**	460	541	1805	570	138	347	510	1203
**Bacterioplanes**	181	163	137	112	68	180	109	413
**Oleispira**	131	386	322	998	52	280	192	963
**Saccharospirillum**	377	291	230	305	185	357	298	307
**Gynuella**	266	266	822	187	103	263	171	404
**Reinekea**	187	177	123	201	91	157	155	223
**Hahella**	650	606	726	598	328	605	486	708
**Kangiella**	330	360	1822	240	123	262	360	981
**Endozoicomonas**	223	263	389	132	61	278	150	347
**Oleiphilus**	146	201	127	96	55	162	86	263
**Marinobacter**	4332	3900	3863	4148	1849	3791	3502	4416
**Alteromonas**	1407	1627	3882	1217	469	1361	1341	3069
**Glaciecola**	741	852	849	911	279	585	990	744
**Salinimonas**	322	263	174	211	101	296	187	542
**Lacimicrobium**	240	210	440	158	126	273	159	327
**Catenovulum**	155	204	872	137	57	152	127	309
**Paraglaciecola**	122	153	162	103	39	138	128	428
**Agarivorans**	117	76	139	95	41	84	133	246
**Shewanella**	5072	5360	11262	6674	3964	4975	4756	817172
**Pseudoalteromonas**	2321	4381	8330	2527	980	2795	3160	8340
**Colwellia**	739	1443	3822	647	312	755	1376	2537
**Thalassotalea**	134	117	67	58	21	111	71	487
**Litorilituus**	63	114	661	51	30	79	154	351
**Idiomarina**	437	464	845	277	165	386	484	954
**Ferrimonas**	488	358	231	402	193	331	309	322
**Moritella**	268	299	2237	309	111	264	253	883
**Psychromonas**	255	328	715	340	78	218	212	676
**Vibrio**	7078	7250	16650	6862	3018	7364	6597	23410
**Photobacterium**	652	702	1337	431	518	748	521	1226

**Aliivibrio**	482	433	1843	435	325	505	615	1180
**Grimontia**	190	146	416	91	58	212	113	208
**Paraphotobacterium**	92	47	29	41	33	120	85	137
**Enterovibrio**	45	27	32	35	13	35	27	87
**Methylomonas**	1959	2020	1131	1705	921	1579	1406	2093
**Methylococcus**	1455	727	529	979	540	748	743	367
**Methylomicrobium**	1282	944	701	1175	440	884	778	913
**Methylocaldum**	908	765	452	853	395	547	532	579
**Methylovulum**	374	3552	614	1831	554	1064	1194	2718
**Thiohalobacter**	1020	866	574	832	365	688	667	516
**Sedimenticola**	496	298	246	327	155	367	268	350
**Candidatus Ruthia**	57	47	79	16	19	52	34	63
**Thiolapillus**	305	201	182	213	90	286	193	207
**Gallaecimonas**	239	243	164	174	64	271	181	354
**Candidatus Thioglobus**	235	221	607	79	64	131	329	434
**Pseudohongiella**	206	173	155	101	75	189	110	271
**Candidatus Nardonella**	42	48	11	17	5	48	29	42
**Cellvibrio**	1255	3710	1870	1342	549	919	982	1425
**Simiduia**	334	344	314	333	135	290	243	470
**Teredinibacter**	187	172	110	129	54	174	119	286
**Saccharophagus**	128	179	346	102	38	84	71	226
**Agarilytica**	123	122	595	59	51	107	185	231
**Microbulbifer**	1203	1007	1824	871	434	1038	702	916
**Halioglobus**	745	539	372	569	263	554	413	554
**Congregibacter**	290	290	277	226	155	348	239	258
**Zhongshania**	206	235	165	567	106	169	146	466
**Oceanicoccus**	154	156	447	84	52	124	103	210
**Spongiibacter**	113	155	240	111	44	83	106	329
**Solimonas**	1973	1584	1133	1683	878	1071	1302	757
**Steroidobacter**	1323	1151	865	1338	366	615	763	413
**Methylophaga**	375	659	1200	435	140	358	327	1221
**Thiomicrospira**	350	317	1141	210	109	309	581	782
**Thiomicrorhabdus**	242	285	274	126	59	276	173	443
**Cycloclasticus**	153	171	125	190	56	213	165	366
**Hydrogenovibrio**	143	170	185	166	55	159	164	281
**Piscirickettsia**	111	188	851	59	30	85	83	232
**Francisella**	1083	921	1319	741	409	1041	1162	2049
**Allofrancisella**	83	38	10	17	36	44	29	56
**Thioploca**	144	152	94	51	54	107	91	262
**Beggiatoa**	141	251	145	83	49	100	178	794

**Legionella**	2004	2358	3480	1430	1037	1903	1674	2795
**Tatlockia**	126	142	54	150	263	124	108	135
**Fluoribacter**	81	51	33	40	24	91	60	119
**Coxiella**	187	226	264	158	71	170	132	258
**Rickettsiella**	44	73	562	49	33	59	45	124
**Sulfurifustis**	1170	871	615	838	431	634	626	324
**Sulfuricaulis**	840	647	367	677	351	525	448	430
**Acidiferrobacter**	444	336	178	274	176	276	250	180
**Immundisolibacter**	1537	1131	922	1373	558	918	947	604
**Salinisphaera**	722	726	414	627	373	455	525	301
**Cardiobacterium**	511	363	337	375	200	429	317	369
**Dichelobacter**	151	153	554	56	56	135	103	395
**Gilliamella**	166	230	107	84	55	207	222	433
**Frischella**	98	69	46	34	38	64	109	150
**Acidovorax**	781425	1369344	758282	1990867	326274	653834	1014081	1335766
**Comamonas**	369001	91937	393272	153795	44170	82376	282577	364347
**Delftia**	125105	56636	63662	254659	56084	89227	172752	64968
**Variovorax**	101855	139067	60486	191064	63874	53079	135785	66088
**Ottowia**	89737	39380	28935	427908	18443	40606	82365	43083
**Alicycliphilus**	72772	74776	108903	96916	25107	25766	50996	31937
**Hydrogenophaga**	47746	51325	46694	85918	24161	28232	62556	42989
**Melaminivora**	46265	47921	62281	73121	18549	21990	46336	35583
**Rhodoferax**	35159	50962	28713	96087	25980	25708	70217	51570
**Polaromonas**	28206	39704	23669	70164	19613	19650	52955	45421
**Ramlibacter**	22453	25050	17008	36844	12243	11749	28907	14923
**Simplicispira**	14768	17877	14635	32410	6927	10731	21101	27093
**Verminephrobacter**	13542	13904	12296	26675	5710	9183	19784	16459
**Serpentinomonas**	11585	9354	9652	17361	5451	6499	11469	11033
**Limnohabitans**	9520	11031	8650	23975	5083	7533	16447	15870
**Curvibacter**	6585	8347	6429	16253	3667	4901	10954	10385
**Diaphorobacter**	5666	5806	13122	14256	2453	3307	5872	4802
**Candidatus Symbiobacter**	1665	1232	1191	2100	530	1082	1602	1832
**Burkholderia**	86269	68978	41345	84613	42003	53193	60221	34190
**Cupriavidus**	74739	62019	43885	64067	59721	72060	60302	29694
**Ralstonia**	27680	22537	18240	31573	11900	14772	24697	14416
**Paraburkholderia**	18625	18710	11564	21047	11389	12114	15724	11813
**Pandoraea**	17646	17268	10561	17358	7997	9628	14802	10213
**Lautropia**	1893	1507	1153	2128	2765	1385	2437	1041
**Polynucleobacter**	1856	2107	3806	1826	644	1708	1931	4809
**Hydromonas**	382	236	240	351	85	208	294	41656

**Mycoavidus**	182	101	131	197	44	102	177	279
**Methylibium**	12238	9457	6402	10953	6521	4836	8626	3811
**Thiomonas**	10829	8475	6966	14336	4045	7592	9513	10374
**Rubrivivax**	9960	7764	6608	8618	6275	4087	6543	2680
**Rhizobacter**	8551	7061	4261	8484	4894	3733	6753	3338
**Leptothrix**	8375	7499	5134	9210	4965	3902	7113	4043
**Aquabacterium**	6961	5728	3541	8070	4700	3179	4826	3619
**Mitsuaria**	6388	4955	2968	5577	3202	2490	4275	1913
**Paucibacter**	4447	5416	3154	5916	2127	2856	5399	4152
**Roseateles**	4059	3801	2389	5367	2231	2019	3709	2361
**Achromobacter**	33403	30773	22674	40314	17138	17419	29834	22627
**Bordetella**	31927	29465	24133	41870	15775	19656	28811	20678
**Pigmentiphaga**	3291	2899	2447	3536	1544	1597	2561	1540
**Alcaligenes**	3125	3647	3782	9841	1441	2538	3439	5709
**Castellaniella**	2898	2095	1958	2896	1113	1275	2036	1191
**Orrella**	2370	2103	1564	2974	1203	1464	2228	1393
**Pusillimonas**	1357	1094	876	1625	568	841	1215	1465
**Advenella**	949	846	1403	1059	433	645	894	1496
**Oligella**	336	142	645	124	88	129	142	433
**Taylorella**	192	165	161	78	43	202	105	288
**Paenalcaligenes**	122	94	73	112	30	75	84	446
**Basilea**	117	98	254	45	59	131	103	425
**Massilia**	25884	138986	30106	281865	53527	27453	121951	17248
**Janthinobacterium**	17401	49221	35649	1237082	159598	23175	45713	429464
**Herbaspirillum**	9875	11723	7114	18094	5861	5898	11445	7469
**Collimonas**	5441	6990	4823	11269	3538	3766	7321	5883
**Oxalobacter**	1228	761	274	243	155	687	581	383
**Herminiimonas**	1099	1268	767	1976	639	751	1171	1542
**Undibacterium**	608	547	417	891	253	377	652	969
**Sutterella**	1538	2256	569	559	415	2057	688	297
**Thauera**	42137	22057	47484	33662	9912	12682	16811	18759
**Azoarcus**	29849	17111	14502	21463	8760	11544	14085	12237
**Dechloromonas**	29222	13267	2884	7081	7560	4746	5052	9515
**Azospira**	12307	5084	2629	5880	7629	5475	4428	2457
**Aromatoleum**	3524	1964	1914	2613	1044	1428	1554	1485
**Aquaspirillum**	11046	3379	1552	6540	9857	5467	6044	3984
**Chromobacterium**	8288	6231	4580	7663	4071	4945	5606	4707
**Iodobacter**	216	160	747	123	110	128	134	471
**Laribacter**	3138	1690	1239	3134	885	1924	2639	1836
**Jeongeupia**	2315	1377	1008	1863	788	1015	1259	865

**Aquitalea**	2250	1395	810	1376	791	965	1337	1428
**Pseudogulbenkiania**	1876	1207	828	1536	802	909	1217	930
**Vogesella**	1772	1302	947	1728	803	1061	1305	1189
**Microvirgula**	1650	6038	743	2344	906	8861	1670	1802
**Neisseria**	6275	4674	3852	3839	2486	4828	5731	48488
**Vitreoscilla**	3527	2333	2160	3681	1243	2165	3334	14739
**Crenobacter**	1560	1017	672	1135	621	674	829	600
**Eikenella**	848	465	736	532	229	731	427	4118
**Kingella**	203	113	77	96	46	177	98	1385
**Snodgrassella**	187	135	94	133	63	169	205	559
**Simonsiella**	97	144	67	55	23	72	103	721
**Sulfuritalea**	3108	2323	1215	1708	1092	1078	1236	1615
**Methyloversatilis**	2597	1666	1109	1846	959	1027	1286	1130
**Nitrosomonas**	3309	2289	3318	2355	997	2164	2837	2159
**Nitrosospira**	1659	1061	1149	1277	418	1524	2772	1007
**Sulfuritortus**	3346	1708	1141	2736	827	1473	1572	743
**Thiobacillus**	1545	1423	789	1117	550	719	800	582
**Sideroxydans**	967	671	399	664	360	525	523	529
**Sulfuriferula**	964	638	402	1042	285	905	1895	1553
**Sulfuricella**	765	559	307	514	259	456	411	530
**Gallionella**	685	469	406	738	216	479	470	683
**Ferriphaselus**	612	446	298	475	265	385	334	518
**Methylovorus**	799	563	504	791	281	539	582	811
**Methylobacillus**	710	605	367	678	252	492	493	475
**Methylotenera**	253	252	752	171	86	252	329	1021
**Candidatus Methylopumilı**	221	161	254	131	116	198	160	617
**Methylophilus**	205	176	120	145	71	137	121	330
**Candidatus Accumulibact**	4572	2693	1343	2811	1597	1747	1990	2740
**Candidatus Kinetoplastiba**	250	171	363	104	143	233	219	272
**Candidatus Profftella**	35	34	5	12	5	34	13	32
**Candidatus Tremblaya**	21	1	1	0	0	2	2	3
**Candidatus Nasuia**	6	5	0	1	0	1	1	9
**Sphingopyxis**	449715	478936	241500	311381	201253	261738	276576	52743
**Sphingobium**	182771	127888	44622	66615	53582	74606	66456	14898
**Sphingomonas**	135456	140573	71474	93070	75580	69807	77493	15133
**Novosphingobium**	45116	37791	27249	36756	22324	23940	21569	7602
**Blastomonas**	26400	10666	6427	8594	17658	9709	14404	2262
**Sphingosinicella**	6445	6593	3260	4921	3417	3532	3785	1213
**Sphingorhabdus**	5816	4082	2843	3217	2415	3004	3894	1160
**Rhizorhabdus**	5455	5275	3169	3755	2909	3007	3190	756

**Citromicrobium**	2151	1893	1340	1332	1048	1162	1272	425
**Zymomonas**	598	619	791	1531	276	585	556	314
**Altererythrobacter**	15848	14047	11553	11652	7349	8373	8835	3421
**Erythrobacter**	15497	12834	9935	11295	7595	8438	8576	3375
**Porphyrobacter**	11769	8047	6488	7027	5584	5630	5315	2370
**Croceicoccus**	6647	5160	4024	4618	2608	3295	3305	1220
**Rhizobium**	107812	69086	53894	176433	61446	50908	56575	22551
**Agrobacterium**	49369	31409	20133	65226	22198	34265	60888	16859
**Neorhizobium**	13246	8752	7506	25210	8578	6600	7773	3695
**Sinorhizobium**	78499	62633	38566	240200	45667	55303	64386	15567
**Ensifer**	30756	18876	14701	73892	25193	36418	24042	4043
**Shinella**	71676	51318	51765	301418	46739	43017	49971	11111
**Liberibacter**	306	208	110	241	277	250	302	263
**Bosea**	133744	115627	104354	325463	230968	67955	106940	21617
**Bradyrhizobium**	82499	75080	41065	85966	102791	49578	60862	17218
**Rhodopseudomonas**	23070	16265	9575	22741	21169	10662	12913	4626
**Nitrobacter**	4157	4051	1927	6883	5204	2265	3535	953
**Oligotropha**	3505	3285	1651	3644	4836	1675	2439	615
**Afipia**	2296	2113	876	2240	3115	1137	1794	702
**Variibacter**	1656	1425	884	2016	1773	927	1177	392
**Mesorhizobium**	66158	51062	29743	96259	50310	30963	41958	14066
**Aminobacter**	16839	16089	7622	36977	14752	9372	13352	3587
**Hoeflea**	2366	1627	1256	3334	1435	1333	1555	801
**Chelativorans**	2362	2198	1592	5072	1894	1424	1805	689
**Nitratireductor**	2336	1803	1539	3260	1498	1594	1527	750
**Phyllobacterium**	1580	1133	789	2590	1048	889	961	424
**Methylobacterium**	42643	40436	20972	55474	50859	21989	24989	7120
**Methylorubrum**	8385	7590	4506	11893	7908	5231	5579	1781
**Microvirga**	5008	4185	2657	7085	4398	3000	3146	1100
**Devosia**	10889	9364	8009	13423	8654	5631	5727	2981
**Hyphomicrobium**	5334	4206	2442	4628	9439	3466	3691	1195
**Blastochloris**	4243	3437	2519	7915	3368	3230	2578	997
**Rhodoplanes**	2491	2071	1579	3245	2323	1419	1598	708
**Pelagibacterium**	1033	818	784	1532	680	679	651	378
**Rhodomicrobium**	1031	793	526	1278	620	654	570	252
**Filomicrobium**	362	311	184	382	251	286	170	112
**Maritalea**	282	248	216	278	122	246	212	290
**Xanthobacter**	8412	7408	2705	8144	7972	4740	3836	2277
**Azorhizobium**	4707	3746	2157	6055	5414	3045	2971	829
**Starkeya**	3872	3624	1937	6271	3651	2778	2442	911

**Pseudolabrys**	2423	1941	1445	3135	2270	1315	1532	651
**Pleomorphomonas**	5915	3686	3423	23954	3293	4761	3498	1092
**Methylocystis**	4089	3014	2024	3680	3600	2192	2166	900
**Methylosinus**	2112	1692	1132	2538	2166	1321	1192	479
**Ochrobactrum**	8866	5680	5135	20738	3647	8437	5398	7359
**Brucella**	2237	1774	1474	3628	1483	1440	1431	904
**Martelella**	7519	4863	4783	12724	4363	4587	4720	1994
**Aureimonas**	2518	1968	1334	3937	1719	1540	1503	538
**Hartmannibacter**	3075	2379	1664	7954	2382	2316	2239	737
**Pseudorhodoplanes**	1854	1752	1733	2220	1956	1109	1272	529
**Methyloceanibacter**	1330	1085	660	1568	875	910	827	345
**Chelatococcus**	6021	4801	3441	9596	6243	3487	3947	1225
**Methylocella**	1407	1495	755	1668	1362	939	865	352
**Methylovirgula**	1246	1051	529	1215	1143	749	749	327
**Beijerinckia**	853	955	419	841	890	605	598	317
**Parvibaculum**	1568	1768	1175	1809	952	989	1025	1084
**Anderseniella**	646	431	275	549	401	408	331	303
**Candidatus Phaeomarinob**	452	342	556	464	258	313	249	198
**Breoghania**	2016	1445	1136	3259	1295	1388	1118	509
**Cohaesibacter**	655	484	349	835	380	611	381	272
**Bartonella**	1405	1016	580	841	448	1330	1245	1384
**Paracoccus**	159716	93309	64487	114550	33489	84812	150593	30223
**Rhodobacter**	33255	15044	27048	24724	9567	14330	17775	10601
**Pannonibacter**	15673	11545	7165	18879	7404	8015	10309	1805
**Defluviimonas**	7840	5051	6790	6517	2855	3685	5897	1985
**Celeribacter**	7401	4767	4967	6472	2930	4137	5301	2570
**Rhodovulum**	6602	4331	4336	5885	2319	3789	4371	2126
**Sulfitobacter**	6382	3679	4912	4693	2209	3370	3987	3102
**Tabrizicola**	6339	2908	4751	4110	1479	1940	3092	3836
**Phaeobacter**	5243	2959	4600	4074	1856	2968	3460	2603
**Gemmobacter**	4140	1762	3891	2730	1100	1450	2444	2946
**Yangia**	4128	2640	2682	3880	1698	2472	2886	1421
**Ruegeria**	3226	1911	2642	2327	1102	1628	2058	1435
**Thalassococcus**	2963	1658	2157	2330	1140	1417	1831	1079
**Ketogulonicigenium**	2465	1515	2105	2001	823	1544	1926	1000
**Labrenzia**	2400	1629	1889	3139	1313	1469	1538	881
**Sagittula**	2277	1454	1398	2148	794	1206	1314	707
**Pelagibaca**	2245	1377	1583	2202	965	1247	1564	859
**Salipiger**	2237	1484	1377	2385	1030	1298	1444	627
**Leisingera**	2223	1280	1662	1835	896	1170	1512	1016

**Confluentimicrobium**	2152	1105	1640	1832	637	986	1285	868
**Thioclava**	2150	1073	1450	1902	735	1194	1373	685
**Marinovum**	2105	1315	1769	1990	853	1206	1533	774
**Dinoroseobacter**	1689	951	1167	1240	659	747	947	588
**Brevirhabdus**	1664	934	1081	1140	612	700	890	505
**Yoonia**	1599	717	1197	1151	536	735	1026	656
**Roseibacterium**	1587	1588	1084	1390	603	975	1151	520
**Silicimonas**	1547	1004	1055	1401	701	794	1009	480
**Roseovarius**	1367	777	1104	1040	463	681	878	667
**Stappia**	1230	1030	766	2118	818	889	847	391
**Antarctobacter**	1159	659	787	960	396	667	785	542
**Epibacterium**	1142	684	763	813	398	616	702	525
**Octadecabacter**	1071	610	983	845	341	569	744	685
**Roseobacter**	1051	865	706	756	443	573	851	522
**Tateyamaria**	951	525	659	721	365	447	476	369
**Rhodobaca**	920	584	722	735	303	467	718	613
**Jannaschia**	801	417	628	637	336	436	493	348
**Sedimentitalea**	435	305	393	323	162	296	331	282
**Pseudovibrio**	362	276	306	415	223	339	266	238
**Halocynthiibacter**	312	144	212	190	131	149	167	169
**Marivivens**	289	400	197	275	148	147	286	164
**Hyphomonas**	1887	1809	1162	1611	1026	1187	1177	624
**Glycocaulis**	800	672	360	675	337	524	459	258
**Maricaulis**	591	584	378	720	310	439	347	188
**Hirschia**	125	101	168	59	29	109	63	118
**Brevundimonas**	158265	313413	217941	283348	111888	66683	81594	44327
**Caulobacter**	42663	58210	17729	36679	28130	20476	25436	9836
**Phenylobacterium**	7126	10638	4077	7211	7515	3865	5688	2481
**Asticcacaulis**	1742	1936	1268	1628	919	1134	1116	596
**Azospirillum**	19924	15906	11027	21841	11494	12629	12323	5611
**Magnetospirillum**	7193	5003	3614	6576	3846	4648	4012	1941
**Rhodospirillum**	2972	2556	1801	3578	1809	2123	2106	913
**Tistrella**	2471	2048	1470	2794	1451	1463	1666	714
**Nitrospirillum**	2434	2410	1402	3150	1638	2152	1657	735
**Niveispirillum**	2000	2806	1161	2081	1885	1361	1343	629
**Thalassospira**	1836	1514	1238	1612	769	1333	1211	835
**Indioceanicola**	1311	1222	776	1262	837	976	877	431
**Pararhodospirillum**	934	779	592	1269	787	737	617	345
**Magnetospira**	471	374	279	549	255	356	282	191
**Haematospirillum**	163	126	87	168	77	144	96	70

**Candidatus Endolissoclinu**	74	88	106	36	29	64	55	82
**Roseomonas**	9933	6795	5249	10634	6549	5158	6985	2779
**Komagataeibacter**	3793	3118	2631	3800	1861	3178	2542	1390
**Acetobacter**	2205	1852	1615	2350	1081	2594	1724	1340
**Acidiphilium**	1957	1574	1283	2201	1272	1088	1221	541
**Acidisphaera**	1395	1128	959	1417	848	788	869	324
**Gluconacetobacter**	1233	1122	820	1359	686	1486	843	353
**Granulibacter**	1174	943	714	1135	584	824	690	381
**Gluconobacter**	1099	1012	633	983	579	1039	652	477
**Kozakia**	509	337	291	441	223	378	267	226
**Neoasaia**	490	405	277	391	212	417	261	140
**Asaia**	236	205	219	226	103	250	155	146
**Parasaccharibacter**	217	174	95	141	70	214	85	80
**Commensalibacter**	72	62	27	15	23	89	40	71
**Polymorphum**	3068	1872	1660	5617	1705	1847	1905	669
**Phreatobacter**	2761	2527	2755	4110	2952	1689	1723	665
**Micavibrio**	655	588	825	596	262	509	481	292
**Candidatus Puniceispirillu**	159	109	109	86	50	128	79	101
**Rickettsia**	670	464	256	263	160	639	579	430
**Orientia**	92	91	148	43	42	85	60	65
**Candidatus Phycoricketts**	67	55	36	35	14	65	38	29
**Wolbachia**	308	184	213	108	74	249	282	239
**Ehrlichia**	192	135	63	91	39	221	144	214
**Anaplasma**	87	74	31	16	20	111	48	54
**Neorickettsia**	67	44	21	56	7	51	29	29
**Candidatus Midichloria**	35	48	8	41	10	34	19	23
**Candidatus Fokinia**	28	17	9	6	6	9	9	6
**Candidatus Pelagibacter**	326	320	111	183	91	266	598	419
**Candidatus Fonsibacter**	117	153	27	65	74	125	86	553
**Magnetococcus**	293	181	156	141	61	281	161	176
**Parvularcula**	271	193	116	201	139	188	130	103
**Candidatus Nucleicultrix**	110	47	21	58	35	59	45	98
**Candidatus Paracaedibact**	100	76	330	63	36	63	51	53
**Desulfovibrio**	72801	20786	11924	17251	13481	33480	37447	5855
**Pseudodesulfovibrio**	2329	1608	999	1150	757	2465	1308	707
**Lawsonia**	129	67	30	20	26	172	59	72
**Desulfomicrobium**	1496	903	573	782	368	1485	782	819
**Desulfohalobium**	208	169	79	177	68	183	124	58
**Myxococcus**	5382	5084	3697	4458	42988	4210	3651	1888
**Corallococcus**	1741	1640	1015	1452	1106	1472	1190	652

**Archangium**	1346	1280	1016	1357	729	1061	951	431
**Cystobacter**	1244	1191	736	1199	723	886	819	453
**Stigmatella**	955	915	577	879	579	740	659	412
**Melittangium**	854	829	518	720	446	724	592	322
**Anaeromyxobacter**	3716	3575	2618	2770	1762	3081	2294	986
**Vulgatibacter**	724	585	396	538	292	537	444	245
**Sorangium**	6417	6079	4459	5727	3373	4436	4319	1842
**Chondromyces**	1018	1060	773	1021	521	850	750	334
**Pajaroellobacter**	30	30	16	24	8	31	11	21
**Sandaracinus**	1588	1538	1134	1202	819	1083	1013	381
**Haliangium**	1627	1570	1088	1563	828	1258	1143	730
**Geobacter**	6789	4901	3085	3761	2237	6653	3494	1898
**Geoalkalibacter**	553	354	243	263	146	573	251	179
**Pelobacter**	1882	1407	881	866	574	2070	990	750
**Desulfuromonas**	1255	1450	818	758	508	1317	904	539
**Desulfococcus**	925	686	496	574	287	867	473	327
**Desulfobacter**	508	299	636	145	166	607	307	225
**Desulfatibacillum**	462	296	183	177	123	446	254	162
**Desulfobacula**	247	210	228	98	83	354	193	112
**Desulfobacterium**	194	178	124	81	60	234	93	123
**Desulfobulbus**	726	454	251	318	174	550	414	154
**Desulfurivibrio**	463	413	260	492	242	547	474	263
**Desulfotalea**	250	363	98	675	107	510	547	196
**Desulfocapsa**	159	180	106	122	48	177	350	65
**Syntrophus**	256	181	119	86	85	246	113	71
**Desulfomonile**	228	194	108	92	68	276	193	135
**Desulfobacca**	150	123	97	54	53	145	86	124
**Syntrophobacter**	452	316	250	241	166	464	212	168
**Desulfarculus**	1037	732	483	668	345	1005	532	337
**Bradymonas**	488	412	285	368	166	423	301	131
**Desulfurella**	104	86	35	35	25	108	66	66
**Hippea**	93	78	35	25	17	104	113	31
**Candidatus Desulfofervidu**	121	67	80	48	31	90	97	55
**Arcobacter**	16301	3525	4144	4532	7171	5125	11221	358481
**Aliiarcobacter**	227	80	54	68	88	120	146	3252
**Campylobacter**	9209	5364	4466	2735	2028	14133	6578	4816
**Sulfurospirillum**	1726	1168	719	1422	2685	1566	1477	2302
**Helicobacter**	1856	1351	1513	976	757	1727	1662	1807
**Sulfuricurvum**	409	132	47	56	121	192	91	195
**Sulfurimonas**	330	237	123	132	83	285	160	1240

**Wolinella**	112	127	64	70	104	108	73	50
**Nitratifractor**	198	170	196	93	39	260	159	66
**Nautilia**	291	208	120	81	57	287	228	161
**Sulfurovum**	141	136	58	43	57	225	99	91
**Nitratiruptor**	77	42	25	112	19	73	42	60
**Bacteriovorax**	3728	546	826	803	518	379	571	361
**Halobacteriovorax**	418	241	672	98	99	272	166	344
**Bdellovibrio**	2231	1290	1219	748	577	1084	1022	491
**Hydrogenophilus**	4389	581	225	1320	494	1334	884	277
**Acidithiobacillus**	3287	1676	1305	3193	648	2163	2160	1984
**Mariprofundus**	412	246	246	213	156	388	279	199
**Faecalibacterium**	1834440	768956	468748	300790	449834	2120253	608140	136167
**Ruminococcus**	106606	35763	39703	8717	6917	374392	30438	3220
**Flavonifractor**	31147	28451	14465	6419	7519	58547	14088	2700
**Ethanoligenens**	4876	3208	1442	915	843	7476	2151	650
**Caproiciproducens**	4484	2697	1414	829	611	6920	2064	699
**Acutalibacter**	2	1	1	0	2	4	0	1
**Lachnoclostridium**	136804	54890	32232	23728	43475	172774	65896	7782
**Anaerostipes**	95153	53465	33849	50516	23556	150250	41182	10727
**Roseburia**	91714	64520	32725	18949	20122	153149	49064	6744
**Blautia**	55003	36936	20558	13573	21296	92226	28324	5925
**Butyrivibrio**	11252	7737	3531	2230	2391	17207	4744	1352
**Lachnoanaerobaculum**	2947	1910	873	570	667	4324	1346	336
**Cellulosilyticum**	2382	1445	569	469	394	3106	1093	439
**Anaerotignum**	2024	1073	624	362	385	3190	909	253
**Herbinix**	1781	1317	561	363	481	2660	868	261
**Eubacterium**	138736	148497	53136	32232	41506	235772	80061	11519
**Acetobacterium**	1216	752	668	305	400	1609	713	706
**Intestinimonas**	42696	31155	13718	6914	5269	75108	20427	4148
**Monoglobus**	4129	2917	1179	920	765	6715	3817	995
**Clostridium**	60649	42248	21344	14885	21548	73365	33727	19187
**Mordavella**	15149	9188	4765	3804	3976	29023	7418	1651
**Alkaliphilus**	1153	618	406	184	227	1136	498	567
**Geosporobacter**	979	493	740	369	176	991	377	519
**Candidatus Arthromitus**	430	297	140	140	88	450	251	200
**Oscillibacter**	63294	56306	29374	12689	10638	156479	38731	7574
**Clostridioides**	46052	26973	15095	9404	9949	75928	23848	4775
**Acetoanaerobium**	9774	490	2923	319	354	1186	779	1803
**Paeniclostridium**	1205	721	311	229	311	1453	961	402
**Peptoclostridium**	1138	477	1119	196	156	1058	313	645

**Filifactor**	664	424	225	177	163	896	379	119
**Christensenella**	25516	18857	8721	4865	4186	44010	12640	2669
**Desulfitobacterium**	3117	1568	1386	678	550	3406	1285	652
**Syntrophobotulus**	2242	362	245	167	154	845	296	127
**Desulfosporosinus**	1935	1117	1144	470	390	2019	1035	439
**Desulfotomaculum**	1712	1008	584	544	425	1938	751	494
**Dehalobacterium**	1677	708	787	327	246	1710	613	384
**Dehalobacter**	1197	690	452	258	245	1650	669	262
**Desulfallas**	739	417	241	175	143	913	315	154
**Desulfofarcimen**	448	260	188	157	99	421	218	105
**Candidatus Desulforudis**	386	329	247	169	84	520	202	150
**Thermincola**	358	184	172	140	62	416	164	125
**Hungateiclostridium**	2896	1501	1345	637	531	3245	1000	637
**Mageeibacillus**	1417	919	439	332	275	1806	723	222
**Pseudoclostridium**	1127	638	356	236	231	1364	450	140
**Ruminiclostridium**	844	511	265	155	166	1167	388	200
**Thermoclostridium**	749	490	274	147	166	1178	330	136
**Fastidiosipila**	570	326	111	98	77	601	259	193
**Aminipila**	2126	1050	614	485	392	2654	861	357
**Mogibacterium**	1571	749	411	369	326	1881	671	214
**Thermaerobacter**	696	589	378	428	220	924	484	213
**Carboxydocella**	512	283	167	125	139	542	212	92
**Symbiobacterium**	1186	939	630	523	308	1496	657	265
**Heliobacterium**	1143	696	430	311	211	1301	473	195
**Syntrophomonas**	268	141	105	69	48	295	115	163
**Syntrophothermus**	168	118	45	40	36	214	68	107
**Caldicellulosiruptor**	1993	1212	795	583	576	2305	1075	626
**Thermoanaerobacterium**	1731	902	728	447	766	1597	799	443
**Thermosediminibacter**	364	182	105	82	36	407	145	150
**Thermoanaerobacter**	1073	608	372	318	251	1109	488	556
**Moorella**	721	312	197	278	149	649	296	138
**Ammonifex**	245	178	143	107	73	332	107	49
**Tepidanaerobacter**	356	180	94	82	63	436	169	211
**Thermacetogenium**	336	217	149	126	89	391	156	86
**Caldanaerobacter**	315	200	94	77	51	311	125	105
**Thermanaeromonas**	179	103	120	49	50	215	96	58
**Carboxydothermus**	147	98	54	33	37	173	72	43
**Mahella**	589	263	153	94	78	574	166	208
**Thermodesulfobium**	152	198	47	84	48	171	124	106
**Halanaerobium**	509	407	176	128	133	507	378	286

**Halocella**	331	202	83	113	71	380	142	104
**Halothermothrix**	148	118	36	37	36	160	76	68
**Halobacteroides**	356	197	92	81	59	277	470	170
**Acetohalobium**	352	150	93	81	72	305	129	80
**Anoxybacter**	357	211	129	127	62	338	158	106
**Natranaerobius**	229	134	61	42	47	206	205	90
**Lactobacillus**	109132	18997	18877	22959	165652	34166	34892	14491
**Pediococcus**	5240	510	934	982	991	871	480	825
**Streptococcus**	61959	19556	21987	15664	22571	51253	37344	33483
**Lactococcus**	9212	14681	9311	9205	8212	15436	13572	515659
**Enterococcus**	47805	18690	33515	227459	303323	42718	32440	19397
**Tetragenococcus**	958	571	2081	380	220	1670	770	1089
**Vagococcus**	534	286	1285	216	153	799	342	766
**Melissococcus**	284	127	397	57	96	378	254	291
**Carnobacterium**	1582	948	10687	4772	561	3233	2267	8333
**Jeotgalibaca**	1091	591	14600	866	406	3653	1551	7192
**Marinilactibacillus**	284	199	1348	138	112	565	245	554
**Leuconostoc**	1099	875	2050	572	552	1071	680	2476
**Weissella**	661	415	778	208	368	701	532	660
**Oenococcus**	385	300	591	191	142	477	375	236
**Aerococcus**	1323	810	7345	750	1180	2128	1373	4214
**Bacillus**	30526	18269	24339	11460	10593	30296	18251	19042
**Anoxybacillus**	2813	832	818	1146	4163	1645	1101	617
**Geobacillus**	2657	1560	1828	965	1540	2718	1656	1075
**Virgibacillus**	1859	1153	1305	607	512	2093	1088	1330
**Lysinibacillus**	1752	1032	1061	531	438	1793	978	1412
**Oceanobacillus**	910	657	636	291	234	1044	739	492
**Halobacillus**	882	693	823	824	212	1081	640	686
**Fictibacillus**	582	363	270	187	168	584	323	249
**Lentibacillus**	363	155	102	81	67	266	141	83
**Parageobacillus**	362	166	355	156	311	310	174	189
**Paraliobacillus**	306	148	158	58	51	249	111	167
**Aeribacillus**	304	176	145	104	179	388	150	110
**Salimicrobium**	289	162	184	77	56	320	109	130
**Terribacillus**	259	189	309	136	57	352	167	253
**Amphibacillus**	222	151	568	68	61	312	126	189
**Paenibacillus**	26596	18425	17066	9349	7097	34451	14316	9824
**Brevibacillus**	2807	1895	1475	944	699	2949	1629	1275
**Thermobacillus**	1314	1001	528	467	316	1623	643	243
**Cohnella**	1049	722	495	464	227	1264	515	316

**Aneurinibacillus**	754	386	302	735	187	905	474	323
**Staphylococcus**	8277	4847	8043	2809	2376	7408	5467	6581
**Macrococcus**	566	332	503	200	117	610	274	489
**Salinicoccus**	237	146	139	59	49	288	144	86
**Jeotgalicoccus**	181	123	93	49	53	266	93	80
**Auricoccus**	133	114	199	35	31	134	155	72
**Planococcus**	2436	2178	3512	1188	598	2943	1404	2427
**Sporosarcina**	1439	911	1170	667	373	1813	1097	874
**Solibacillus**	721	381	497	165	174	754	380	401
**Kurthia**	444	1253	293	168	85	406	303	881
**Jeotgalibacillus**	395	236	189	91	64	423	301	103
**Ureibacillus**	362	195	161	72	69	306	144	192
**Rummeliibacillus**	207	154	649	70	35	174	132	176
**Paenisporosarcina**	159	79	148	112	43	132	215	102
**Alicyclobacillus**	1541	713	354	838	1662	1261	905	224
**Tumebacillus**	1470	1064	599	484	490	1778	710	364
**Kyrpidia**	333	251	212	159	95	455	160	120
**Listeria**	2142	1274	2600	670	512	2426	1411	2608
**Brochothrix**	287	160	274	377	78	290	202	440
**Exiguobacterium**	932	771	2156	597	281	1182	639	972
**Gemella**	892	550	300	289	211	864	424	436
**Laceyella**	209	171	182	76	65	297	139	107
**Novibacillus**	205	130	129	65	41	256	96	93
**Sporolactobacillus**	427	219	201	95	83	430	138	172
**Veillonella**	97926	13653	4294	19520	17109	22463	30962	3579
**Dialister**	48394	15197	9633	7037	5021	28288	15363	4184
**Megasphaera**	11725	7717	10082	2079	4427	10910	16430	6749
**Negativicoccus**	1249	649	455	364	381	771	679	186
**Phascolarctobacterium**	50118	21004	6828	12175	27479	65509	12642	6199
**Acidaminococcus**	6301	6370	5393	1832	2426	12969	14211	1156
**Megamonas**	17569	1347	596	856	477	1675	2949	671
**Selenomonas**	8409	7620	2986	2562	1590	10432	6569	3452
**Pelosinus**	2112	701	409	535	425	1118	718	670
**Methylomusa**	1282	449	241	377	206	777	388	207
**Faecalitalea**	24460	12855	7972	5395	6312	38398	12105	2628
**Faecalibaculum**	1726	1098	740	444	451	2629	855	217
**Turicibacter**	1438	1077	445	314	420	2056	710	355
**Erysipelothrix**	1223	567	559	578	179	1001	447	419
**Erysipelatoclostridium**	61	10	6	27	36	81	38	3
**Peptoniphilus**	1606	866	609	350	668	1481	1129	359

**Finegoldia**	1324	513	390	331	446	790	573	246
**Parvimonas**	873	478	267	251	199	843	406	255
**Murdochiella**	806	576	330	186	172	1216	485	142
**Anaerococcus**	699	418	183	176	291	729	394	165
**Soehngenia**	555	327	419	100	112	584	218	348
**Sporanaerobacter**	1215	443	338	192	180	1046	350	430
**Gottschalkia**	656	352	203	123	100	670	355	209
**Ezakiella**	712	240	139	93	249	289	248	95
**Ndongobacter**	717	496	231	218	123	1128	355	108
**Limnochorda**	617	476	313	308	190	711	297	158
**Bifidobacterium**	691410	139606	139402	271361	208482	358783	135467	80834
**Gardnerella**	13129	2786	1747	4650	5258	2004	4046	360
**Parascardovia**	338	223	183	203	679	498	184	77
**Scardovia**	236	130	67	60	66	279	95	29
**Microbacterium**	188515	395489	144098	2108052	386812	189936	1014282	40738
**Leucobacter**	7917	8132	16043	24245	5786	3404	8001	2388
**Agromyces**	7771	12989	10286	21332	7294	5503	10423	3571
**Leifsonia**	4819	7921	5986	11531	4302	3723	7793	2239
**Rathayibacter**	3576	4807	4286	7228	2893	2607	3929	1643
**Clavibacter**	3245	4340	4320	6163	2972	2312	3894	1761
**Agrococcus**	2918	3716	4086	5867	2383	1903	3284	1112
**Curtobacterium**	2873	3866	3742	5564	2199	2002	3194	1249
**Cryobacterium**	2772	3987	3183	6074	2306	2036	3469	1654
**Lysinimonas**	2685	3247	3653	5343	2405	1647	3263	1111
**Microterricola**	2606	4482	3363	6353	2278	1905	3309	1882
**Frondihabitans**	1938	2767	2668	5110	1712	1525	2498	1094
**Plantibacter**	1719	2885	2767	4730	1488	1223	2337	966
**Gryllotalpicola**	1260	2152	1568	2804	1212	922	2095	590
**Salinibacterium**	1070	1169	1131	3116	1112	756	2013	1040
**Microcella**	1008	1282	1391	3153	962	748	1711	553
**Mycetocola**	627	779	763	1355	433	485	693	373
**Humibacter**	519	611	659	1094	371	325	631	213
**Cnuibacter**	441	621	434	1163	436	323	648	247
**Aurantimicrobium**	330	286	275	473	186	255	234	221
**Rhodoluna**	164	106	145	121	79	92	85	107
**Candidatus Aquiluna**	65	54	39	83	26	88	45	53
**Pontimonas**	137	85	75	162	59	159	73	53
**Arthrobacter**	9729	11123	9147	11715	5485	6906	8116	5510
**Kocuria**	5006	5733	7577	7761	4071	4005	5161	2198
**Rothia**	2931	1531	750	1173	1859	1570	2199	558

**Pseudarthrobacter**	2563	3923	2905	4151	1846	2251	2440	1047
**Zhihengliuella**	1709	3074	1623	11718	1133	1071	3401	296
**Micrococcus**	1488	1588	2488	2959	1766	1242	1493	782
**Glutamicibacter**	1349	1234	1399	2712	835	1157	1317	707
**Sinomonas**	1034	1408	1516	1908	853	806	1125	425
**Auritidibacter**	339	217	249	393	139	158	309	79
**Neomicrococcus**	290	282	294	286	167	242	172	169
**Psychromicrobium**	240	211	203	326	161	163	158	135
**Renibacterium**	145	118	92	98	54	100	107	66
**Paenarthrobacter**	110	136	140	172	131	73	136	57
**Serinicoccus**	3116	3335	5727	3793	2109	2006	2761	962
**Janibacter**	2585	2805	3458	3498	2026	1628	2529	1109
**Arsenicicoccus**	2010	1676	2688	1813	1177	1080	1587	611
**Phycicoccus**	1654	2221	1709	1624	1345	971	1430	736
**Ornithinimicrobium**	1635	1578	2411	1835	1166	980	1324	438
**Intrasporangium**	1561	1874	2253	1797	1332	1112	1326	659
**Cellulosimicrobium**	2675	2726	7308	3654	1910	2120	2333	1180
**Isoptericola**	2424	2403	5330	3099	1993	1924	2073	964
**Xylanibacterium**	1227	1372	2350	1933	1020	961	1167	429
**Xylanimicrobium**	1192	1223	2868	1537	875	880	1086	365
**Xylanimonas**	1041	1215	2354	1629	860	792	1061	385
**Cellulomonas**	5874	6302	21772	8761	4961	4769	5192	2250
**Paraoerskovia**	787	842	1858	1072	600	659	684	318
**Brachybacterium**	5459	5884	7414	7667	3810	4485	4960	1697
**Dermabacter**	290	225	470	710	188	227	268	111
**Devriesea**	215	195	179	232	134	207	209	102
**Miniimonas**	2528	2664	5326	7047	2032	2003	4621	1060
**Beutenbergia**	1327	1484	2734	2222	1008	945	1255	416
**Brevibacterium**	3706	4317	5205	12435	2693	2940	4826	1396
**Kytococcus**	1137	1417	2030	2565	795	745	1223	363
**Dermacoccus**	1095	1040	1771	1628	836	791	1055	672
**Luteipulveratus**	1007	1119	1159	1170	812	699	939	449
**Sanguibacter**	1264	1224	3234	1934	898	1102	1097	621
**Georgenia**	1139	964	1921	1582	792	945	1008	386
**Austwickia**	690	494	1009	676	438	507	604	229
**Dermatophilus**	271	194	297	167	158	195	165	136
**Jonesia**	220	160	333	295	107	161	264	97
**Tropheryma**	44	14	14	11	5	25	14	10
**Mycobacterium**	120027	72439	23556	57791	194806	46079	73063	16920
**Mycolicibacterium**	76707	48933	16254	41728	141509	30280	51943	12787

**Mycobacteroides**	8484	5312	2047	4851	12488	3934	6295	1568
**Mycolicibacter**	7407	4774	1755	3962	8466	3446	5778	1135
**Hoyosella**	376	284	272	355	347	254	318	171
**Rhodococcus**	21602	24193	15306	31121	28731	14103	17470	8913
**Nocardia**	14805	12707	9643	13737	14370	8964	11147	4394
**Corynebacterium**	21785	17806	19373	20841	12243	19357	15559	7285
**Gordonia**	15236	11381	8543	11861	12338	7180	12110	3443
**Dietzia**	5134	2714	3280	3145	2513	2138	2548	2270
**Tsukamurella**	3221	2883	2624	3184	3457	1729	2521	1558
**Segniliparus**	423	497	255	327	341	388	317	143
**Lawsonella**	280	207	160	180	259	1439	179	68
**Micropruina**	43853	48015	33893	21004	30226	27926	55659	3846
**Nocardioides**	8681	9944	9601	9341	6455	5668	7706	2860
**Aeromicrobium**	5581	5097	6817	6052	3802	3582	4577	1862
**Friedmanniella**	4822	5743	7354	6270	3953	3319	5358	1158
**Pimelobacter**	2038	2331	2174	2332	1471	1270	1903	606
**Kribbella**	1838	2043	2066	2152	1333	1196	1709	461
**Marmoricola**	1443	1799	1817	1698	1161	903	1304	614
**Actinopolymorpha**	1152	1197	1295	1335	970	811	1044	361
**Tessaracoccus**	27056	25874	154037	42229	15644	30860	35589	6288
**Microlunatus**	17434	7418	8026	10917	3946	5294	16262	1746
**Propionibacterium**	7112	7736	11555	13742	7250	6235	8209	1802
**Acidipropionibacterium**	6133	5793	9581	7755	5351	4511	6572	1373
**Auraticoccus**	2263	2778	3984	3273	1762	1572	2398	561
**Cutibacterium**	2068	1814	2996	2137	1725	1822	1878	612
**Pseudopropionibacterium**	1939	1931	5584	2709	1420	1698	1845	450
**Propionimicrobium**	255	199	126	143	191	409	187	75
**Streptomyces**	101203	102004	94520	111361	73507	73500	84228	36807
**Kitasatospora**	4113	4184	3520	4265	2667	2946	3559	1375
**Streptacidiphilus**	995	855	761	925	593	817	812	329
**Amycolatopsis**	10267	10412	8046	11435	7968	6902	8451	3415
**Pseudonocardia**	9095	8547	7151	9080	6605	5312	7990	2618
**Actinoalloteichus**	3511	3133	2619	4562	2141	2367	2852	1128
**Saccharomonospora**	3120	3171	2734	3256	2402	2395	2650	1157
**Actinosynnema**	2141	2049	1850	2328	1472	1596	1806	853
**Kutzneria**	1589	1492	1325	1498	1165	1000	1253	479
**Lentzea**	1568	1492	1755	1576	1062	953	1171	451
**Saccharothrix**	1367	1429	1174	1546	1013	937	1238	580
**Kibdelosporangium**	1348	1494	972	1389	955	882	1084	476
**Saccharopolyspora**	1328	1461	1369	1562	1037	843	1104	418

**Allokutzneria**	1305	1250	1084	1527	866	867	974	385
**Alloactinosynnema**	1195	1119	953	1232	920	780	986	399
**Prauserella**	769	691	726	896	562	620	620	269
**Micromonospora**	17614	18358	15891	20013	12088	11762	14858	5732
**Actinoplanes**	9763	10957	8141	10992	6751	6788	7877	3184
**Plantactinospora**	2434	2624	2247	2586	1609	1689	2000	808
**Salinispora**	940	817	811	952	673	683	811	357
**Verrucosispora**	873	788	764	930	664	630	694	296
**Actinomyces**	13819	9063	13004	10705	7588	9747	8389	3062
**Flaviflexus**	1793	923	1685	2088	619	832	928	327
**Schaalia**	1319	670	1037	725	755	1208	821	223
**Trueperella**	826	625	541	458	401	889	539	195
**Actinotignum**	356	189	242	266	1197	300	2894	116
**Actinobaculum**	292	188	204	146	96	234	137	90
**Mobiluncus**	249	107	102	102	115	240	126	55
**Arcanobacterium**	241	176	129	125	103	267	145	65
**Nakamurella**	17670	27840	2957	6358	11649	7590	10244	5305
**Streptosporangium**	2825	2874	2484	2824	2014	2189	2444	960
**Nonomuraea**	2415	2451	2286	2591	1675	1651	1916	806
**Nocardiopsis**	2794	2632	2658	3038	1784	2118	2133	935
**Streptomonospora**	897	824	836	954	576	603	695	297
**Thermobifida**	420	416	328	355	267	320	341	133
**Actinomadura**	1258	1254	1291	1117	806	867	929	473
**Thermomonospora**	1047	964	873	862	689	798	798	403
**Frankia**	6047	5640	5128	6391	4312	4260	4852	2101
**Jatrophihabitans**	577	645	432	667	454	454	555	245
**Modestobacter**	1463	1624	1440	1593	1083	990	1261	528
**Geodermatophilus**	1276	1389	1165	1327	983	805	1111	492
**Blastococcus**	1161	1259	1088	1264	849	903	998	398
**Jiangella**	3731	3921	3891	4785	2611	2621	3480	1068
**Catenulispora**	1529	1494	1335	1645	1016	1088	1255	646
**Kineococcus**	1273	1213	1662	1478	857	1037	1069	616
**Stackebrandtia**	1055	980	917	1102	638	824	771	372
**Thermobispora**	803	766	838	803	549	491	643	212
**Candidatus Planktophila**	598	462	1034	201	160	598	347	2209
**Candidatus Nanopelagicu**	187	155	84	92	55	188	221	278
**Actinopolyspora**	428	476	409	465	305	336	385	162
**Acidothermus**	272	236	155	224	149	174	181	112
**Collinsella**	120084	37312	22677	19563	48884	62239	36977	10742
**Coriobacterium**	780	494	251	188	251	1066	364	131

**Olsenella**	13792	7775	4011	2524	2620	14472	5756	3581
**Parolsenella**	2366	1781	1029	526	3061	4726	1172	464
**Libanicoccus**	1937	1382	719	453	607	2357	1126	394
**Atopobium**	667	273	233	504	261	593	244	96
**Gordonibacter**	7572	4840	2759	2804	5249	9903	4473	1225
**Eggerthella**	7525	6622	2879	5465	7586	12962	5956	1187
**Adlercreutzia**	4897	4089	1990	2041	1376	19378	3241	824
**Slackia**	1992	1240	731	530	640	2658	988	314
**Denitrobacterium**	1237	790	321	273	419	1335	567	155
**Phoenicibacter**	1047	546	277	397	363	1984	607	154
**Cryptobacterium**	260	151	77	73	74	307	120	80
**Euzebya**	933	823	664	1033	549	699	669	409
**Egicoccus**	786	708	758	675	456	577	568	326
**Egibacter**	618	519	416	632	378	457	438	282
**Conexibacter**	1598	1566	1032	1662	918	1232	1159	519
**Rubrobacter**	928	863	660	699	424	955	623	271
**Acidimicrobium**	271	224	169	340	149	218	177	171
**Oscillatoria**	14913	1180	4011	1383	540	590	571	227
**Moorea**	287	251	387	177	86	241	237	208
**Cyanothece**	729	451	283	375	226	615	406	599
**Microcoleus**	205	317	90	123	52	192	124	143
**Arthrospira**	165	136	102	57	55	152	110	302
**Planktothrix**	120	110	75	139	28	89	87	495
**Trichodesmium**	107	118	60	54	47	129	167	210
**Geitlerinema**	319	215	190	174	102	278	187	134
**Crinalium**	168	96	82	51	38	117	65	147
**Geminocystis**	451	537	657	341	122	405	632	657
**Chondrocystis**	199	122	94	120	39	142	201	90
**Gloeocapsa**	108	112	69	60	33	88	98	248
**Microcystis**	262	243	251	180	98	259	338	370
**Cyanobacterium**	177	106	62	92	24	133	140	206
**Halothece**	87	103	48	30	227	90	60	135
**Candidatus Atelocyanoba**	50	61	24	13	6	59	28	164
**Synechococcus**	3187	2359	1838	1743	1058	3488	1938	1733
**Cyanobium**	933	764	590	693	397	847	610	412
**Thermosynechococcus**	209	125	86	96	65	167	102	130
**Dactylococcopsis**	52	72	40	94	24	62	197	55
**Leptolyngbya**	715	671	450	420	220	732	450	495
**Prochlorococcus**	563	465	225	272	155	531	498	662
**Pseudanabaena**	273	236	119	107	73	288	217	240

**Synechocystis**	225	166	114	95	66	211	262	112
**Acaryochloris**	182	116	66	57	56	156	85	187
**Halomicronema**	172	175	126	146	60	207	121	99
**Chamaesiphon**	150	159	106	92	48	155	109	90
**Nostoc**	1859	1347	2192	1277	522	1329	1280	1710
**Anabaena**	293	264	157	253	136	386	261	628
**Trichormus**	207	122	386	67	35	125	78	139
**Cylindrospermum**	149	126	109	58	178	204	151	79
**Calothrix**	1487	1136	1636	882	436	1297	1172	1345
**Rivularia**	210	145	106	90	42	175	172	131
**Microchaete**	87	108	672	38	26	77	77	182
**Sphaerospermopsis**	170	162	85	87	39	103	163	211
**Nodularia**	118	120	44	124	42	100	238	204
**Anabaenopsis**	82	71	39	27	21	62	43	62
**Dolichospermum**	63	68	45	116	25	71	67	80
**Raphidiopsis**	61	68	42	85	47	56	86	101
**Scytonema**	419	243	233	155	98	243	382	353
**Fischerella**	279	256	192	127	71	246	372	248
**Tolypothrix**	1	0	0	0	0	0	0	1
**Gloeobacter**	909	674	480	651	303	769	546	425
**Stanieria**	252	189	252	219	90	197	258	210
**Pleurocapsa**	146	95	57	43	39	142	62	99
**Chroococcidiopsis**	153	204	78	80	37	171	250	166
**Gloeomargarita**	124	78	94	63	31	100	75	84
**Meiothermus**	15916	1139	15692	1099	1700	2962	1073	359
**Thermus**	1612	1190	2043	1203	832	8114	888	460
**Oceanithermus**	600	477	646	492	215	671	384	171
**Marinithermus**	230	227	295	197	83	237	139	80
**Deinococcus**	9056	7491	7312	7641	3375	8289	7218	2874
**Truepera**	480	343	374	293	188	378	291	137
**Mycoplasma**	4109	2552	2945	1466	945	3152	2260	3426
**Ureaplasma**	69	97	41	30	21	90	63	67
**Candidatus Hepatoplasma**	41	36	20	18	6	65	39	48
**Spiroplasma**	1878	1016	556	508	371	1437	998	1351
**Mesoplasma**	394	258	170	192	77	327	250	464
**Entomoplasma**	268	181	118	89	54	159	222	178
**Acholeplasma**	1348	832	509	382	248	1143	885	676
**Candidatus Phytoplasma**	213	118	66	63	51	155	104	142
**Candidatus Izimaplasma**	271	142	112	51	58	166	82	123
**Dehalococcoides**	1237	708	357	215	172	1008	583	452

**Dehalogenimonas**	375	258	232	130	93	442	179	130
**Pelolinea**	489	289	192	164	129	509	204	150
**Anaerolinea**	301	230	186	170	157	347	135	114
**Brevefilum**	222	126	866	64	70	317	122	90
**Roseiflexus**	917	688	604	497	311	772	488	316
**Chloroflexus**	396	270	239	205	143	352	178	211
**Candidatus Promineofilum**	905	616	483	658	524	881	539	285
**Sphaerobacter**	620	610	406	468	267	539	352	162
**Thermomicrobium**	161	143	132	90	71	173	107	91
**Caldilinea**	482	354	272	337	185	546	274	179
**Fimbriimonas**	314	282	184	172	143	352	219	86
**Chthonomonas**	172	131	53	48	35	152	60	53
**Thermobaculum**	203	138	156	135	56	146	89	37
**Bacteroides**	1121241	526894	316994	147173	185952	920958	176632	37921
**Alistipes**	147586	145580	115989	18115	67921	198666	35764	10874
**Mucinivorans**	753	804	374	185	160	712	572	150
**Prevotella**	40658	103659	27116	6700	10062	72625	17398	4272
**Paraprevotella**	17797	14049	6500	3635	3731	11438	3852	873
**Parabacteroides**	43726	44390	30886	13880	13407	158352	18329	6612
**Tannerella**	2704	3290	1700	655	697	3184	1264	352
**Odoribacter**	16654	17881	14631	3720	4250	24171	5690	1176
**Butyricimonas**	15366	15677	8930	3422	5663	26281	4917	1299
**Porphyromonas**	3256	3908	1961	892	946	17656	1448	450
**Petrimonas**	2705	1811	2651	699	373	1501	602	204
**Fermentimonas**	949	738	599	371	164	630	598	357
**Barnesiella**	5059	8831	3340	994	1210	6253	1478	662
**Paludibacter**	3398	1348	883	764	319	816	917	681
**Muribaculum**	1963	3577	1232	450	443	2118	612	182
**Proteiniphilum**	1439	1580	1082	469	298	1083	591	249
**Candidatus Azobacteroide**	390	323	218	179	82	311	190	200
**Draconibacterium**	802	1059	698	615	220	691	835	447
**Alkalitalea**	378	425	302	251	101	237	350	246
**Salinivirga**	276	329	224	217	77	280	314	205
**Chryseobacterium**	125535	146556	133353	285209	27541	58781	228942	256387
**Flavobacterium**	71969	167145	70921	346394	66037	58190	758629	126960
**Cloacibacterium**	69689	44001	208941	40793	16979	18882	60709	96479
**Elizabethkingia**	9660	14907	5709	10325	1627	16083	13900	10159
**Polaribacter**	5129	9629	4453	17973	2464	3981	34335	13932
**Capnocytophaga**	4395	5582	4704	8069	1658	3654	12931	5039
**Myroides**	3348	7251	2061	6787	1589	1642	19141	4322

**Tenacibaculum**	3303	6497	3425	12917	1691	2719	24055	8934
**Riemerella**	2932	2359	2071	3100	651	1086	4304	4572
**Ornithobacterium**	2698	3130	1361	1204	595	3538	1986	1412
**Gramella**	1971	3184	2398	4783	761	1752	8284	2658
**Nonlabens**	1777	2805	2192	5091	665	1421	8958	2956
**Maribacter**	1692	3063	1961	5084	722	1578	9686	3096
**Cellulophaga**	1530	3214	1669	5827	811	1399	12655	3909
**Winogradskyella**	1521	2788	2009	6054	812	1301	9756	3964
**Formosa**	1401	2535	1392	5378	696	1173	9159	3052
**Lacinutrix**	1376	3104	1799	5156	774	1228	11193	3970
**Aquimarina**	1347	2438	1937	4557	614	1177	8584	3084
**Salegentibacter**	975	1730	986	2665	369	654	5142	1505
**Lutibacter**	959	1884	892	3903	495	851	7688	2874
**Dokdonia**	904	1441	809	2914	415	736	5360	1817
**Olleya**	782	1688	849	3695	423	655	6929	2306
**Aequorivita**	705	1349	848	2357	400	556	4461	1626
**Mariniflexile**	701	1679	884	3153	415	668	7570	1696
**Weeksella**	590	595	865	847	239	350	1731	8012
**Zunongwangia**	575	794	491	1409	194	416	2717	836
**Kordia**	568	1411	654	3013	322	589	4765	1518
**Arenibacter**	520	611	308	1033	155	290	1222	528
**Siansivirga**	519	986	483	1840	240	460	4187	1212
**Seonamhaeicola**	489	844	542	1995	283	464	3211	1164
**Algibacter**	489	931	420	1897	255	444	3821	1093
**Muricauda**	483	880	598	1274	195	455	2690	789
**Flavivirga**	450	721	434	1457	196	365	2179	838
**Wenyingzhuangia**	438	841	405	1701	209	365	2917	1224
**Tamlana**	433	848	418	1678	196	369	2814	1112
**Apibacter**	407	375	301	433	98	259	816	1116
**Gillisia**	385	575	249	1197	120	241	1937	838
**Euzebyella**	337	471	422	903	134	277	1348	493
**Zobellia**	326	442	323	608	104	209	844	333
**Croceibacter**	312	505	289	875	124	236	1442	791
**Psychroflexus**	290	451	299	873	121	232	1552	821
**Robiginitalea**	270	389	236	219	107	269	290	89
**Flagellimonas**	252	440	232	633	111	347	1268	395
**Sediminicola**	200	388	250	919	121	190	1347	323
**Aureitalea**	181	271	198	409	72	142	526	267
**Gilvibacter**	180	305	201	463	80	152	801	209
**Muriicola**	154	229	138	356	63	136	717	229

**Fluviicola**	562	986	788	2898	328	486	3545	1192
**Blattabacterium**	485	470	330	855	152	361	1237	851
**Owenweeksia**	320	418	620	585	111	291	738	324
**Candidatus Sulcia**	109	97	48	116	21	38	84	177
**Candidatus Walczuchella**	15	10	19	26	5	22	27	22
**Ichthyobacterium**	70	76	54	132	17	53	164	158
**Runella**	2566	1732	962	1030	254	577	1487	891
**Spirosoma**	2232	2576	1329	1388	605	1726	2058	795
**Dyadobacter**	1401	1547	530	589	517	704	710	193
**Fibrella**	933	1194	712	579	468	930	623	371
**Cytophaga**	666	806	371	474	218	351	684	385
**Leadbetterella**	546	675	687	866	199	291	1546	521
**Pseudarcicella**	341	558	288	851	100	191	548	341
**Arcticibacterium**	294	353	216	545	103	275	781	302
**Hymenobacter**	5759	6828	3762	3421	2231	5836	3263	2055
**Pontibacter**	1304	1480	1213	704	323	1234	676	391
**Rufibacter**	1100	1236	656	628	284	985	772	443
**Echinicola**	632	730	466	844	195	601	1097	336
**Cyclobacterium**	591	801	1098	573	215	568	1865	651
**Algoriphagus**	576	679	382	859	185	558	1262	408
**Belliella**	474	1056	582	1554	308	545	5166	861
**Aquiflexum**	318	426	292	402	114	290	523	299
**Flammeovirga**	752	962	539	1332	218	595	2050	1301
**Marivirga**	306	372	265	387	76	190	657	421
**Chryseolinea**	591	739	458	296	223	510	342	205
**Persicobacter**	398	478	283	356	87	382	351	252
**Bernardetia**	351	588	401	581	132	296	1367	775
**Candidatus Cardinium**	122	82	325	33	22	84	100	53
**Candidatus Amoebophilus**	66	60	31	36	26	46	141	66
**Sphingobacterium**	5795	5867	2095	5339	1540	2236	10235	3261
**Pedobacter**	5788	14743	4638	5027	2239	3295	9142	3461
**Mucilaginibacter**	3256	4421	2758	2628	1039	2355	3565	1508
**Pseudopedobacter**	656	855	2169	1136	274	458	1092	536
**Solitalea**	570	669	523	1030	157	469	1193	621
**Niabella**	3011	3511	1921	1448	675	1296	2238	683
**Chitinophaga**	2044	3027	2278	1391	651	1241	2105	761
**Niastella**	1190	2089	1464	648	549	728	842	429
**Arachidicoccus**	1155	1713	989	1031	332	815	1963	1167
**Pseudoflavitalea**	1153	2363	1107	759	344	723	1080	241
**Filimonas**	902	1622	1045	843	311	578	1206	277

**Flavisolibacter**	602	1033	683	413	170	392	529	258
**Rhodothermus**	842	897	509	588	307	931	482	283
**Salinibacter**	555	674	386	384	214	558	362	175
**Haliscomenobacter**	709	769	561	580	218	526	1257	467
**Saprospira**	149	165	779	151	63	125	117	126
**Chlorobium**	1070	840	524	522	282	1134	904	433
**Pelodictyon**	428	383	257	258	142	434	428	155
**Chlorobaculum**	1242	1071	708	625	368	1253	637	422
**Prosthecochloris**	760	672	551	346	185	948	402	296
**Chloroherpeton**	239	168	81	77	43	219	86	77
**Ignavibacterium**	336	209	379	171	70	247	345	182
**Melioribacter**	167	122	120	67	40	177	142	95
**Candidatus Cyclonatronur**	304	248	152	193	62	325	350	174
**Gemmatirosa**	1513	2095	1024	1432	756	1327	1035	413
**Gemmatimonas**	1295	3112	938	1054	545	838	1110	420
**Fibrobacter**	460	407	203	156	124	590	245	105
**Candidatus Cloacimonas**	193	152	72	45	42	170	78	252
**Akkermansia**	175455	101738	30092	34977	53754	204761	104285	32366
**Verrucomicrobium**	1624	1176	803	722	555	1186	673	537
**Opitutus**	2054	1747	990	1179	817	1416	970	424
**Lacunisphaera**	1007	625	595	484	381	628	373	250
**Ereboglobus**	676	473	314	303	194	595	282	185
**Coraliomargarita**	138	140	58	62	35	117	77	52
**Methylacidiphilum**	196	98	64	67	34	130	99	47
**Candidatus Xiphinematob**	51	36	18	12	13	50	20	16
**Planctomyces**	2293	2157	1104	1474	1630	1474	1371	590
**Pirellula**	367	260	245	168	261	233	146	97
**Fuerstia**	284	194	632	137	117	205	153	131
**Rubinisphaera**	274	204	158	121	141	276	150	180
**Thermogutta**	239	186	121	132	76	261	101	80
**Rhodopirellula**	210	197	131	245	119	197	122	110
**Planctopirus**	199	165	106	74	95	152	110	112
**Paludisphaera**	1364	1045	654	862	821	819	818	306
**Singulisphaera**	647	551	350	378	431	338	347	169
**Isosphaera**	278	212	207	182	167	235	187	105
**Gemmata**	2068	1392	1227	1004	1409	1300	1002	675
**Phycisphaera**	762	681	506	519	361	677	407	209
**Sedimentisphaera**	316	197	118	87	70	341	302	143
**Candidatus Kuenenia**	159	186	82	75	48	154	73	103
**Candidatus Protochlamyd**	1024	301	342	61	154	201	131	128

**Parachlamydia**	94	44	22	81	40	47	49	36
**Neochlamydia**	64	37	30	14	13	27	32	24
**Simkania**	196	51	23	99	44	80	79	53
**Waddlia**	64	45	38	34	22	54	49	98
**Chlamydia**	692	354	571	300	200	521	369	573
**Kiritimatiella**	416	363	248	238	158	510	249	149
**Fusobacterium**	22106	3325	2528	1758	1437	5676	4060	5941
**Ilyobacter**	254	212	96	58	57	271	104	215
**Sebaldella**	3088	338	166	163	98	529	246	7971
**Leptotrichia**	1091	756	390	334	244	1099	536	5988
**Sneathia**	369	233	70	120	292	340	227	798
**Streptobacillus**	263	216	68	139	64	224	218	1345
**Treponema**	6149	2703	1745	1592	916	5707	2218	1180
**Sphaerochaeta**	1090	530	358	288	204	1270	450	269
**Spirochaeta**	716	521	300	311	172	723	348	140
**Salinispira**	272	228	86	64	53	310	104	56
**Sediminispirochaeta**	272	151	125	122	81	376	151	69
**Borrelia**	410	503	139	224	115	319	327	392
**Borreliella**	318	190	98	124	74	270	154	314
**Brachyspira**	1287	885	455	517	283	1348	981	975
**Leptospira**	862	864	625	818	292	884	794	810
**Turneriella**	242	138	260	97	70	206	198	87
**Cloacibacillus**	3841	1114	691	415	744	8452	1671	325
**Aminomonas**	1058	335	212	263	141	663	313	156
**Jonquetella**	489	229	143	136	151	458	244	51
**Thermanaerovibrio**	316	225	156	150	100	551	177	84
**Acetomicrobium**	245	93	80	70	28	154	84	40
**Aminobacterium**	163	62	58	37	31	128	107	43
**Thermovirga**	108	55	27	33	23	103	35	36
**Terriglobus**	966	828	563	634	376	974	590	299
**Granulicella**	863	670	887	529	301	753	518	271
**Acidobacterium**	552	488	382	324	205	512	339	170
**Candidatus Koribacter**	454	395	307	313	167	443	240	140
**Candidatus Solibacter**	844	825	613	528	378	765	491	262
**Luteitalea**	1326	2013	946	1201	1374	1099	1159	462
**Chloracidobacterium**	326	292	176	210	143	299	204	129
**Fervidobacterium**	609	356	424	216	346	545	226	165
**Thermosipho**	280	201	237	110	89	319	282	209
**Thermotoga**	489	320	182	140	306	446	347	153
**Pseudothermotoga**	301	160	79	184	89	267	87	86

**Marinitoga**	184	173	83	48	40	225	86	178
**Defluviitoga**	137	106	3054	59	39	123	122	84
**Petrotoga**	122	83	60	32	30	165	66	84
**Mesotoga**	192	72	62	42	29	179	57	23
**Kosmotoga**	181	79	92	27	55	160	92	129
**Candidatus Saccharimona**	621	514	623	809	502	449	525	120
**Candidatus Babela**	91	54	22	13	20	42	24	58
**Candidatus Bipolaricaulis**	118	124	87	82	52	146	90	44
**Vampirococcus**	187	107	93	73	33	221	87	49
**Nitrospira**	1392	1742	5257	1374	630	1361	895	555
**Leptospirillum**	181	174	100	126	69	213	91	136
**Thermodesulfovibrio**	134	90	102	45	29	139	62	63
**Thermocrinis**	177	66	41	35	28	148	47	45
**Hydrogenobaculum**	100	85	15	40	15	80	58	125
**Aquifex**	86	86	26	24	8	111	38	23
**Hydrogenobacter**	32	26	20	6	13	34	17	26
**Sulfurihydrogenibium**	205	187	117	97	122	214	274	138
**Persephonella**	56	48	14	11	23	81	44	34
**Thermosulfidibacter**	126	114	66	96	16	115	61	22
**Desulfurobacterium**	171	72	46	48	26	148	45	89
**Thermovibrio**	76	57	72	25	14	113	36	28
**Geovibrio**	256	185	106	104	58	357	156	67
**Calditerrivibrio**	233	234	109	57	67	222	104	63
**Denitrovibrio**	166	117	40	30	42	187	71	72
**Deferribacter**	146	103	71	59	41	171	66	259
**Flexistipes**	96	211	487	37	22	106	78	85
**Endomicrobium**	249	127	58	66	51	204	96	52
**Elusimicrobium**	103	64	43	30	35	104	59	35
**Thermodesulfobacterium**	162	116	61	219	32	100	188	126
**Thermodesulfatator**	108	118	53	33	20	124	63	50
**Caldimicrobium**	71	31	64	19	19	40	37	50
**Desulfurispirillum**	334	250	126	207	79	290	154	117
**Caldithrix**	308	255	214	108	54	256	169	110
**Dictyoglomus**	247	153	130	171	51	274	223	165
**Caldisericum**	125	40	28	17	17	74	44	72
**Coprothermobacter**	62	18	21	24	7	42	11	17
**Homo**	1861572	1839757	197372	768172	10254514	1374974	623585	154989
**Halorubrum**	1112	1080	870	1197	784	869	847	334
**Salinigranum**	284	274	200	227	159	226	214	96
**Halopenitus**	197	175	145	193	120	196	143	52

**Halohasta**	83	105	81	94	46	84	60	41
**Haloferax**	582	552	497	559	278	537	374	163
**Haloplanus**	458	405	281	572	277	347	346	168
**Halogeometricum**	95	78	49	92	42	80	49	28
**Haloquadratum**	95	54	28	19	20	37	21	35
**Haloarcula**	439	375	321	295	204	389	259	164
**Natronomonas**	397	264	167	269	160	230	208	121
**Halorhabdus**	282	268	185	308	158	214	212	85
**Halomicrobium**	220	159	153	191	127	192	145	55
**Halorientalis**	184	191	169	261	172	209	159	71
**Halobacterium**	692	613	581	632	379	509	443	205
**Halalkalicoccus**	211	168	127	215	87	153	111	48
**Halorussus**	175	151	180	132	93	139	116	63
**Halodesulfurarchaeum**	130	49	86	74	40	114	63	40
**Halanaeroarchaeum**	68	79	61	54	33	43	64	27
**Haloterrigena**	409	404	274	486	237	276	341	152
**Natronolimnobius**	353	397	317	299	213	293	312	131
**Natrinema**	344	331	256	310	241	264	303	119
**Natronococcus**	230	209	216	284	163	135	147	73
**Halopiger**	220	283	152	291	137	168	173	66
**Salinarchaeum**	202	158	147	157	119	180	162	52
**Halobiforma**	200	185	215	341	166	203	188	81
**Natronobacterium**	178	110	96	109	80	86	86	36
**Natrialba**	145	117	80	87	81	113	110	44
**Halostagnicola**	144	129	146	100	73	147	96	53
**Methanosarcina**	1073	812	475	454	331	1242	850	1159
**Methanococcoides**	148	89	72	59	48	133	66	35
**Methanohalophilus**	140	67	55	45	40	142	59	42
**Methanolobus**	76	57	29	25	27	77	41	26
**Methanomethylovorans**	58	75	27	56	16	70	57	35
**Methanosalsum**	58	40	26	16	6	48	38	44
**Methanohalobium**	41	23	18	13	16	42	19	9
**Methanothrix**	362	185	107	212	614	356	124	942
**Methanoculleus**	403	635	278	273	301	415	209	107
**Methanofollis**	98	103	80	73	75	115	109	32
**Methanolacinia**	94	105	37	46	28	142	54	14
**Methanoplanus**	86	51	59	29	12	72	91	26
**Methanoregula**	225	114	119	101	155	254	101	92
**Methanosphaerula**	76	95	51	35	36	102	52	327
**Methanolinea**	71	38	28	62	33	57	23	14

**Methanocorpusculum**	124	73	52	90	17	172	67	48
**Methanospirillum**	49	66	14	50	43	40	27	34
**Methanocella**	386	273	194	161	172	347	223	102
**Methanobrevibacter**	2632	4260	2937	2841	9933	25590	37895	7644
**Methanobacterium**	571	348	242	426	727	844	466	784
**Methanosphaera**	176	128	51	60	44	238	182	130
**Methanothermobacter**	105	82	33	85	43	141	58	79
**Methanothermus**	37	17	12	8	7	38	20	21
**Methanococcus**	422	279	385	157	105	460	415	382
**Methanothermococcus**	55	21	11	13	12	31	38	19
**Methanocaldococcus**	208	152	69	180	53	168	312	234
**Methanotorris**	29	18	13	6	9	38	25	61
**Thermococcus**	1136	934	576	432	330	1428	551	400
**Pyrococcus**	137	80	64	68	45	157	80	83
**Palaeococcus**	39	35	13	23	7	37	25	28
**Methanomassiliicoccus**	393	126	25	22	33	253	162	9
**Candidatus Methanoplasn**	30	16	14	13	11	32	12	5
**Candidatus Methanometh**	69	95	43	42	17	160	52	11
**Picrophilus**	46	24	15	14	12	33	44	13
**Thermoplasma**	34	23	16	11	9	23	14	28
**Cuniculiplasma**	29	44	11	14	6	50	22	49
**Ferroplasma**	14	15	41	8	11	95	9	20
**Aciduliprofundum**	57	34	20	19	15	61	75	22
**Archaeoglobus**	103	69	43	46	34	145	60	57
**Geoglobus**	60	50	48	57	27	79	24	20
**Ferroglobus**	22	24	7	8	4	19	9	5
**Methanopyrus**	22	30	12	51	10	33	10	6
**Sulfolobus**	107	74	69	65	28	91	61	123
**Sulfodiicoccus**	81	31	11	16	11	17	42	8
**Acidianus**	77	91	28	48	34	86	97	81
**Metallosphaera**	55	37	45	16	13	56	29	26
**Sulfurisphaera**	49	40	12	19	9	51	28	38
**Saccharolobus**	18	20	19	13	6	36	8	23
**Pyrobaculum**	75	50	27	94	26	83	43	36
**Thermoproteus**	55	39	47	25	14	47	38	26
**Vulcanisaeta**	36	17	2	14	9	38	14	50
**Caldivirga**	21	14	15	5	1	9	2	6
**Thermofilum**	71	64	266	35	40	74	35	32
**Staphylothermus**	31	18	8	3	3	23	12	19
**Desulfurococcus**	27	21	12	3	4	23	21	6

**Ignicoccus**	19	15	10	13	7	35	19	8
**Thermogladius**	12	13	11	12	3	12	12	23
**Thermosphaera**	6	7	1	7	0	6	3	11
**Pyrodictium**	38	21	11	11	13	27	15	9
**Pyrolobus**	25	26	14	8	2	20	4	3
**Hyperthermus**	18	9	51	11	3	22	4	32
**Acidilobus**	46	61	22	31	24	58	41	19
**Caldisphaera**	22	18	11	14	14	22	11	51
**Candidatus Nitrosotenuis**	135	91	33	25	24	52	33	17
**Candidatus Nitrosopelagic**	24	10	18	2	7	32	12	44
**Nitrosopumilus**	54	51	16	31	17	62	28	83
**Candidatus Nitrosomarinu**	22	23	15	15	11	21	19	49
**Nitrososphaera**	58	43	59	18	36	52	28	21
**Candidatus Nitrosocaldus**	5	10	6	21	1	8	2	7
**Candidatus Korarchaeum**	10	14	15	5	1	10	6	5
**Candidatus Mancarchaeu**	8	16	11	8	4	18	14	10
**Vequintavirus**	21943	962	218	206	428	388	352	166
**Certrevirus**	6	2	4	5	1	4	6	4
**Seunavirus**	6	5	5	0	12	3	4	13
**Felixounavirus**	3121	273	116	4181	1398	210	407	72
**Mooglevirus**	309	3	4	37	3	10	6	4
**Suspvirus**	29	3	1	5	1	1	3	0
**Kolesnikvirus**	3	10	7	6	6	7	9	1
**Asteriusvirus**	711	13	5	12	24	16	20	34
**Bixzunavirus**	353	36	11	19	27	51	16	7
**Obolenskvirus**	230	53	82	117	460	92	146	118
**Tequatrovirus**	24	273	11	158	11	300	36	83
**Mosigvirus**	7	11	1	0	2	73	222	14
**Dhakavirus**	7	1	2	1	7	70	5	4
**Schizotequatrovirus**	6	4	10	3	2	13	6	5
**Gaprivervirus**	5	7	0	0	0	12	2	6
**Jiaodavirus**	4	1	0	1	1	29	10	8
**Slopekvirus**	2	1	1	1	0	0	5	17
**Karamvirus**	1	1	0	1	1	3	0	7
**Moonvirus**	1	1	2	0	2	36	3	6
**Gelderlandvirus**	1	2	3	47	1	21	6	13
**Pbunavirus**	160	16	2	25	6	26	6	8
**Mieseafarmvirus**	36	10	3	21	110	16	51	24
**Peduovirus**	20	34	7	25	12	17	4	18
**Hpunavirus**	11	4	10	5	3	13	8	76

**Muvirus**	30	0	0	0	1	1	29	0
**Eneladusvirus**	21	3	6	8	3	58	8	17
**Chiangmaivirus**	20	15	12	7	2	35	11	6
**Siminovitchvirus**	17	0	1	0	0	1	0	4
**Phikzvirus**	13	22	3	4	9	9	19	13
**Tegunavirus**	12	4	1	4	2	41	8	18
**Alcyoneusvirus**	9	4	2	1	3	4	5	8
**Agricanvirus**	9	11	11	2	6	17	9	0
**Fletchervirus**	6	2	0	0	2	1	1	11
**Firehammervirus**	3	2	1	1	0	5	1	13
**Sepunavirus**	8	3	0	4	11	3	4	6
**Nazgulvirus**	8	4	2	8	1	0	0	1
**Pakpunavirus**	6	0	4	4	4	2	3	2
**Tulanevirus**	5	4	1	4	1	67	6	22
**Shalavirus**	5	2	1	0	1	3	0	0
**Jilinvirus**	4	2	6	6	4	3	4	9
**Otagovirus**	4	9	6	3	4	9	7	4
**Seoulvirus**	4	2	6	1	0	3	0	3
**Biquartavirus**	3	0	7	2	2	10	1	2
**Vidavervirus**	3	7	3	6	8	3	5	1
**Hapunavirus**	3	7	0	0	3	1	4	8
**Elvirus**	3	0	0	0	0	0	0	0
**Yokohamavirus**	3	2	0	0	0	1	0	2
**Nankokuvirus**	3	0	1	0	1	0	0	1
**Emdodecavirus**	2	4	0	4	5	2	1	1
**Radnorvirus**	2	2	3	0	1	2	2	4
**Erskinevirus**	2	8	0	3	1	1	0	9
**Vhmlvirus**	2	0	1	7	6	0	13	7
**Bcepmuvirus**	2	1	1	0	2	3	2	2
**Bequatrovirus**	1	6	0	1	3	3	1	2
**Viunavirus**	1	1	0	0	4	1	3	2
**Machinavirus**	1	16	4	0	0	4	1	72
**Svunavirus**	1	2	0	5	3	2	0	0
**Skunavirus**	1714	1140	87	72	95	366	243	77
**Cronusvirus**	1150	23	11	18	9	14	24	5
**Barnyardvirus**	649	19	11	10	70	23	158	5
**Liefievirus**	378	517	52	94	865	165	268	73
**Coopervirus**	120	162	243	30	35	73	150	29
**Acadianvirus**	20	29	11	6	17	11	26	3
**Pipefishvirus**	16	25	12	6	7	16	12	9

**Rosebushvirus**	16	14	6	9	6	34	24	7
**Pegunavirus**	13	22	5	2	10	7	12	9
**Kagunavirus**	139	7	7	4	68	5	3	32
**Jerseyvirus**	47	7	3	9	65	97	10	21
**Cornellvirus**	11	0	0	0	2	1	2	2
**Ceduovirus**	197	159	127	116	51	179	234	28
**Fromanvirus**	178	109	61	79	106	98	114	78
**Pamexvirus**	177	512	42	53	82	26	14	20
**Septimatrevirus**	142	17	8	656	10	21	50	211
**Efquatrovirus**	141	31	21	23	741	27	39	2
**Trigintaduovirus**	138	25	2	3	158	53	24	3
**Phicbkvirus**	95	53	27	29	19	25	28	12
**Timquatrovirus**	93	46	50	47	41	54	64	38
**Cheoctovirus**	77	50	88	60	35	29	40	19
**Bronvirus**	69	22	24	8	16	46	21	11
**Moineauvirus**	67	54	4	14	104	119	28	9
**Roufvirus**	52	113	75	87	14488	190	134	72
**Cequinquevirus**	40	11	2	8	2	48	16	3
**Patiencevirus**	39	7	2	47	8	13	33	3
**Wizardvirus**	37	5	1	12	10	7	1	0
**Brussowvirus**	35	37	21	4	173	29	47	10
**Rtpvirus**	10	14	6	22	54	27	29	6
**Rogunavirus**	9	5	1	2	22	8	5	2
**Tlsvirus**	6	1	0	3	8	9	24	6
**Webervirus**	5	6	2	2	10	12	9	7
**Buttersvirus**	17	4	2	6	7	9	5	1
**Charlievirus**	6	7	0	0	3	2	4	1
**Redivirus**	4	6	0	1	7	4	1	0
**Mapvirus**	30	18	4	2	12	16	13	5
**Smoothievirus**	27	2	2	2	2	5	4	1
**Chivirus**	25	10	10	33	3	12	10	12
**Phayoncevirus**	19	5	1	2	7	1	6	4
**Fishburnevirus**	3	6	3	2	4	6	3	1
**Nymphadoravirus**	16	8	1	3	4	4	17	0
**Baxtervirus**	6	5	1	0	4	2	2	1
**Pepyhexavirus**	21	94	4	42	10	19	61	12
**Tinduovirus**	21	3	4	11	54	38	11	2
**Abidjanvirus**	18	4	14	3	4	11	4	2
**Phietavirus**	18	2	1	13	2	2	5	0
**Nipunavirus**	16	7	38	3	7	5	3	10

**Chenonavirus**	15	6	1	7	14	6	5	0
**Nonagvirus**	15	4	0	30	2	5	0	5
**Likavirus**	12	6	17	9	4	3	19	5
**Camvirus**	2	6	10	4	1	2	9	0
**Arequatrovirus**	1	1	0	1	1	1	1	1
**Pahexavirus**	14	4	5	6	5	3	5	2
**Corndogvirus**	14	7	2	6	29	1	17	14
**Seuratvirus**	13	5	7	2	2	24	9	3
**Emalynvirus**	13	0	1	7	6	4	4	0
**Yuavirus**	12	6	3	10	40	1	8	3
**Biseptimavirus**	12	1	0	0	0	2	2	0
**Minunavirus**	12	20	4	39	9	4	19	1
**Betterkatzvirus**	11	25	5	7	5	4	2	0
**Tequintavirus**	11	11	1	35	6	23	406	108
**Brujitavirus**	11	7	1	6	14	2	8	1
**Phifelvirus**	10	0	1	16	10	11	4	0
**Vividuovirus**	10	6	1	2	1	4	3	2
**Woodruffvirus**	8	9	1	9	4	2	6	4
**Kostyavirus**	8	3	3	2	4	3	3	3
**Casadabanvirus**	7	6	3	2	0	1	3	0
**Gordtnkvirus**	7	6	2	2	1	7	0	0
**Sextaecvirus**	7	0	0	3	4	4	11	4
**Omegavirus**	6	4	10	8	7	4	9	2
**Lambdavirus**	6	0	0	0	0	0	0	0
**Hedwigvirus**	6	3	0	7	0	2	2	0
**Unahavirus**	6	1	2	5	0	0	32	17
**Ahduovirus**	6	5	6	1	1	3	2	0
**Xipdecavirus**	5	2	1	0	1	13	1	0
**Dhillonvirus**	5	21	2	1	13	53	3	10
**Gilesvirus**	5	3	0	1	81	6	1	0
**Bantamvirus**	5	6	5	26	8	2	1	1
**Hawkeyevirus**	5	1	0	1	0	1	0	1
**Soupsvirus**	4	6	2	0	1	13	0	3
**Titanvirus**	4	6	3	4	0	0	110	16
**Papyrusvirus**	4	5	0	0	10	0	2	1
**Woesvirus**	4	1	2	5	2	3	3	1
**Nyceiraevirus**	4	0	1	3	3	0	4	0
**Andromedavirus**	4	0	1	0	0	2	1	2
**Attisvirus**	4	0	2	0	1	4	0	0
**Hendrixvirus**	4	8	4	2	5	7	12	14

Wildcatvirus	3	0	0	0	5	2	2	0
Bongovirus	3	1	1	0	2	2	2	1
Vendettavirus	3	0	1	0	0	1	0	1
Limdunavirus	3	4	5	0	1	4	3	1
Pulverervirus	3	0	2	1	1	3	0	0
Bernalvirus	3	0	1	1	3	0	1	0
Mudcatvirus	3	2	0	9	1	13	1	3
Cinunavirus	3	1	0	1	0	3	1	0
Eyrevirus	3	0	0	4	5	0	1	1
Stanholtvirus	3	1	0	1	0	0	1	1
Beetrevirus	2	2	0	2	0	1	1	1
Bendigovirus	2	0	0	1	0	0	1	1
Bowservirus	2	0	0	2	4	0	0	0
Chunghsingvirus	2	0	0	1	0	0	0	0
Galunavirus	2	1	0	6	5	1	0	1
Gesputvirus	2	0	0	2	1	0	1	3
Klementvirus	2	13	0	1	0	1	0	0
Yvonnevirus	2	0	1	2	1	0	1	1
Xiamenvirus	2	1	1	1	0	1	0	0
Slashvirus	2	1	1	0	0	2	1	0
Demosthenesvirus	2	3	2	51	2	5	2	1
Decurrovirus	1	0	2	0	0	1	0	1
Helsingorvirus	1	0	0	1	0	0	0	9
Homburgvirus	1	2	4	0	2	1	0	1
Myunavirus	1	1	1	0	0	0	0	0
Lomovskayavirus	1	5	1	5	9	3	1	0
Saphexavirus	1	0	1	0	0	0	1	9
Marvinvirus	1	3	2	1	2	2	5	0
Samistivirus	1	0	0	1	2	1	0	0
Gaiavirus	1	0	1	0	12	3	6	2
Sasvirus	1	0	1	0	0	0	0	1
Pbi1virus	1	0	0	1	3	1	2	0
Steinhofvirus	1	0	0	0	0	2	0	2
Kelleziovirus	1	1	0	0	3	0	1	1
Gamtrevirus	1	1	0	0	0	0	0	0
Jwalphavirus	387	12	23	91	72	41	35	58
Teseptimavirus	181	147	70	83	78	154	275	53
Friunavirus	51	72	35	44	8400	59	71	21
Phikmvvirus	18	9	5	468	12	15	28	7
Pradovirus	13	18	2	65	10	6	10	3

**Zindervirus**	9	5	11	10	513	24	31	27
**Przondovirus**	2	0	0	1	0	2	0	5
**Phimunavirus**	2	10	3	0	2	1	2	81
**Johnsonvirus**	80	2	6	38	11	5	8	31
**Gamaleyavirus**	40	6	4	12	71	6	11	21
**Rosenblumvirus**	4	1	0	1	7	1	3	2
**Salasvirus**	1	0	0	0	0	4	2	1
**Lessievirus**	23	19	23	10	21	10	20	17
**Lederbergvirus**	22	2	0	0	2	4	2	1
**Litunavirus**	21	7	2	16	0	7	16	6
**Luzseptimavirus**	11	1	3	6	2	5	12	4
**Kochitakasuvirus**	8	1	11	8	2	3	8	1
**Pagevirus**	4	0	0	0	2	4	0	1
**Ithacavirus**	4	2	0	0	7	6	8	20
**Hollowayvirus**	4	0	0	3	6	6	6	4
**Bruynoghevirus**	4	0	0	0	0	1	3	1
**Rauchvirus**	3	1	1	0	1	2	0	0
**Fipvunavirus**	3	5	1	2	3	2	28	48
**Kuravirus**	2	2	2	0	1	1	1	4
**Uetakevirus**	2	1	0	1	1	0	4	0
**Lightbulbvirus**	2	1	1	1	2	2	1	4
**Schmidvirus**	1	0	0	0	1	0	0	0
**Baltimorevirus**	1	0	0	0	0	0	1	0
**Bifseptvirus**	1	1	0	3	2	1	0	1
**Enhodamvirus**	1	0	5	1	0	2	1	0
**Wphvirus**	12	2	9	1	2	14	8	6
**Caeruleovirus**	6	1	3	4	2	1	2	8
**Tsarbombavirus**	5	1	1	0	0	6	2	0
**Bastillevirus**	3	2	0	1	0	4	1	5
**Agatevirus**	1	0	0	0	0	1	0	2
**Silviavirus**	5	2	1	0	1	3	1	8
**Kayvirus**	2	0	0	0	14	4	1	14
**Twortvirus**	1	3	2	1	2	5	4	5
**Okubovirus**	1	2	0	1	1	0	2	0
**Kochikohdavirus**	2	0	0	2	1	0	2	1
**Agtrevirus**	1	0	0	0	0	0	2	1
**Limestonevirus**	1	0	0	0	0	0	4	2
**Kuttervirus**	2	0	7	3	1	5	26	4
**Pandoravirus**	310	269	214	240	147	266	233	119
**Pithovirus**	3	3	3	0	3	3	2	8

**Betabaculovirus**	204	129	25	70	60	111	184	48
**Alphabaculovirus**	91	65	40	50	33	76	100	83
**Gammabaculovirus**	3	0	0	0	1	0	0	0
**Varicellovirus**	65	41	50	35	37	46	114	21
**Simplexvirus**	51	26	13	50	26	22	22	8
**Mardivirus**	7	3	3	3	3	2	3	27
**Iltovirus**	4	1	11	3	1	2	3	1
**Scutavirus**	2	1	0	0	0	0	1	0
**Cytomegalovirus**	31	20	24	15	6	5	19	16
**Muromegalovirus**	16	31	17	8	4	7	17	1
**Roseolovirus**	6	5	5	0	2	2	6	6
**Proboscivirus**	5	3	1	5	2	3	2	4
**Rhadinovirus**	7	5	0	10	4	3	6	4
**Lymphocryptovirus**	4	2	2	0	7	2	2	1
**Percavirus**	3	1	2	1	1	0	1	3
**Cyprinivirus**	19	16	13	18	9	7	10	2
**Batrachovirus**	1	3	14	0	1	2	3	0
**Aurivirus**	3	0	1	1	0	1	2	0
**Betapolyomavirus**	169	7	2	14	6	6	30	4
**Alphapolyomavirus**	6	1	1	0	1	0	0	2
**Nucleorhabdovirus**	5	3	0	0	0	0	0	0
**Almendravirus**	2	0	0	0	0	1	0	0
**Cytorhabdovirus**	1	0	0	0	0	0	0	0
**Rubulavirus**	5	2	2	0	1	1	4	0
**Morbillivirus**	1	0	0	0	0	0	3	2
**Orthobornavirus**	3	0	0	0	0	0	0	0
**Metapneumovirus**	1	0	0	0	1	0	0	0
**Ophiovirus**	1	0	0	0	0	0	0	0
**Tospovirus**	5	11	0	0	0	0	0	0
**Orthobunyavirus**	2	3	1	2	1	2	7	0
**Mammarenavirus**	2	1	0	1	0	3	1	0
**Orthonairovirus**	2	0	0	1	1	0	1	0
**Orthotospovirus**	2	0	1	0	0	0	2	0
**Emaravirus**	1	0	0	1	1	0	0	5
**Tenuivirus**	1	1	0	0	0	0	0	0
**Alphainfluenzavirus**	1	1	0	0	1	0	0	0
**Epsilonarterivirus**	7	0	0	1	0	0	1	0
**Betaarterivirus**	1	1	0	0	0	1	0	0
**Gammaarterivirus**	1	0	0	0	0	0	0	0
**Betacoronavirus**	5	4	3	3	2	7	4	6

**Alphacoronavirus**	3	3	2	6	0	10	0	1
**Deltacoronavirus**	1	1	0	0	0	1	0	0
**Bostovirus**	2	0	0	0	1	0	1	0
**Tobamovirus**	6	2	0	3	0	2	4	3
**Furovirus**	6	0	0	1	0	0	0	2
**Alphaendornavirus**	9	5	4	9	0	8	10	3
**Nepovirus**	2	0	0	0	0	0	0	9
**Fabavirus**	1	0	0	0	0	0	0	0
**Waikavirus**	1	0	0	0	0	0	0	0
**Iflavirus**	1	0	0	1	0	0	0	0
**Kobuvirus**	1	0	0	0	0	0	0	0
**Cosavirus**	1	1	5	0	0	1	3	0
**Robigovirus**	6	0	0	0	0	3	0	2
**Carlavirus**	3	2	0	1	0	0	1	0
**Tymovirus**	1	1	0	0	0	4	0	1
**Aquareovirus**	5	2	0	11	0	2	0	0
**Fijivirus**	1	0	0	0	0	0	0	0
**Orbivirus**	2	4	0	1	2	0	3	2
**Negevirus**	1	0	0	0	0	0	0	0
**Mamastrovirus**	1	1	0	0	0	2	1	1
**Polerovirus**	3	6	1	0	0	1	0	0
**Flavivirus**	2	0	3	9	0	4	3	0
**Potyvirus**	2	2	1	7	1	3	9	1
**Ilarvirus**	2	0	1	0	1	1	0	2
**Velarivirus**	1	0	0	0	0	0	1	0
**Narnavirus**	1	0	0	0	5	0	0	0
**Benyvirus**	1	0	0	0	1	1	30	0
**Picobirnavirus**	1	1	0	0	0	1	0	0
**Polemovirus**	1	0	0	0	0	0	0	0
**Totivirus**	1	0	0	0	0	0	0	0
**Mimivirus**	141	79	63	75	32	103	88	135
**Cafeteriavirus**	8	6	4	4	1	8	5	16
**Parapoxvirus**	24	25	15	16	11	21	13	14
**Molluscipoxvirus**	19	3	1	5	2	2	4	2
**Orthopoxvirus**	17	6	0	1	3	7	6	10
**Avipoxvirus**	7	6	8	5	5	10	6	10
**Suipoxvirus**	7	8	0	0	1	3	3	0
**Cervidpoxvirus**	3	0	0	1	0	0	0	1
**Centapoxvirus**	3	2	1	1	2	2	0	9
**Crocodylidpoxvirus**	2	3	1	2	2	5	2	0

**Capripoxvirus**	1	1	0	1	0	2	0	14
**Leporipoxvirus**	1	3	1	1	1	4	2	0
**Yatapoxvirus**	1	0	0	2	0	0	1	1
**Betaentomopoxvirus**	17	16	4	5	2	8	9	17
**Alphaentomopoxvirus**	7	5	2	1	1	5	2	7
**Prasinovirus**	33	11	8	29	8	13	11	5
**Chlorovirus**	18	25	3	26	8	18	22	17
**Coccolithovirus**	15	11	1	6	1	11	6	5
**Raphidovirus**	5	1	1	2	1	1	2	3
**Prymnesiovirus**	4	6	0	1	4	4	3	13
**Phaeovirus**	3	1	0	2	1	3	0	2
**Alphapapillomavirus**	20	0	0	3	0	8	0	0
**Gammapapillomavirus**	11	1	2	3	0	7	3	0
**Betapapillomavirus**	2	0	0	3	0	1	0	0
**Rhopapillomavirus**	1	0	0	0	0	0	0	0
**Iotapapillomavirus**	1	0	0	0	0	0	0	2
**Mastadenovirus**	25	8	8	16	6	21	15	25
**Aviadenovirus**	5	6	4	5	4	3	8	0
**Atadenovirus**	3	1	0	1	5	3	0	0
**Iridovirus**	9	3	0	4	7	9	4	35
**Chloriridovirus**	5	1	2	3	2	3	7	16
**Ranavirus**	7	2	2	1	1	5	4	2
**Lymphocystivirus**	3	3	2	3	2	3	39	14
**Megalocytivirus**	1	1	0	0	0	0	1	4
**Marseillevirus**	20	14	10	14	8	9	14	6
**Inovirus**	6	0	0	4	0	2	0	0
**Gemycircularvirus**	7	2	1	0	2	24	2	0
**Gemykibivirus**	5	6	18	12	3	1	1	0
**Muscavirus**	8	0	0	1	1	0	0	0
**Glossinavirus**	3	1	0	0	2	1	1	3
**Begomovirus**	8	6	2	2	7	17	7	5
**Ichnovirus**	5	0	0	1	0	1	1	13
**Bracovirus**	5	2	1	0	0	1	0	3
**Alphasphaerolipovirus**	9	1	1	3	2	1	2	3
**Alphanudivirus**	2	1	0	0	0	1	2	0
**Betanudivirus**	2	3	0	3	1	0	0	1
**Rudivirus**	6	1	0	1	0	1	0	1
**Betalipothrixvirus**	1	0	1	0	0	0	2	1
**Badnavirus**	5	6	5	1	0	2	3	8
**Caulimovirus**	2	5	0	0	0	0	0	0

**Whispovirus**	6	5	5	1	2	3	6	0
**Circovirus**	2	8	0	1	0	4	2	1
**Bicaudavirus**	6	8	0	3	1	5	1	1
**Bocaparvovirus**	5	5	4	0	1	4	3	1
**Ambidensovirus**	1	0	0	0	0	0	1	0
**Sputnikvirus**	2	0	0	1	0	0	1	2
**Mavirus**	1	0	0	0	0	0	0	0
**Alphafusellovirus**	1	0	0	0	0	0	0	0
**Betafusellovirus**	1	0	0	0	0	3	1	0
**Alphatectivirus**	2	1	0	0	0	0	6	6
**Nanovirus**	2	0	0	0	0	0	2	0
**Alphaturrivirus**	2	0	0	0	0	0	0	0
**Alphatorquevirus**	1	0	0	0	0	0	1	0
**Ampullavirus**	1	0	0	0	0	0	0	0
**Porprismacovirus**	1	0	0	0	0	0	0	0
**Betasatellite**	1	1	0	0	0	2	0	0
**Drulisvirus**	0	2	1	1	1	4	0	25
**Enquatrovirus**	0	1	0	4	1	2	2	3
**Tunavirus**	0	0	0	0	3	2	1	1
**Ravinvirus**	0	2	204	0	1	4	3	7
**Bignuzvirus**	0	2	1	0	1	2	1	0
**Detrevirus**	0	1	1	0	0	1	1	0
**Psavirus**	0	0	0	0	0	0	0	1
**Cimpunavirus**	0	0	2	0	0	1	0	3
**Coetzeevirus**	0	0	0	0	0	0	0	0
**Ghobesvirus**	0	0	1	0	1	0	0	0
**Krischvirus**	0	2	6	1	0	6	1	7
**Punavirus**	0	1	0	5	0	0	0	4
**Lambdaarterivirus**	0	1	0	0	0	22	0	1
**Phlebovirus**	0	2	0	0	1	7	1	3
**Isavirus**	0	0	0	0	0	0	0	5
**Sigmavirus**	0	0	0	0	0	0	0	0
**Sprivivirus**	0	0	0	0	0	0	0	0
**Hapavirus**	0	0	0	0	1	0	1	0
**Potexvirus**	0	4	0	0	1	0	1	0
**Chrysovirus**	0	0	0	1	0	0	0	0
**Macavirus**	0	10	2	1	2	6	1	2
**Lentivirus**	0	0	0	0	4	0	0	0
**Deltapolyomavirus**	0	0	0	0	0	0	0	0
**Gequatrovirus**	0	0	0	0	0	0	1	0

**Ascovirus**	0	2	1	0	0	1	2	0
**Betatorquevirus**	0	34	0	1	0	0	0	0
**Unaquatrovirus**	0	27	0	0	1	11	1	2
**Rerduovirus**	0	2	0	0	0	0	1	0
**Incheonvrus**	0	2	0	3	0	1	11	24
**Plotvirus**	0	1	0	0	0	0	0	1
**Mardecavirus**	0	2	0	0	0	2	0	0
**Oshimavirus**	0	1	0	11	2	0	1	0
**Nonanavirus**	0	1	1	0	0	1	1	2
**Inhavirus**	0	1	0	0	1	1	0	2
**Eiauvirus**	0	1	0	1	0	1	0	0
**Tijeunavirus**	0	2	0	7	2	1	2	0
**Jimmervirus**	0	1	0	0	0	0	1	0
**Nitunavirus**	0	1	0	0	1	1	11	2
**Krylovvirus**	0	2	2	0	0	3	2	1
**Myxoctovirus**	0	1	2	3	1	2	0	0
**Pecentumvirus**	0	2	0	0	0	11	1	5
**Deltabaculovirus**	0	1	1	0	0	4	0	0
**Ictalurivirus**	0	1	2	2	1	0	0	0
**Herbevirus**	0	1	0	0	0	0	1	0
**Horwuvirus**	0	1	0	0	0	0	0	0
**Shaspivirus**	0	1	0	0	0	0	0	0
**Novirhabdovirus**	0	1	0	0	0	0	0	0
**Avulavirus**	0	1	0	1	0	1	0	0
**Ebolavirus**	0	1	4	0	0	0	1	2
**Yingvirus**	0	1	0	0	0	0	0	1
**Foveavirus**	0	4	0	0	1	0	1	0
**Maculavirus**	0	1	0	0	0	0	0	0
**Gammacoronavirus**	0	1	0	0	0	0	0	0
**Paguronivirus**	0	1	0	0	0	0	0	0
**Ampivirus**	0	1	0	0	1	0	0	0
**Torradovirus**	0	1	0	0	0	0	0	0
**Orthoreovirus**	0	2	0	0	0	0	0	0
**Victorivirus**	0	2	0	0	0	5	3	0
**Pestivirus**	0	1	0	0	0	1	0	0
**Pegivirus**	0	1	0	2	1	1	0	0
**Betatetravirus**	0	1	0	0	0	0	0	0
**Alphavirus**	0	1	2	0	0	0	0	1
**Norovirus**	0	1	0	0	0	0	0	0
**Gammatorquevirus**	0	1	0	0	0	0	0	0

**Solendovirus**	0	1	0	0	0	0	0	0
**Gammaretrovirus**	0	10	1	0	2	1	0	2
**Betaretrovirus**	0	1	0	0	0	0	0	0
**Mastrevirus**	0	1	0	1	1	0	0	0
**Etapapillomavirus**	0	1	0	0	0	0	0	0
**Dyoiotapapillomavirus**	0	1	0	0	1	0	0	0
**Chipapillomavirus**	0	1	0	0	0	0	1	0
**Gammasphaerolipovirus**	0	1	0	0	0	0	0	0
**Cecivirus**	0	0	1	0	0	0	1	0
**Lubbockvirus**	0	0	1	0	0	0	0	0
**Ledantevirus**	0	0	1	0	0	0	1	0
**Dichorhavirus**	0	0	1	0	0	0	0	0
**Tibrovirus**	0	0	1	0	0	0	0	0
**Luteovirus**	0	0	2	1	0	1	0	0
**Orthohepevirus**	0	0	1	0	0	2	0	0
**Pipapillomavirus**	0	0	2	0	0	0	0	1
**Dyomupapillomavirus**	0	0	1	0	0	0	1	5
**Alphatristromavirus**	0	0	1	0	2	0	0	1
**Kleczkowskavirus**	0	0	0	2	0	0	0	0
**Marthavirus**	0	0	0	1	1	0	3	0
**Gorjumvirus**	0	0	0	1	0	0	0	0
**Perisivirus**	0	0	0	2	0	1	0	2
**Rotavirus**	0	0	0	1	0	1	2	1
**Seadornavirus**	0	0	0	1	0	0	0	0
**Orthohantavirus**	0	0	0	2	0	6	1	1
**Sripuvirus**	0	0	0	2	0	0	2	0
**Bymovirus**	0	0	0	1	1	0	0	0
**Trichovirus**	0	0	0	1	0	0	1	0
**Marafivirus**	0	0	0	1	0	1	0	0
**Allexivirus**	0	0	0	1	0	0	0	0
**Siadenovirus**	0	0	0	1	0	0	1	0
**Omikronpapillomavirus**	0	0	0	1	0	0	0	0
**Equispumavirus**	0	0	0	1	0	0	0	0
**Alphatrevirus**	0	0	0	2	11	2	6	0
**Getseptimavirus**	0	0	0	0	1	0	0	0
**Sitaravirus**	0	0	0	0	1	0	0	0
**Cbastvirus**	0	0	0	0	1	0	1	1
**Marburgvirus**	0	0	0	0	1	0	0	0
**Hepatovirus**	0	0	0	0	1	0	1	0
**Mosavirus**	0	0	0	0	1	0	0	0

**Comovirus**	0	0	0	0	1	0	0	0
**Sobemovirus**	0	0	0	0	2	0	0	0
**Invictavirus**	0	0	0	0	2	0	0	0
**Pomovirus**	0	0	0	0	1	0	0	0
**Alphapartitivirus**	0	0	0	0	1	1	3	0
**Vesivirus**	0	0	0	0	1	0	0	0
**Xipapillomavirus**	0	0	0	0	1	0	0	0
**Dyorhopapillomavirus**	0	0	0	0	1	0	0	0
**Amdoparvovirus**	0	0	0	0	1	2	0	3
**Jedunavirus**	0	0	0	0	0	1	0	31
**Traversvirus**	0	0	0	0	0	1	0	0
**Torovirus**	0	0	0	0	0	1	0	0
**Bafinivirus**	0	0	0	0	0	1	1	6
**Wuhivirus**	0	0	0	0	0	1	0	0
**Vesiculovirus**	0	0	0	0	0	4	0	0
**Respirovirus**	0	0	0	0	0	1	0	10
**Anphevirus**	0	0	0	0	0	1	0	0
**Betaendornavirus**	0	0	0	0	0	1	0	1
**Coltivirus**	0	0	0	0	0	1	0	0
**Cypovirus**	0	0	0	0	0	1	2	1
**Oryzavirus**	0	0	0	0	0	1	0	0
**Hypovirus**	0	0	0	0	0	2	0	0
**Ampelovirus**	0	0	0	0	0	1	1	0
**Lineavirus**	0	0	0	0	0	5	2	1
**Erythroparvovirus**	0	0	0	0	0	1	0	0
**Toursvirus**	0	0	0	0	0	3	0	0
**Corticovirus**	0	0	0	0	0	1	0	0
**Enterovirus**	0	0	0	0	0	0	2	3
**Parechovirus**	0	0	0	0	0	0	1	0
**Sopolycivirus**	0	0	0	0	0	0	1	2
**Dinovernavirus**	0	0	0	0	0	0	5	0
**Ipomovirus**	0	0	0	0	0	0	1	0
**Hepacivirus**	0	0	0	0	0	0	1	1
**Rubivirus**	0	0	0	0	0	0	3	0
**Panicovirus**	0	0	0	0	0	0	1	0
**Spiromicrovirus**	0	0	0	0	0	0	2	0
**Deltapapillomavirus**	0	0	0	0	0	0	1	0
**Taupapillomavirus**	0	0	0	0	0	0	1	0
**Epsilonretrovirus**	0	0	0	0	0	0	1	0
**Prosimiispumavirus**	0	0	0	0	0	0	1	0

**Iteradensovirus**	0	0	0	0	0	0	1	1
**Orchidvirus**	0	0	0	0	0	0	0	1
**Cepunavirus**	0	0	0	0	0	0	0	1
**Jonvirus**	0	0	0	0	0	0	0	1
**Megrivirus**	0	0	0	0	0	0	0	1
**Harkavirus**	0	0	0	0	0	0	0	1
**Infratovirus**	0	0	0	0	0	0	0	1
**Amalgavirus**	0	0	0	0	0	0	0	1
**Tombusvirus**	0	0	0	0	0	0	0	1
**Bovispumavirus**	0	0	0	0	0	0	0	1
**Dyoxipapillomavirus**	0	0	0	0	0	0	0	1
**Thetapapillomavirus**	0	0	0	0	0	0	0	1
**Gammapolyomavirus**	0	0	0	0	0	0	0	1
**Chapparvovirus**	0	0	0	0	0	0	0	1
**Deltalipothrixvirus**	0	0	0	0	0	0	0	2
**Iotatorquevirus**	0	0	0	0	0	0	0	2

Table S4

Gene group	CP1	CP3	CP4	CP5	CP6	CP7	CP8	Seafield
aac(3)-I_1_AJ877225	0.00000	29.35374	0.00000	36.41902	2.40914	10.44301	20.17039	29.47549
aac(3)-Ia_1_X15852	8.30881	31.89019	7.02658	49.30890	5.50686	14.03017	24.73817	41.30760
aac(3)-Ib_1_L06157	51.52712	47.49352	1.24699	13.99149	13.97678	31.61560	38.06324	0.00000
aac(3)-Ib-aac(6_)-Ib__1_AF355189	6.03360	1.94023	0.00000	0.00000	0.00000	1.10442	2.10311	0.00000
aac(3)-Ic_1_AJ511268	0.00000	0.00000	0.00000	0.00000	1.18923	1.76742	0.00000	0.00000
aac(3)-IIa_4_L22613	99.97203	28.98846	17.43179	951.20903	42.93649	266.85033	208.66258	18.90360
aac(3)-IIb_1_M97172	11.32049	0.00000	1.63494	0.00000	2.76605	2.39802	0.00000	0.00000
aac(3)-IIIb_2_LLLC01000048	126.17946	15.40683	15.00300	30.56341	66.01921	48.72624	57.21534	0.00000
aac(3)-IIIc_1_L06161	86.62525	18.36439	11.91172	18.03609	32.67402	26.20698	40.88866	0.00000
aac(3)-IVa_1_X01385	0.00000	0.00000	0.00000	0.00000	0.00000	0.00000	0.00000	0.36433
aac(6_)-30-aac(6_)-Ib__1_AJ584652	6.89286	1083.81965	1.90081	62.76470	6.81004	6.46623	59.15806	0.00000
aac(6_)-31_1_AM283489	56.70801	55.35438	6.37911	11.78882	5.66602	6.14853	13.99923	0.00000
aac(6_)-32_1_EF614235	0.00000	0.00000	0.00000	0.00000	0.67282	0.41665	1.34879	0.00000
aac(6_)-aph(2__)_1_M13771	8.45415	2.95443	1.82411	8.05131	4.91846	10.41527	8.37009	0.00000
aac(6_)-Ib-cr_1_DQ303918	1.17578	43.03644	13.84966	342.28918	37.69162	129.52483	322.19043	0.00000
aac(6_)-Ib-Suzhou_1_EU085533	120.25517	1966.62524	42.31476	505.16482	34.89546	170.37656	567.09609	0.00000
aac(6_)-If_1_X55353	0.00000	0.00000	0.00000	0.00000	0.00000	0.00000	1.21473	0.00000
aac(6_)-Ii_1_L12710	0.00000	0.00000	0.53605	11.27729	15.98417	0.00000	0.00000	0.00000
aac(6_)-IIa_1_M29695	19.36426	7.65137	2.25357	5.51207	7.06465	77.16268	15.39204	0.00000
aac(6_)-Im_1_AF337947	0.00000	0.00000	0.00000	0.00000	0.00000	1.20571	0.00000	0.00000
aac(6_)-Iz_1_AF140221	0.00000	0.00000	0.00000	0.00000	0.00000	0.00000	3.43122	0.00000
aadA10_1_U37105	1.53690	1.97435	2.73471	7.24887	1.56710	2.10721	0.52798	7.27555
aadA11_1_AY144590	124.71104	81.62007	51.64048	115.48550	30.93333	55.36531	65.58024	48.15585
aadA13_2_NC010643	3.45957	12.92349	2.39710	4.83761	7.60405	3.12954	3.09010	3.22042
aadA15_1_DQ393783	2.74924	38.77337	5.12862	50.68199	4.73279	11.37140	24.66794	5.42859
aadA16_1_EU675686	2.68056	0.00000	0.00000	1.37755	0.00000	3.82663	0.00000	1.60323
aadA4_1_Z50802	46.36223	7.13957	3.63666	12.55510	13.84342	3.86862	16.63132	1.99048
aadA7_1_AF224733	0.00000	0.00000	1.38294	6.57185	0.00000	0.92727	0.00000	0.00000
aadA8b_1_AY139603	45.82913	2.83829	9.91623	16.42325	3.29261	7.45671	25.29300	2.24802
aadD_1_AF181950	0.00000	0.00000	0.00000	0.00000	0.00000	0.00000	0.00000	0.55879
ampS_1_X80276	0.00000	0.00000	1.48070	4.48939	0.00000	1.16346	1.55088	5.61210
ant(2__)-Ia_5_AY139594	429.10371	982.18550	123.17177	603.57369	1356.26028	958.55538	2059.37018	4.94622
ant(3__)-Ia_1_X02340	71.97034	276.03906	14.49497	176.88704	49.51046	73.79634	128.38703	6.71997
ant(3__)-Ii-aac(6_)-IId_1_AF453998	52.69885	66.58515	12.05065	12.16584	8.14839	17.14351	25.05379	0.00000
ant(6)-Ia_2_KF421157	3.82295	0.00000	0.00000	0.00000	0.00000	0.00000	1.32510	0.41311
ant(6)-Ia_3_KF864551	39.29130	8.29650	3.22463	3.23445	5.27608	28.75140	7.82148	0.90570
ant(6)-Ia_5_AB247327	0.48811	0.95339	0.00000	8.81428	1.48915	1.93334	0.62975	0.00000
ant(6)-Ib_1_FN594949	0.00000	0.40403	0.00000	0.00000	0.00000	1.23974	0.00000	0.00000
ant(9)-Ia_1_X02588	0.00000	0.00000	0.00000	0.18605	0.00000	0.00000	0.00000	0.00000
aph(2__)-Id_1_AF016483	0.00000	0.00000	0.00000	0.00000	0.00000	0.00000	0.00000	1.41826
aph(2__)-If_1_KF652097	0.00000	0.00000	0.00000	0.00000	0.00000	0.00000	2.07219	0.00000
aph(3__)-Ib_1_M28829	90.62662	48.55954	20.08600	261.54656	70.94466	79.32271	92.72321	33.69524
aph(3_)-Ia_2_EU287476	102.70608	284.52367	38.85993	178.34610	408.76846	209.75786	177.26900	1.70591
aph(3_)-Ib_2_AJ744860	1.93329	0.00000	0.00000	0.00000	0.00000	1.53025	0.70152	0.00000
aph(3_)-IIa_2_V00618	3.41062	2.23472	3.97939	1.14525	4.69707	0.87260	2.32632	0.00000
aph(3_)-IIb_1_X90856	0.00000	5.47687	0.00000	1.08309	0.00000	0.00000	0.00000	0.00000
aph(3_)-IIc_1_AM743169	0.00000	0.79948	0.00000	0.00000	0.00000	0.00000	0.00000	0.00000
aph(3_)-III_1_M26832	1.17821	2.45274	0.64781	9.07040	3.28795	5.70096	1.77244	0.35917
aph(3_)-VI_1_KC170992	3.91863	18.27709	0.42446	0.51360	0.71811	24.72468	4.34694	0.86944
aph(3_)-XV_2_GQ626879	0.00000	0.65079	1.88765	0.45581	0.00000	0.00000	0.00000	0.00000
aph(6)-Ic_1_X01702	5.72384	5.24741	0.00000	3.77376	0.00000	2.65592	3.79319	0.00000
aph(6)-Id_2_AF024602	176.37927	104.23454	36.52218	542.84383	144.88907	148.31322	199.62756	69.33660
ARR-3_4_FM207631	1.72501	0.55860	0.33873	0.00000	0.60173	11.49803	2.67610	0.00000
ARR-6_3_JF922883	5.98552	0.28696	0.00000	0.72356	2.57600	44.61414	10.30375	0.00000

**blaACI-1_1_AJ007350**	43.53296	3.04082	0.00000	0.00000	15.83202	2.97499	4.37764	0.00000
**blaACT-14_1_JX440354**	0.00000	0.00000	0.00000	0.00000	0.00000	0.00000	0.00000	0.00000
**blaACT-6_1_FJ237366**	14.67802	0.00000	0.00000	0.00000	6.72456	9.77193	0.00000	0.00000
**blaACT-9_1_HQ693810**	0.00000	0.00000	0.00000	0.00000	0.00000	8.45648	0.00000	0.00000
**blaADC-25_1_EF016355**	0.00000	0.00000	0.00000	0.00000	0.52674	0.00000	0.00000	0.99147
**blaAER-1_1_U14748**	0.00000	1.04186	0.00000	0.00000	0.00000	0.00000	0.00000	1.17026
**blaBEL-2_1_FJ666063**	0.00000	20.14009	2.84964	0.00000	5.42376	0.00000	7.39067	0.00000
**blaBKC-1_1_KP689347**	114.55980	21.25186	34.79459	169.25953	11.44631	51.30441	125.97821	0.00000
**blaCARB-16_1_HF953351**	3.46247	0.00000	0.00000	0.00000	0.00000	34.95639	3.82904	11.34058
**blaCARB-2_1_M69058**	2.38818	2.99873	0.00000	7.93909	7.75261	3.36055	7.00886	0.00000
**blaCMY-93_1_KF992025**	0.00000	0.00000	0.00000	20.62473	0.00000	0.00000	0.00000	0.00000
**blaCMY-94_1_JX514368**	0.00000	0.00000	10.94796	3.49635	16.58032	5.77191	10.56852	0.00000
**blaCTX-M-60_1_AM411407**	182.31766	2.67083	0.00000	32.92303	0.00000	3.80074	5.42822	0.00000
**blaCTX-M-83_1_FJ214366**	13.67513	0.00000	2.26760	16.46308	6.71371	0.00000	10.85748	0.00000
**blaDES-1_1_AF426161**	0.00000	0.00000	0.00000	0.00000	0.00000	0.00000	1.03875	0.00000
**blaEBR-1_1_AF416700**	0.00000	0.00000	0.00000	0.00000	0.00000	0.00000	0.00000	59.99243
**blaFOX-5_1_AY007369**	0.00000	0.00000	7.39566	19.17625	25.91822	52.68766	41.73452	55.69844
**blaIMP-13_1_AJ550807**	39.08814	40.08300	2.77781	257.82075	52.87058	33.17655	292.60959	0.00000
**blaIMP-6_1_AB753460**	152.75347	8344.83416	90.35207	3540.85297	111.11812	540.86614	2743.77219	0.00000
**blaIMP-68_1_MF669572**	0.00000	0.00000	0.00000	8.96519	0.00000	0.00000	0.00000	0.00000
**blaKPC-28_1_KY282958**	0.00000	0.00000	0.00000	0.00000	0.00000	43.62886	0.00000	0.00000
**blaLCR-1_1_X56809**	0.00000	0.00000	0.00000	0.00000	2.26530	0.47252	0.00000	0.00000
**blaLEN7_1_AJ635425**	10.91318	0.00000	0.00000	0.00000	0.00000	3.59704	0.00000	0.00000
**blaMOX-4_1_FJ262599**	1.67333	5.77002	0.00000	0.28527	0.48749	0.00000	0.84312	16.40199
**blaOKP-B-8_1_AM051157**	0.00000	0.00000	0.00000	0.00000	0.00000	1.18653	3.08109	0.00000
**blaOXA-18_1_U85514**	0.00000	15.49056	0.00000	0.00000	0.00000	6.70255	8.50897	0.00000
**blaOXA-198_1_HQ634775**	106.97053	4.61326	0.00000	5.90828	4.25951	0.00000	2.67887	0.00000
**blaOXA-209_1_JF268688**	0.00000	0.00000	1.38227	0.00000	0.00000	0.00000	15.31847	0.00000
**blaOXA-210_1_JF795487**	231.74254	359.77401	49.62260	186.25233	160.23349	94.24912	581.22010	12.94298
**blaOXA-233_1_KJ657570**	356.90019	196.21192	37.05201	75.05037	55.48289	247.37532	176.69224	11.29885
**blaOXA-275_1_APPJ01000001**	0.00000	0.00000	0.00000	4.43399	0.00000	1.91332	2.34223	14.38689
**blaOXA-285_1_APRY01000059**	0.00000	0.00000	4.84388	0.00000	0.00000	0.00000	0.00000	1.94034
**blaOXA-296_1_APOH01000009**	0.00000	20.77895	62.38595	0.51114	1.31024	0.59500	8.18877	16.74021
**blaOXA-299_1_APQD01000016**	0.00000	1.87043	0.48519	0.00000	0.00000	0.00000	2.11194	0.00000
**blaOXA-334_1_KF203108**	86.55822	488.30516	448.24214	409.78822	278.44336	350.13284	487.78820	235.88505
**blaOXA-347_1_JN086160**	7.76831	4.98971	2.80913	3.57569	3.28155	4.37250	34.42654	0.00000
**blaOXA-37_1_AY007784**	5.66230	0.00000	0.00000	0.00000	0.00000	4.15709	1.53926	1.42594
**blaOXA-427_1_KX827604**	10.23185	11.99117	0.00000	10.58213	10.33356	37.11444	20.10606	65.68406
**blaOXA-437_1_KP410856**	0.00000	46.15769	0.26657	11.56788	79.48649	404.72246	27.12236	0.00000
**blaOXA-444_1_CP010800**	0.00000	0.00000	0.00000	0.84799	0.00000	0.00000	0.00000	0.00000
**blaOXA-45_1_AJ519683**	0.00000	0.00000	12.58597	2.93185	0.00000	0.00000	0.00000	0.00000
**blaOXA-46_1_AF317511**	0.67701	0.00000	2.29627	2.72802	0.00000	0.00000	0.00000	1.38138
**blaOXA-47_1_AY237830**	19.99246	1.92933	8.41080	14.06800	22.69263	27.77083	63.95784	0.68723
**blaOXA-486_1_AY597426**	0.00000	0.00000	0.00000	0.00000	0.00000	0.00000	4.01831	0.00000
**blaOXA-490_1_KU721147**	0.00000	0.00000	0.00000	0.00000	0.00000	0.00000	0.98238	5.66778
**blaOXA-5_1_AF347074**	4.66010	1.67075	0.00000	23.59989	0.46445	0.69026	2.19074	2.75244
**blaOXA-552_1_KY682754**	0.00000	0.00000	0.00000	0.00000	0.00000	0.00000	0.00000	8.37443
**blaOXA-58_1_AY665723**	1147.38412	2331.53401	195.32032	2250.98072	2650.79821	2285.38912	3215.57473	118.12978
**blaOXA-9_1_KQ089875**	3.46586	0.00000	0.00000	0.00000	0.00000	1.79385	0.00000	0.00000
**blaOXY-3-1_1_AF491278**	0.00000	0.00000	0.00000	0.00000	0.00000	0.00000	0.00000	0.00000
**blaOXY-6-4_4_AJ871877**	11.16202	0.00000	9.25439	85.66487	0.00000	16.87020	14.40104	0.00000
**blaPAO_2_FJ666065**	0.00000	0.00000	0.00000	0.00000	0.00000	0.00000	9.29360	0.00000
**blaPER-7_1_HQ713678**	0.00000	0.00000	0.00000	0.00000	0.00000	11.02797	0.00000	0.00000
**blaPLA-4A_1_AY507664**	0.00000	0.00000	0.00000	0.00000	0.00000	0.00000	0.00000	0.00000
**blaRAHN-1_1_GU645205**	0.00000	0.00000	0.00000	0.00000	0.00000	0.00000	0.00000	0.96468
**blaSGM-5_1_NG049987**	0.00000	0.00000	0.00000	0.00000	0.00000	1.17858	0.00000	0.00000
**blaSGM-6_1_NG049988**	5.92994	2.12021	0.00000	2.15321	0.00000	0.00000	2.15457	0.00000

**blaSHV-129_1_GU827715**	105.52083	1.05625	0.55510	3.84689	2.11305	9.01859	31.84311	0.00000
**blaSST-1_1_AB008455**	0.00000	0.00000	0.00000	10.89047	0.00000	0.00000	0.00000	0.00000
**blaTEM-4_1_LK391770**	185.48938	27.23164	42.39038	674.22610	356.23829	119.31872	136.77908	11.19482
**blaTER-2_1_FJ263090**	0.00000	0.00000	0.00000	0.00000	0.00000	0.00000	0.00000	0.00000
**blaVEB-1_1_HM370393**	0.00000	0.00000	0.00000	0.00000	0.00000	10.68834	24.16974	0.00000
**blaVIM-42_1_KP071470**	297.29205	121.94347	42.43807	63.02172	93.82729	146.31697	167.79185	18.09287
**cat_2_M35190**	7.34744	3.19432	1.29695	0.00000	0.89764	16.45348	1.76418	0.57200
**cat(pC194)_1_NC_002013**	0.00000	0.00000	0.28254	0.00000	0.00000	0.00000	0.00000	0.00000
**cat(pC233)_1_AY355285**	0.00000	0.00000	0.00000	0.00000	2.80927	0.35685	0.00000	0.00000
**catA1_1_V00622**	14.86437	0.00000	0.61310	1.71059	1.06084	2.31238	1.86811	0.00000
**catA2_1_X53796**	12.51670	0.00000	0.00000	0.00000	0.00000	0.00000	0.00000	0.00000
**catB1_1_M58472**	0.00000	0.00000	0.00000	0.00000	0.00000	0.00000	0.00000	0.00000
**catB2_1_AF047479**	0.00000	0.00000	0.00000	0.00000	0.58992	0.29224	0.00000	0.00000
**catB3_2_U13880**	4.12771	2.66973	0.17434	1.84109	1.69601	3.43387	14.46929	0.33832
**catB9_1_AF462019**	0.00000	0.00000	0.00000	0.00000	0.00000	0.00000	0.00000	0.33993
**catP_1_U15027**	0.00000	0.00000	0.00000	0.00000	0.00000	0.88938	3.88120	0.00000
**catQ_1_M55620**	3.95885	0.00000	0.00000	0.00000	0.00000	0.00000	0.00000	0.00000
**catS_1_X74948**	5.21045	1.76145	1.27107	0.37011	0.75898	3.57197	1.61100	0.00000
**cepA_6_FR688022**	8.78973	0.00000	0.00000	0.00000	0.00000	0.30729	0.00000	0.00000
**cfr(C)_2_CANB01000378**	92.32861	33.39631	13.32525	0.00000	30.21875	126.89133	44.11879	7.70253
**cfxA4_1_AY769933**	15.36126	28.52895	6.05487	2.03585	12.51489	25.03882	6.83757	0.77593
**cfxA6_1_GQ342996**	24.94644	29.19235	8.64254	1.97453	3.98349	36.35731	6.27793	1.11093
**cmlA1_1_M64556**	0.00000	0.00000	0.00000	0.00000	3.88976	0.33034	0.00000	0.00000
**cmlB1_1_AM296481**	42.28959	91.38679	11.82621	25.05606	197.73276	57.82778	68.83347	0.78942
**cmx_1_U85507**	0.79650	0.40531	1.15739	2.16780	0.00000	0.00000	13.92910	0.00000
**cphA1_4_AY261376**	0.00000	0.00000	0.00000	0.00000	0.00000	1.69272	1.15121	1.58635
**cphA6_1_AY227052**	0.00000	0.00000	0.00000	0.00000	0.00000	1.26954	1.09365	1.25975
**dfrA1_5_EU089668**	2.39214	40.31495	1.00890	55.70420	0.00000	4.19546	15.23537	0.22590
**dfrA10_1_L06418**	0.00000	0.00000	0.91313	0.00000	0.00000	0.00000	0.00000	0.00000
**dfrA12_1_FJ763641**	0.00000	0.00000	0.22160	0.80444	0.00000	0.00000	0.00000	0.00000
**dfrA14_2_Z50805**	3.52283	0.00000	0.00000	0.68888	0.00000	0.58320	0.00000	0.60013
**dfrA16_3_AY878718**	16.64099	0.27425	0.00000	0.30733	0.39390	0.68298	3.43725	0.00000
**dfrA17_8_AM932673**	13.72881	0.27425	0.00000	0.69150	3.44663	0.48784	0.00000	0.30121
**dfrA26_1_AM403715**	0.00000	0.00000	0.00000	0.00000	0.00000	0.00000	0.00000	0.00000
**dfrA28_2_FM877476**	0.00000	0.00000	0.00000	0.00000	0.00000	2.14651	0.00000	0.00000
**dfrA5_2_FJ001870**	1.66410	2.28543	0.62086	0.30733	34.17084	2.53679	14.67798	0.00000
**dfrA7_2_AJ884724**	0.31202	0.00000	0.00000	0.00000	0.00000	0.00000	0.00000	0.00000
**dfrA7_5_AJ419170**	3.54606	4.94134	0.00000	5.04755	4.09449	0.00000	4.09438	1.31500
**dfrB1_1_U36276**	0.00000	3.06933	0.00000	0.00000	0.00000	0.00000	0.00000	0.00000
**dfrB2_1_J01773**	0.00000	0.00000	0.00000	0.00000	0.00000	0.00000	0.00000	0.30121
**dfrB3_2_FM877478**	0.00000	0.00000	0.00000	0.00000	0.00000	0.00000	0.00000	0.45181
**dfrG_1_AB205645**	0.00000	1.56621	0.44321	0.00000	9.84157	2.41453	0.61895	0.00000
**ere(A)_5_FN396877**	30.58136	14.05638	8.28437	5.41844	1.21437	3.64717	17.00498	3.07598
**ere(D)_1_KP265721**	6.79015	4.97944	2.69825	20.95504	3.38571	6.52065	59.32201	12.01402
**erm(A)_1_X03216**	4.44498	0.00000	0.00000	1.79110	0.00000	0.00000	0.00000	0.00000
**erm(A)_2_AF002716**	0.00000	0.00000	0.00000	0.00000	0.00000	0.00000	1.44374	0.00000
**erm(B)_9_AF299292**	121.08273	94.83569	126.13263	151.44252	48.05188	258.66673	156.33775	115.44040
**erm(F)_1_M14730**	284.71511	281.08812	73.29685	84.29571	74.12405	155.89083	842.41715	10.96189
**erm(G)_2_L42817**	2.41464	11.67303	1.50148	0.00000	1.90519	22.65189	2.87569	0.00000
**erm(T)_3_AF310974**	0.00000	0.00000	0.00000	0.00000	29.72097	0.00000	7.90814	1.45686
**erm(X)_1_M36726**	0.00000	0.00000	0.00000	18.65676	2.62047	0.00000	0.00000	0.00000
**erm(X)_3_U21300**	0.00000	0.00000	0.00000	38.42637	12.86379	0.00000	0.00000	0.00000
**floR_2_AF118107**	34.97586	14.83621	16.95496	21.40180	17.97935	74.68133	30.51570	1.35134
**fosA_1_M85195**	0.00000	0.00000	0.43176	1.28236	2.08185	0.00000	1.86059	2.34602
**fosA_2_AGDM01000012**	0.00000	0.00000	0.00000	0.00000	0.00000	1.21125	0.62906	0.00000
**fosA_4_ACWU01000146**	0.00000	0.95585	0.00000	0.00000	0.00000	0.00000	0.00000	0.00000
**fosA_6_ACZD01000244**	23.33124	0.71775	0.95752	2.67153	0.00000	2.29815	12.08693	2.28043

**fosA7_1_LAPJ01000014**	5.47766	0.00000	0.00000	0.86097	0.00000	0.00000	0.00000	0.00000
**fosE_1_AB901041**	0.00000	0.85594	0.00000	0.00000	1.61353	0.00000	0.00000	0.00000
**fosE_2_AY029772**	0.00000	0.00000	0.00000	0.00000	0.52299	0.00000	0.00000	0.00000
**imiS_1_Y10415**	0.00000	0.00000	0.67268	0.00000	0.00000	0.84570	2.47317	1.39863
**lnu(B)_1_AJ238249**	5.27327	0.00000	0.00000	0.00000	3.13503	1.03539	0.00000	5.06094
**lnu(C)_1_AY928180**	4.08334	6.30279	0.59452	1.10361	0.75438	9.34295	2.57977	0.57686
**lnu(G)_1_KX470419**	0.00000	0.00000	0.00000	0.00000	0.34834	0.00000	0.00000	1.64259
**lsa(A)_3_AY737526**	0.00000	0.00000	0.00000	0.00000	3.89756	0.00000	0.00000	0.00000
**lsa(E)_1_JX560992**	31.87001	0.00000	0.00000	0.00000	9.61833	3.92404	0.00000	17.73833
**mcr-3.11_1_MG489958**	0.00000	0.00000	0.00000	0.00000	0.00000	0.00000	0.00000	0.72490
**mcr-3.17_1_MH332767**	0.00000	0.00000	0.00000	0.00000	0.00000	0.00000	0.00000	0.81370
**mcr-4.1_1_MF543359**	0.57606	0.00000	0.00000	0.00000	0.94732	0.00000	0.00000	4.41223
**mcr-5.2_1_MG384740**	0.87042	0.00000	0.00000	0.00000	0.00000	0.00000	0.00000	0.00000
**mcr-9_1_NZ_NAAN01000063.1**	1.67373	0.00000	0.00000	0.85427	0.31694	0.00000	0.57081	0.44065
**mdf(A)_1_Y08743**	145.61773	14.97105	8.59240	16.30438	129.46953	35.55777	52.06933	0.00000
**mef(A)_1_AJ971089**	25.49944	33.22810	8.03377	0.00000	1.07304	13.06172	2.81991	56.96810
**mef(B)_1_FJ196385**	10.18043	0.00000	0.00000	0.00000	0.00000	0.00000	0.00000	0.00000
**mef(C)_1_AB571865**	7.45123	21.87827	12.29212	32.90804	3.05080	5.21419	49.89794	6.24044
**mph(A)_1_D16251**	89.02101	2.29572	6.98369	8.36110	20.40194	24.29786	34.21622	4.09721
**mph(A)_2_U36578**	2.99755	0.00000	0.00000	0.00000	0.00000	0.00000	0.00000	0.00000
**mph(E)_1_DQ839391**	97.48374	668.92414	39.52958	252.01140	319.72544	393.49663	574.43149	265.13631
**mph(F)_1_AM260957**	0.00000	0.00000	1.18533	0.00000	0.00000	0.00000	0.00000	0.00000
**mph(G)_1_AB571865**	6.57319	20.41732	10.51628	29.34098	1.89873	4.49412	50.15391	6.65462
**msr(C)_2_AF313494**	1.83329	0.00000	0.00000	29.17954	52.23172	2.34521	5.50796	0.00000
**msr(D)_3_AF227520**	32.93329	34.62985	19.44844	0.00000	9.56499	37.52880	7.76008	180.51284
**msr(E)_1_FR751518**	652.64288	4246.86017	261.19198	1759.01904	1986.94219	2736.78705	3407.26054	1397.12855
**nimA_1_X71444**	0.00000	0.00000	0.00000	1.50889	0.00000	2.09029	0.00000	0.00000
**nimE_1_AM042593**	0.00000	0.00000	0.00000	0.00000	0.00000	0.00000	0.00000	0.23982
**oqxA_1_EU370913**	31.85985	1.47387	2.12709	6.00790	3.25470	4.64049	11.68247	2.73161
**oqxB_1_EU370913**	69.10917	28.69541	24.17412	39.96505	33.10186	24.34857	38.46162	28.25553
**poxtA_1_MF095097**	0.00000	0.00000	0.00000	22.35667	28.22407	19.58923	0.00000	24.75940
**qepA_1_FJ167861**	0.00000	4.48551	0.00000	0.00000	0.00000	0.00000	0.00000	0.00000
**qnrA6_1_DQ151889**	1.42569	0.00000	0.00000	0.44346	0.00000	0.35196	1.27343	0.00000
**qnrB43_1_JQ349151**	0.00000	0.00000	0.00000	1.41177	0.00000	1.21908	0.40967	0.00000
**qnrB60_1_AB734055**	0.00000	0.00000	0.22813	0.00000	0.00000	0.00000	0.00000	0.00000
**qnrS5_1_HQ631377**	0.00000	0.00000	0.00000	0.00000	0.00000	0.00000	0.00000	0.97806
**qnrVC1_1_EU436855**	0.37518	0.32977	0.00000	0.00000	0.00000	0.00000	0.00000	0.00000
**str_2_FN435330**	0.46454	0.00000	0.17332	0.00000	0.00000	0.00000	0.67425	1.34532
**sul1_38_BX248359**	157.46302	952.21802	27.75928	408.51686	106.16778	234.41568	384.53112	11.73083
**sul2_12_AF497970**	56.07602	112.91549	9.89021	137.47959	49.68677	74.26624	103.87082	3.77772
**tet(32)_1_EU722333**	20.72095	13.20263	2.64401	2.21928	2.91732	31.93976	26.97079	2.50966
**tet(33)_1_AY255627**	0.00000	0.00000	1.86335	0.00000	0.00000	0.00000	1.87072	0.00000
**tet(36)_1_AJ514254**	0.00000	0.00000	1.20516	0.00000	0.00000	0.00000	0.00000	0.00000
**tet(39)_1_KT346360**	71.35601	133.70267	33.76986	545.31157	78.71052	326.12398	712.15679	362.91041
**tet(40)_2_AM419751**	40.05285	11.92421	8.61659	9.18678	17.12643	108.78226	21.49405	1.22777
**tet(42)_1_EU523697**	3.29426	6.19505	0.00000	35.59839	6.09306	0.00000	13.13833	0.00000
**tet(44)_2_FN594949**	6.22966	3.17721	0.00000	0.00000	0.00000	3.89605	3.84696	0.00000
**tet(A)_3_AY196695**	26.50960	15.72450	4.12612	38.86698	12.24193	27.53878	27.01959	19.54650
**tet(B)_1_AP000342**	4.41483	4.09603	0.00000	5.25449	0.00000	7.93802	7.88668	0.00000
**tet(C)_2_AY046276**	56.19911	7.45335	22.08413	27.20679	5.90352	9.04675	13.81166	15.46896
**tet(D)_1_AF467077**	0.00000	1.46267	0.00000	0.00000	0.00000	0.78055	1.18910	0.00000
**tet(E)_1_Y19116**	0.00000	0.00000	0.00000	0.00000	0.00000	0.76400	1.16389	6.54502
**tet(G)_2_AF133140**	32.97755	15.15506	21.04745	24.79461	12.67971	14.52599	30.95085	2.90877
**tet(H)_2_AJ245947**	3.70669	0.50680	0.00000	0.00000	0.00000	2.00909	0.00000	1.55057
**tet(L)_7_X60828**	0.00000	0.00000	0.00000	0.52896	18.77934	14.97926	0.00000	0.00000
**tet(M)_9_X56353**	34.25904	4.80443	29.46999	5.91479	61.52119	45.06627	11.82752	12.09540
**tet(O)_3_Y07780**	73.71729	49.62835	21.78433	17.46975	45.94784	217.00247	55.59285	6.19050

**tet(O/32/O)_2_AJ295238**	8.55028	5.95811	0.57478	1.99167	0.00000	10.76702	0.00000	0.00000
**tet(O/W/32/O)_5_JQ740053**	31.50509	21.39503	8.21942	4.60928	1.16693	69.15463	20.57211	1.28272
**tet(Q)_2_X58717**	197.34849	146.33057	52.16485	21.73006	43.54848	141.69939	51.25066	6.41671
**tet(S/M)_2_AY534326**	1.45447	0.00000	0.00000	20.07570	2.10192	0.00000	1.50427	1.10848
**tet(W)_3_AJ427421**	379.47901	184.95548	66.02108	49.73701	56.19076	990.86006	209.12869	17.55734
**tet(X)_1_GU014535**	85.84011	251.74723	34.67422	160.78047	31.35810	116.35218	268.65610	14.19155
**tet(X)_3_AB097942**	0.00000	0.00000	1.03532	0.00000	4.92635	0.00000	0.00000	0.00000
**VanG2XY_1_FJ872410**	0.68055	0.00000	0.00000	0.00000	0.00000	0.00000	0.72944	0.00000
**VanHAX_2_M97297**	0.00000	1.69537	0.02822	19.30613	58.11818	6.56372	6.18198	0.00000
**VanHBX_1_AF192329**	0.43493	0.00000	0.00000	0.00000	0.00000	5.00262	1.31747	0.00000
**vat(F)_1_AF170730**	0.00000	0.00000	0.00000	0.00000	0.00000	0.00000	0.00000	2.73325
